# Molecular insights into the high-efficiency catabolism of chloramphenicol for soil bioaugmentation by *Nocardia testacea* CS1

**DOI:** 10.1016/j.eehl.2026.100259

**Published:** 2026-06-16

**Authors:** Qilin Wang, Tianzi Yang, Yaqing Liu, Zhuang Ke, Yalin Song, Yongping Shan, Huan Deng, Huan He, Rong Ji, Wentao Jiao, Xin Jin

**Affiliations:** a School of Environment, Nanjing Normal University, Nanjing, 210023, China; b State Key Laboratory of Pollution Control and Resource and Reuse, School of Environment, Nanjing University, Nanjing, 210023, China; c Research Center for Eco-Environmental Sciences, Chinese Academy of Sciences, Beijing, 100085, China; d Key Laboratory of Surficial Geochemistry of Ministry of Education, School of Earth Sciences and Engineering, Nanjing University, Nanjing, 210023, China; e College of Light Industry and Food Engineering, Guangxi University, Nanning, 530004, China; f College of Rural Revitalization, Jiangsu Open University, Nanjing, 210036, China

**Keywords:** Antibiotics, Soil remediation, Transcriptome, Environmental tolerance, Microbial catabolism, Colonization

## Abstract

Screening microorganisms capable of efficiently degrading chloramphenicol (CAP) is critical for remediating CAP-contaminated soils. However, few bacterial strains capable of catabolizing CAP have been identified as suitable for soil application, and the metabolic and genetic mechanisms of CAP degradation remain poorly understood. Herein, *Nocardia testacea* CS1 was isolated for its ability to utilize CAP as the sole carbon source. CS1 exhibited substantial CAP mineralization and broad environmental tolerance across temperatures of 15–50 °C and pH values of 4–11. Integrated analyses of intermediate products, gene clusters, and transcriptomes revealed a proposed CAP catabolic pathway. The chloramphenicol degrading (*chd*) gene cluster was proposed to mediate sequential CAP transformation, including initial oxidation (ChdB), amide hydrolysis (ChdD), β-cleavage (ChdC), and transcriptional regulation (ChdR). Downstream nitroaromatic intermediates were further reduced by PnbAB and assimilated into central metabolism. Bioaugmentation experiments demonstrated effective CAP removal in farmland soils, supported by successful colonization and biofilm formation of CS1. Sustained CAP degradation was accompanied by improved soil ecological health. The degradation capabilities of CS1 extend to thiamphenicol and selected *para*-substituted nitroaromatic pollutants, while no activity was observed toward florfenicol or *ortho*/*meta*-substituted nitrobenzoates, indicating structural selectivity. This study demonstrates substantial CAP mineralization by a soil-derived *Nocardia testacea* strain and identifies a *chd* gene cluster associated with CAP catabolism, providing mechanistic insights for the bioremediation of CAP-contaminated soils.

## Highlights

• *Nocardia testacea* CS1 to use CAP as a sole carbon source was isolated from soil.
• •The degradation pathways of CAP correlating to functional genes of CS1 were proposed.
• •CS1’s intact *chd* gene cluster promised efficient and complete catabolism of CAP.
• •CS1’s excellent environmental tolerance enabled its successful colonization in soils.
• •Augmentation with CS1 improved biodiversity, while remediating CAP-contaminated soil.

## Graphical abstract

**Figure:**
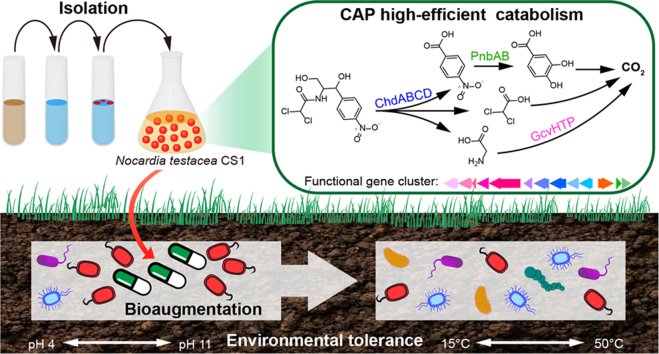

## 1 Introduction

Soil antibiotic pollution increasingly undermines crop production and the health of agricultural ecosystems [1]. As the first mass-produced antibiotic, chloramphenicol (CAP) was widely used in human and veterinary medicine [2]. CAP residues are detected in agricultural soils, primarily due to incomplete metabolism in treated animals and subsequent manure application, as well as wastewater irrigation [3,4]. Reported CAP concentrations in farmland soils generally range from ng/kg to μg/kg levels [5], with occasional hotspot sites reaching mg/kg under intensive livestock production systems [2,6]. In addition to raising the risk of antimicrobial resistance, CAP is potentially carcinogenic, genotoxic, and capable of inducing aplastic anemia in humans, posing significant threats to ecological safety and human health [7]. Hence, there is an urgent need to remediate CAP-contaminated soils.

Microbial degradation is an economical and ecologically friendly approach for removing soil contaminants [8,9]. Screening for highly efficient CAP-mineralizing strains that can resist bacteriostatic effects is essential, as is understanding their metabolic mechanisms for antibiotic resistance. To date, some CAP-resistant bacteria have been identified with metabolic mechanisms such as nitroreduction [10], acetylation [11], hydrolysis [12], and oxidation [13]. However, most of these bacteria cannot completely mineralize CAP, and some even produce more toxic metabolites [14,15]. Besides these co-metabolizing bacteria, only a limited number of bacterial strains have been isolated that can use CAP as the sole carbon source [[16], [17], [18], [19]] (Table S1). Five of these strains, sharing low homology (*i.e.*, *Sphingomonas* sp. CL5.1 [20], *Sphingobium* sp. CAP-1 [21], and *Nocardioides* spp. LMS-CY, QY071, L-11 A [18]), collectively initiate CAP oxidation via glucose-methanol-choline (GMC) family oxidoreductases. However, the reported CAP metabolites vary significantly among them. The complete metabolism pathways, including the initial oxidation of CAP by GMC family oxidoreductase and downstream metabolic transformations, have not yet been fully elucidated. Further investigation is necessary to better understand the genetic mechanisms of CAP metabolism and mineralization, which could lead to more effective bioremediation strategies.

Successful bioaugmentation not only requires screening highly efficient CAP-mineralizing strains but also necessitates effective cell colonization in stressful environments. Temperature and pH are limiting factors that affect biological activity [22]. For example, *Sphingobium* sp. WTD-1, isolated from activated sludge, exhibited optimal CAP degradation at 30 °C and pH 7–8. However, its activity decreased dramatically at temperatures below 25 °C or above 37 °C [19]. In soil environments, the effectiveness of bioaugmentation faces significant challenges, primarily due to the slow mass transfer rates of both nutrients and contaminants, which disadvantage exogenous microbes competing with native soil microorganisms for colonization. Therefore, endowing CAP degraders with strong tolerance to adverse conditions and competitive advantages over native bacterial populations is essential to maintain their high degradation activity in soil environments. Notably, the currently reported several strains capable of mineralizing CAP were mainly isolated from activated sludge (Table S1). Their adaptability to soil environments and their applicability for soil remediation may be limited.

Soils are recognized for their high microbial competition and key roles in antibiotic discovery [23]. The nutrient-deficient nature of soil habitats may drive indigenous bacteria to evolve mechanisms for utilizing antibiotics as alternative carbon and energy sources. This evolutionary adaptation not only enables microbial survival under resource-limited conditions but also provides valuable strains and genetic resources essential for advancing bioremediation technologies. In our previous study, we observed effective ^14^C-CAP mineralization in a soil sample from Changsha, China, implying the presence of soil microorganisms capable of mineralizing CAP [24]. Given their long-term evolution in soil habitats, potential CAP-degrading bacteria originating from soil may have better adaptability in contaminated soils. However, the identity of CAP-degraders and their survival strategies in soils have not yet been revealed. Understanding the metabolic efficiency and environmental adaptability of CAP-degraders to soil conditions would provide a theoretical basis for remediating CAP-contaminated soils.

In this study, a CAP-degrading strain, *Nocardia testacea* CS1, was isolated from soil, and its catabolic performance for degrading amphenicol antibiotics and nitro-aromatic compounds was investigated. Strain CS1 exhibited robust tolerance across a wide pH range (4−11) and extreme temperature conditions (up to 50 °C). Due to its ability to form biofilms via self-aggregation, CS1 successfully colonized CAP-contaminated soils and achieve high remediation efficiency. This study highlights the substantial CAP mineralization capability of this soil-derived *Nocardia* strain, the identification of a *chd* gene cluster associated with CAP catabolism, and its bioaugmentation performance under diverse environmental conditions. These findings provide valuable insights into sustainable and efficient soil decontamination of amphenicol antibiotics and nitroaromatic contaminants.

## 2 Materials and methods

### 2.1 Experimental setup for mineralization of ^14^C-CAP

In a previous study, we assessed CAP mineralization in farmland soils [24]. Five soils with varying CAP mineralization rates were included in the present study. They were originally collected from Changsha (CS, Hunan Province, China), Kunming (KM, Yunnan Province, China), Suzhou (SZ, Jiangsu Province, China), Yingtan (YT, Jiangxi Province, China), and Zhoushan (ZS, Zhejiang Province, China), respectively. Detailed historical records of agrochemical use, manure application, irrigation practices, or specific CAP exposure were not available. The soils were air-dried, sieved (<0.85 mm), and stored under controlled cooling conditions. Their physicochemical properties (pH, soil organic carbon, cation exchange capacity, total nitrogen) were determined as described in Text S1. Before use, these soils were re-activated by adding water to achieve about 20% (w/w) water content and incubating at 15 °C in darkness for 2 weeks.

^14^C-dichloroacetamide-labeled CAP (^14^C-CAP; 93.8 mCi/mmol, purity of 99.7%; Moravek Inc., CA) was used to trace the indigenous microbial mineralization potential of CAP in the five soils. Each soil sample weighing 5 g (dry weight, same as below) was spiked with a certain amount of ^14^C-CAP (25 μg and 600 Bq). The soil water contents were adjusted to 30%–40%, depending on soil texture and organic carbon content [25]. The samples were enclosed in glass flasks and incubated at 30 °C in darkness for 6 days. During this period, a cup of NaOH solution (1 M, 1 mL) was placed inside each flask to capture ^14^CO_2_ and refreshed every two days. The collected NaOH solutions were mixed with a scintillation cocktail (Gold Star Multi-purpose; Meridian Biotechnologies Ltd., UK) at a 1:2 (v/v) ratio, and the radioactivity of ^14^CO_2_ was quantified using a liquid scintillation counter (Tri-Carb 5110 TR, PerkinElmer, Rodgau, Germany). The cumulative ^14^CO_2_ represented the mineralization extent of ^14^C-CAP.

### 2.2 Enrichment, isolation, and characterization of CAP-degrader

Two grams of CS soil were resuspended in 20 mL of mineral salts medium (MSM, pH 7.0; components are provided in Text S2). Subsequent enrichment cultures were acclimated by inoculating 2 mL of the culture into 20 mL of fresh MSM containing 20 mg/L CAP. The liquid cultures were incubated at 30 °C under oxic conditions in darkness with shaking at 150 rpm. After five rounds of acclimation, the CAP-degrading strains were isolated by plating on LB agar and incubating at 30 °C for 20 days. Each colony on the plate was picked and resuspended in fresh MSM with 20 mg/L CAP to assess the CAP-degrading capacity. The CAP-degrading strain, designated CS1, was isolated and deposited in the China Center for Type Culture Collection with the deposit number CCTCC M 2,025,425.

To identify strain CS1, its genomic DNA was sequenced on the NovaSeq PE150 and ONT Nanopore platform by Guangdong Magigene Biotechnology Co., Ltd. (Guangzhou, China). The details of genome assembly are provided in Text S3. The 16 S rRNA gene of CS1 was aligned with those of type strains on the EzBioCloud database [26]. A set of 120 bacterial marker genes was identified and aligned using GTDB-Tk (v2.3.2, release 214) to calculate the average nucleotide identity (ANI) among strains [27]. Phylogenetic trees for marker genes were constructed using IQ-TREE (v2.2.0.3) with 100 bootstraps and the automatic model finder to estimate the best substitution model [28], and visualized in iTOL [29].

To investigate the transcriptomic response of CS1 under CAP exposure, 21 mg of CS1 biomass (dry weight, same as below) harvested from LB broth (without CAP) was inoculated in 50 mL of MSM containing 100 mg/L CAP or glucose, in triplicate. After 35% removal of CAP in the CAP treatment, the biomass pellets were harvested by centrifugation (3 min, 4000×*g*, 4 °C) and immediately frozen in liquid nitrogen. The frozen samples were shipped with dry ice to Guangdong Magigene Biotechnology Co., Ltd. (Guangzhou, China) for RNA extraction and sequencing. Clean reads of transcriptomic data were mapped to the genome of CS1 after processing as described in Text S4. Differential gene expression analysis was performed using the DESeq2 package (v1.40.2) [30] in R, considering genes with |Log_2_(Fold Change)| > 1 and an adjusted *p*-value <0.05 (Benjamini–Hochberg correction) as significantly differentially expressed. All transcriptomic samples were processed in parallel under identical experimental conditions to minimize potential batch effects.

To explore the genetic basis of CAP catabolism, the genome of CS1 was annotated using Bakta [31]. Candidate genes potentially involved in CAP catabolism were identified based on genome annotation and pathway reconstruction. A putative CAP degradation pathway was first inferred from the identified transformation intermediates. Annotated proteins were then screened against the KEGG database to identify enzymes consistent with the proposed metabolic reactions. Transcriptomic responses under CAP exposure were integrated with candidate gene screening to prioritize candidate genes consistent with the proposed metabolic pathway and to delineate the putative *chd* gene cluster associated with chloramphenicol degradation. Further, *Actinomycetes* strains carrying homologs of the *chd* gene cluster were screened from the NCBI database (https://www.ncbi.nlm.nih.gov/datasets/). The screening process is described in Text S5.

To evaluate the biosafety of CS1, Comprehensive Antibiotic Resistance Database [32] and Virulence Factor Database [33] were used to search for suspected antibiotic resistance genes and virulence factors in the CS1 genome, respectively. Antibiotic susceptibility of strain CS1 was evaluated using a modified Kirby–Bauer disk diffusion assay. CS1 cells grown on LB agar were spread onto fresh LB agar plates, and disks containing 0.1 mg trimethoprim and 0.4 mg sulfamethoxazole were placed on the agar surface. Plates were incubated at 30 °C for 96 h, and inhibition zone diameters were recorded. Control disks without antibiotics were included in parallel.

### 2.3 Degradation experiments in aqueous solution

The isolated strain CS1 was first inoculated into LB broth containing 20 mg/L CAP and incubated for 7 days. Bacterial aggregates were harvested by centrifugation at 4000 rpm for 5 min, washed three times with MSM, and resuspended in MSM. For the degradation experiments in aqueous solution, 21 mg of washed bacterial aggregates were inoculated into 50 mL of MSM with initial CAP concentrations ([CAP]_0_) of 20 mg/L or 100 mg/L, and incubated at 30 °C in darkness with shaking at 150 rpm. Notably, strain CS1 grew into biofilms in the form of fluffy pellets with diameters of 1–3 mm in the LB broth (Fig. S1A) and remained intact after centrifugation (Fig. S1B). These pellets were transferred to the MSM unchanged. The inoculated biomass corresponded to an optical density (OD600) of about 0.4, equivalent to a cell density of 10^8^ CFU/mL [34].

To examine the temperature adaptability of strain CS1, the induced CS1 pellets (21 mg, 1–3 mm) were incubated in 50 mL of pre-conditioned MSM (pH 7.0, [CAP]_0_ = 20 mg/L) at temperatures of 5, 15, 25, 30, 35, 40, 50, and 60 °C for 90 min. To assess the pH tolerance of strain CS1, the induced CS1 pellets (10 mg, 1–3 mm) were incubated in 50 mL of MSM with different pH conditions ranging from 3 to 11 (adjusted by HCl or NaOH) at 30 °C for 90 min. All degradation experiments were performed in triplicate.

Samples for metabolite identification were collected when approximately 70% of the initial CAP (100 mg/L) had been removed from MSM (pH 7.0) following inoculation with 21 mg of CS1 biomass. The culture suspensions were freeze-dried. Subsequently, the resulting residues were extracted with methanol. The extracts were concentrated to approximately 1 mL under a gentle stream of nitrogen and filtered through 0.22 μm membranes prior to analysis by high-performance liquid chromatography (HPLC) coupled with quadrupole time-of-flight mass spectrometry (qTOF-MS). Detailed operation information is provided in Text S6. LC–qTOF-MS analysis was primarily performed for qualitative identification of CAP degradation intermediates. Control samples inoculated with heat-inactivated biomass were analyzed to distinguish transformation products from background signals.

In addition to CAP, other compounds, including thiamphenicol (TAP), florfenicol (FF), 2-nitrobenzoic acid (2NBA), 3-nitrobenzoic acid (3NBA), 4-nitrocinnamic acid (4NCN), and 4-nitrobenzoic acid methyl ester (4NME) were subjected to degradation in MSM by CS1 under the same conditions.

### 2.4 Bioaugmentation experiment in soil

Strain CS1 was then used for a bioaugmentation experiment to investigate the mineralization of ^14^C-CAP in different soils. A paddy soil (pH 7.30) from Suzhou (SZ), Jiangsu Province, and a red earth (pH 4.43) from Yingtan (YT), Jiangxi Province were chosen for this investigation (Table S2). The soils were first contaminated with ^14^C-CAP at 5 mg/kg with a total radioactivity of 60 Bq/g soil. In the augmentation treatment, the washed CS1 pellets (1–3 mm, 10 mg dry weight) were inoculated into 5 g (dry weight) of the two CAP-contaminated soils. The soil was thoroughly mixed after inoculation to ensure uniform distribution of soil particles and strain debris. An equal volume of MSM was added to the control treatment. Both treatments were prepared in triplicate. The YT soil was incubated at 30 °C, while the SZ soil was incubated at 15 °C to examine the low-temperature applicability. The experiment was divided into two phases: days 0–30 (Phases Ⅰ) and days 31–38 (Phase Ⅱ). On day 31, an additional 15 μg of ^14^C-CAP (with a radioactivity of 72 Bq/g soil) was added to the bioaugmentation treatments to simulate repeated CAP pollution. Sampling and measurement of ^14^CO_2_ were performed as described in Section 2.1. Meanwhile, on days 0, 7, 31, and 37, 0.3 g of soil was taken from each replicate for analyzing the bacterial community composition (Text S7).

For in situ detection of CS1 cells, soil samples collected on days 7 and 30 were immediately fixed by adding 3 mL of 4% (w/v) paraformaldehyde to 1 g of sample and incubated at 4 °C for 1 h. Subsequently, 1 mL of ice-cold ethanol was added, and the suspensions were stored at −20 °C until further use.

For immobilization, the samples were spread onto glass slides and dried at 46 °C for 15 min. The slides were subsequently dehydrated by immersion in 50%, 80%, and 98% ethanol for 3 min each, followed by air drying. Fluorescence in situ hybridization (FISH) was performed following the previous procedure [35]. Briefly, the immobilized samples were hybridized with the specific probe NTB630 (5′-Cy3-TCACCGCTACACCAGGAATTCCAGTCTCCCCTGAAG-3′, 25 ng/μL) in a hybridization mixture consisting of 1 μL of probe and 8 μL of hybridization buffer (0.9 M NaCl, 0.01% SDS, 20 mM Tris–HCl, pH 7.2, and 20% formamide). Hybridization was carried out in a sealed humid chamber at 46 °C for 90 min. The slides were then rinsed with hybridization buffer to remove excess probe, incubated in the same buffer for 20 min, briefly washed with distilled water, and air dried at room temperature.

For counterstaining of non-target soil microorganisms, the hybridized slides were overlaid with approximately 50 μL of 4′,6-diamidino-2-phenylindole solution (DAPI, 50 μg/mL in PBS). After 10 s of incubation, the slides were gently rinsed with distilled water and air dried at room temperature.

## 3 Results

### 3.1 Mineralization of ^14^C-CAP in the farmland soils

The mineralization rates of ^14^C-CAP in the five farmland soils showed significant differences (Fig. S2). Among them, the CS soil exhibited the highest CAP mineralization activity. After 2 days, the CS soil had mineralized 29.7% of the initial radioactivity, whereas the mineralization ratios of ^14^C-CAP in the other four soils were all below 7.8%. After 6 days of incubation, the CS soil achieved a mineralization ratio of 60.4%, much higher than those reported in previous studies, such as 43.0% within 93 days in a red earth [24], and 38.8% within 64 days in a clay loam [36]. According to our previous study [37], such a high elimination rate of CAP in soil should be attributed to biotic degradation. The key degraders responsible for CAP mineralization needed to be identified.

### 3.2 Isolation, identification, and characterization of CAP-degrader

**Fig. 1:**
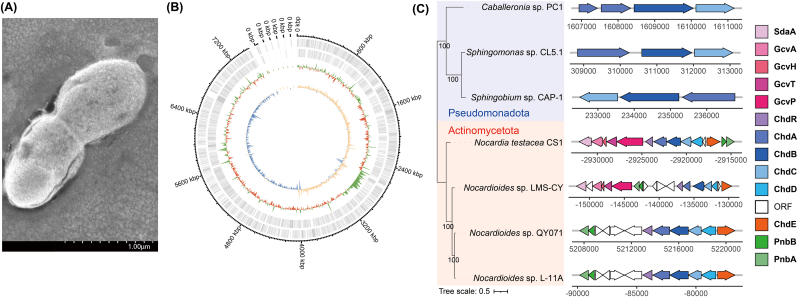
Morphology, genome, and gene cluster for chloramphenicol (CAP) degradation in *Nocardia testacea* CS1. (A) Scanning electron micrograph of CS1. (B) Circular plot of CS1 genome. From outer to inner, the first two rings represent the tRNA, rRNA, ncRNA and coding sequences in forward and reverse strands, respectively. The two innermost rings represent GC content and GC skew, respectively. The GC content ring shows GC variation across the genome using a sliding window, with green and red indicating values above and below the average, respectively. The second innermost ring represents the GC skew in orange and blue. (C) Phylogenetic tree of CS1 and six other reported CAP-degrading strains based on 120 bacterial marker genes created by IQ-TREE (left) and the arrangements of their CAP-degrading gene clusters (right). The accession numbers of their genomes are listed in Table S6. The genes encoding enzymes are as follows: SdaA (L-serine ammonia-lyase); GcvA (glycine cleavage system transcriptional activator); GcvH (glycine cleavage system H protein); GcvT (glycine cleavage system T protein); GcvP (glycine cleavage system P protein); ChdR (IclR family transcriptional regulator); ChdA (aldehyde dehydrogenase family protein); ChdB (GMC family oxidoreductase, synonym—CmO, CapO, and ChloR), ChdC (L-threonine aldolase); ChdD (hippurate hydrolase); ORF (unannotated open reading frame); ChdE (proton-dependent oligopeptide transporter); PnbB (para-hydroxyaminobenzoate lyase); PnbA (para-nitrobenzoate reductase).

During the culture enrichment process, potential CAP-degrading microorganisms were observed as biofilms floating on the medium surface, rather than dispersing in the bulk liquid (Fig. S1C). Following disruption and plating of the biofilm on LB agar, a single CAP-degrading strain was successfully isolated (Fig. S1D). Scanning electron microscopy revealed that the isolate exhibited a short rod-shaped morphology (Fig. 1A).

Genomic analysis indicated a genome size of 7.5 Mb, comprising one chromosome and six plasmids (Fig. 1B). The genome encodes 6127 protein-coding genes, along with 50 tRNAs and 6 rRNA genes. Phylogenetic analysis based on 16 S rRNA gene sequences revealed high similarity to *Nocardia* spp., with *Nocardia testacea* NBRC 100365^T^ being the closest type strain, sharing an ANI of 96.1% (Fig. S3), which exceeds the 95% species threshold [38]. Consequently, the isolate was designated as *N*. *testacea* strain CS1.

### 3.3 CAP-degrading ability and substrate spectrum of CS1

**Fig. 2:**
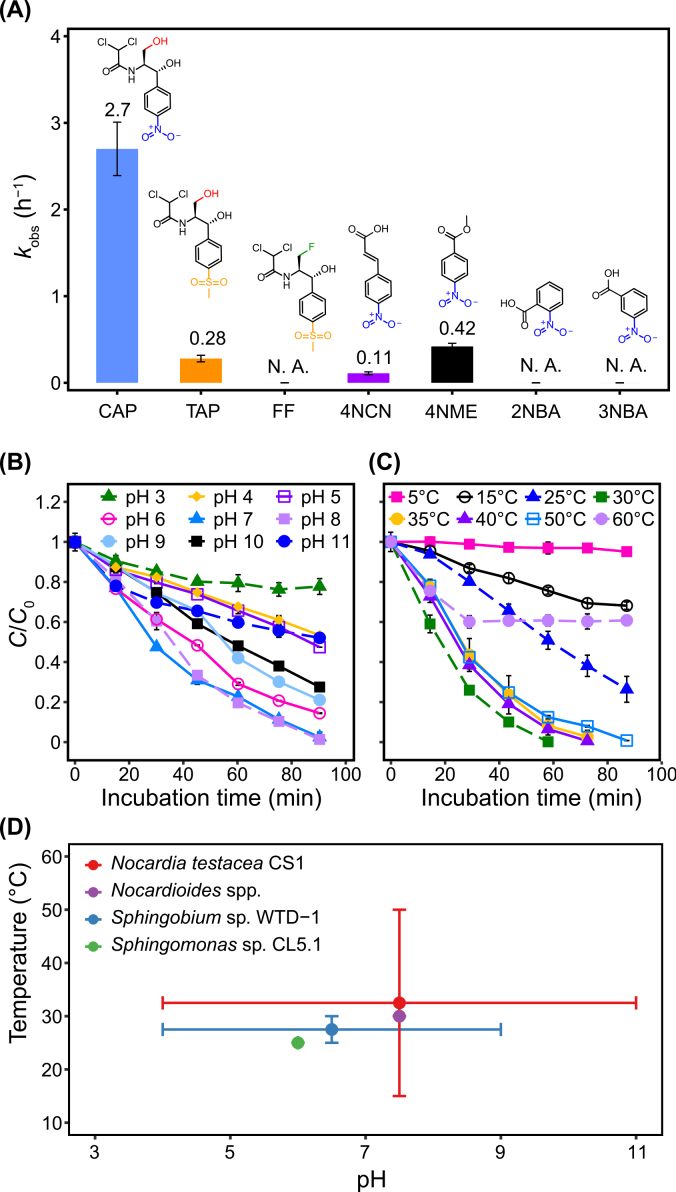
Substrate spectrum and environmental tolerance of CAP degradation by CS1. (A) Degradation rates of amphenicols and nitroaromatic compounds and their molecular structures (N. A.: not applicable). Environmental tolerance: CAP degradation by CS1 in the MSM under varying temperatures (B) and pH conditions (C). Data are means of three individual experiments ± one standard deviation. (D) Comparison of reported temperature and pH ranges for representative CAP-degrading bacteria capable of utilizing CAP as a sole carbon source. Reported temperature and pH ranges for reference strains were compiled from the literature [[17], [18], [19], [20]].

CS1 demonstrated rapid degradation ability for CAP. In MSM, 20 mg/L or 100 mg/L CAP could be completely removed within 1.0 or 2.5 h, respectively (Fig. S1E). Compared to currently reported strains, CS1 exhibited the highest CAP degradation rate (Table S1). Its metabolic capability was also evaluated using two semisynthetic amphenicol antibiotics: TAP and FF. CS1 demonstrated efficient degradation of TAP, with a first-order kinetic constant of 0.28 h^−1^ (Fig. S4A). However, it could not degrade FF, indicating selectivity for specific substrates (Fig. 2A). To further explore the substrate spectrum of CS1, its degradation potential was tested against four nitroaromatic compounds: 2NBA, 3NBA, 4NME, and 4NCN. CS1 could efficiently degrade 4NME and 4NCN, with first-order kinetic constants of 0.42 and 0.11 h^−1^, respectively. In contrast, 2NBA and 3NBA were not significantly degraded (Fig. S4B), indicating that CS1 preferentially metabolizes compounds containing 4-nitrobenzene moieties. The capability of *N*. *testacea* CS1 to effectively degrade CAP, TAP and 4-nitroaromatic compounds suggests its versatility for remediating diverse contaminations or combined pollution.

### 3.4 Environmental tolerance of CS1 regarding pH and temperature

CS1 displayed robust CAP degradation capabilities across a wide range of pH (4−11) and temperature conditions (15–50 °C). Optimal degradation efficiency was observed at pH 7–8 and a temperature of 30 °C (Fig. 2B). Although extreme pH conditions (pH 4 and 11) significantly reduced the degradation rates, CS1 still achieved 46.6% and 47.8% removal of CAP (initial concentration of 20 mg/L) within 90 min, respectively. Under neutral pH conditions, CS1 exhibited optimal degradation activity at temperatures ranging from 30 to 50 °C, achieving complete degradation of 20 mg/L CAP within 90 min (Fig. 2C). Temperature above 60 °C resulted in rapid cell death within 30 min, while low temperature (5–15 °C) gradually suppressed metabolic activity. Nonetheless, CS1 exhibited robust environmental tolerance to various pH and temperature conditions, suggesting its potential for environmental applications.

Compared with previously reported strains, CS1 exhibited a substantially broader temperature (15–50 °C) and pH range (4−11), whereas most reported CAP degraders showed relatively narrow environmental windows. The comparison provides an overview of environmental adaptability under reported experimental conditions and suggests that CS1 possesses strong environmental adaptability, which may be advantageous for bioaugmentation applications.

### 3.5 CAP metabolic pathways in N. testacea CS1

**Fig. 3:**
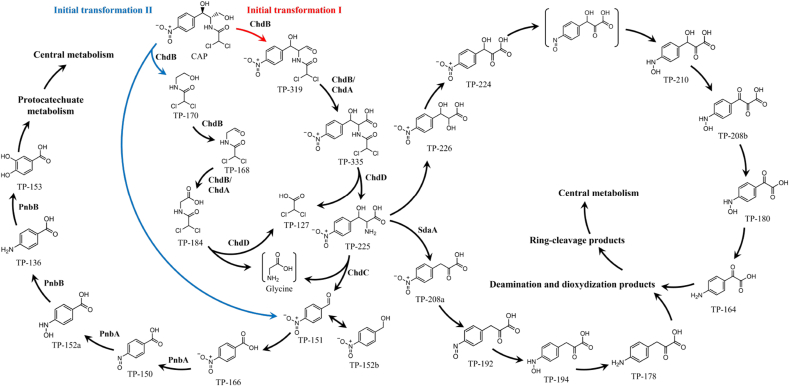
Proposed metabolic pathways of CAP catabolized by CS1, accompanied by the detected metabolites and corresponding key functional enzymes. The compounds in brackets were not detected but were deduced.

The metabolic pathway of CAP in *N*. *testacea* CS1 was systematically elucidated. A total of 24 metabolites were identified, and their mass spectra are presented in Figs. S5–S29. Based on these metabolites, two primary initial transformation pathways of CAP were proposed (Fig. 3). In the first pathway (initial transformation I), CAP was initially converted to 2,2-dichloro-*N*-[1-hydroxy-1-(4-nitrophenyl)-3-oxo-2-propanyl] acetamide (TP-319), which was subsequently oxidized to 2-(2,2-dichloroacetamido)-3-hydroxy-3-(4-nitrophenyl) propanoic acid (TP-335). Hydrolysis of TP-335 likely yielded DCA (TP-127) and 2-amino-3-hydroxy-3-(4-nitrophenyl) propanoic acid (TP-225). Further degradation of TP-225 produced glycine and 4-nitrobenzaldehyde (TP-151). In the second pathway (initial transformation Ⅱ), CAP was directly degraded to 2,2-dichloro-*N*-(2-hydroxyethyl) acetamide (TP-170) and TP-151. TP-170 was further degraded to DCA and glycine through sequential oxidation and hydrolysis. Both pathways converged to produce the same intermediates: TP-151, glycine, and DCA. Glycine was not detected, likely due to its rapid catabolism as a carbon and energy source by CS1.

In the downstream metabolic transformation, nitrobenzene-containing intermediates were further degraded through nitroreduction. Specifically, TP-151 was oxidized to 4-nitrobenzoic acid (TP-166), which was subsequently reduced to protocatechuic acid. Additionally, TP-225, the precursor of TP-151, was also converted into 3-(4-nitrophenyl)-2-oxopropanoic acid (TP-208a) or 2,3-dihydroxy-3-(4-nitrophenyl) propanoic acid (TP-226) (Fig. 3). Both TP-208a and TP-226 underwent nitroreduction to yield aniline compounds, which were further assimilated through central metabolism pathways. This nitroreduction process enables CS1 to not only assimilate nitrobenzene compounds but also mitigate the health risks associated with CAP intermediate metabolites. Additionally, metabolite analysis (Figs. S30–S34) revealed that TAP undergoes a catabolic pathway similar to that of CAP (Fig. S35).

### 3.6 Functional gene integration of CAP catabolism in CS1

**Fig. 4:**
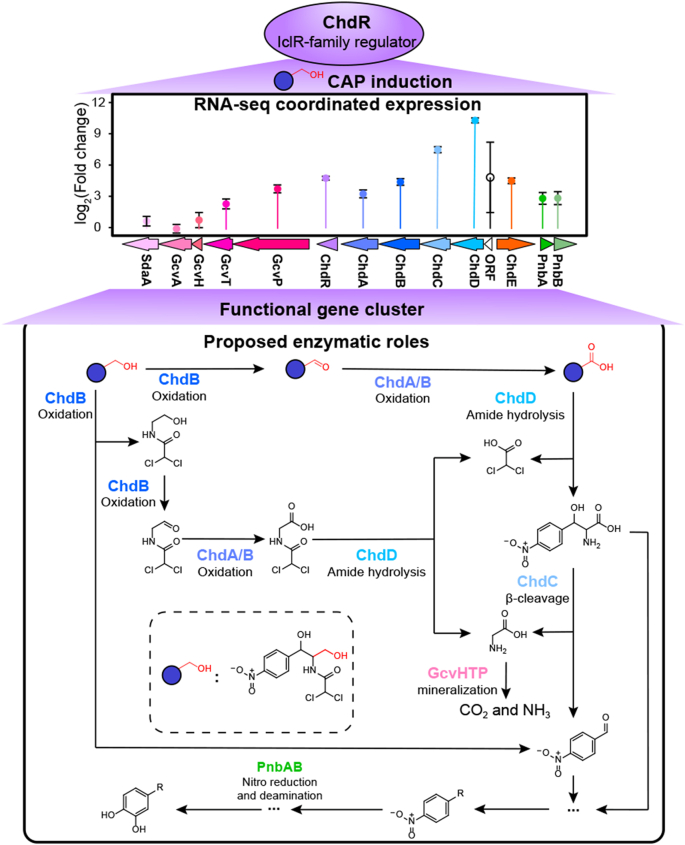
Proposed regulatory and metabolic framework of CAP catabolism in *Nocardia testacea* CS1. ChdR is proposed to regulate transcription of the chd gene cluster, which showed coordinated induction under CAP exposure based on transcriptomic analysis. The encoded proteins catalyze sequential transformation steps in CAP catabolism. Enzyme abbreviations are the same as those defined in Fig. 1.

To elucidate the genetic mechanisms underlying CAP catabolism in CS1, a comparative transcriptomic analysis was conducted. Compared to glucose treatment, 1228 up-regulated genes and 1598 down-regulated genes were observed in response to CAP treatment (Fig. S36A). Notably, a cluster of genes, HKDCAG_13775–HKDCAG_13825, exhibited significant upregulation under CAP treatment (Fig. S36B). Based on metabolic pathway and gene function analyses, the *chd* gene cluster (HKDCAG_13785–HKDCAG_13815), encoding ChdABCDER, was proposed to initiate and accelerate CAP catabolism (Fig. 4). The correlation of these genes to the degradation pathways of CAP is discussed below.

Further genomic database searches identified homologs of the core CAP-degrading genes ChdABCD across five genera within the class *Actinomycetes*: *Nocardia*, *Actinomadura*, *Nocardioides*, *Pseudonocardia*, and *Rhodococcus* (Table S3). These strains were predominantly isolated from diverse environmental matrices, including soils, manure, and compost, indicating that the ecological niche for CAP utilization is widely distributed in natural environments (Table S3).

### 3.7 CS1 bioaugmentation of CAP-contaminated soil

**Fig. 5:**
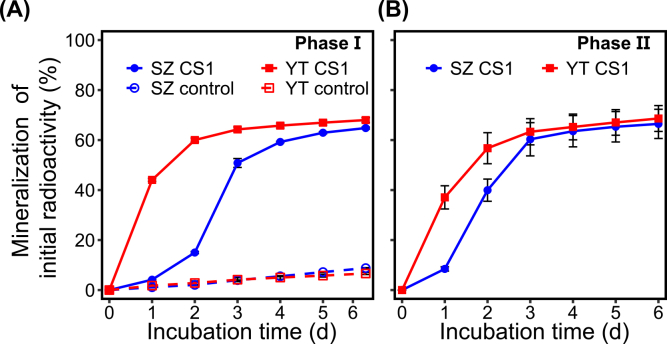
Mineralization of CAP by CS1 in soil and relative abundance of CS1 in soil bacterial communities. (A) The mineralization of ^14^C-CAP in soils with or without CS1 bioaugmentation at phase Ⅰ (within 180 h after initial CAP pollution at the beginning of incubation). (B) The mineralization of ^14^C-CAP in soils with CS1 bioaugmentation at phase Ⅱ (within 180 h after the second round of CAP pollution on the 31st day). Data are means of three individual experiments ± one standard deviation.

The soil remediation potential of *Nocardia testacea* CS1 was evaluated in two CAP-contaminated soils (SZ and YT) with distinct physicochemical properties (Table S2). ^14^C-CAP was utilized to trace CAP mineralization. The YT soil exhibited limited intrinsic CAP mineralization capability, with only 6.6% of the initial radioactivity mineralized over 6 days at 30 °C (Fig. S2). Similarly low levels of ^14^C-CAP mineralization were observed in the non-inoculated control soils, indicating limited indigenous CAP-mineralizing activity. However, upon inoculation with CS1, the mineralization efficiency in YT soil increased substantially to 68.0% under the same conditions. In contrast, the SZ soil demonstrated moderate intrinsic CAP mineralization, with 49.5% of ^14^C-CAP mineralized in 6 days at 30 °C (Fig. S2). However, its CAP mineralization capability was <8.8% at 15 °C in 6 days (Fig. S37), indicating that indigenous CAP-degrading microorganisms are poorly adapted to low temperatures. Remarkably, CS1 inoculation substantially enhanced the low-temperature mineralization capacity of SZ soil, achieving 64.8% ^14^C-CAP mineralization over 6 days at 15 °C (Fig. 5A). These results demonstrate that CS1’s CAP degradation capability is robust across varying soil properties and temperature conditions. To assess long-term application potential, a second phase of ^14^C-CAP was introduced after 30 days. The mineralization rates of ^14^C-CAP in phase II were comparable to, or even slightly faster than, those observed in phase I for both soils (Fig. 5B), indicating that CS1 maintains high degradation efficiency for over one month without decline. The sustained degradation activity suggests successful colonization of CS1 in the bioaugmented soils.

### 3.8 Colonization of strain CS1 in soils

**Fig. 6:**
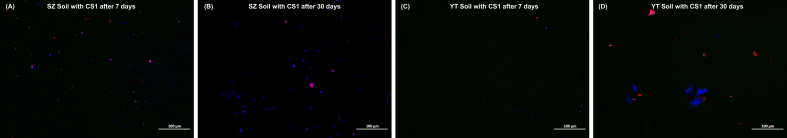
Evaluation of CS1 colonization in SZ and YT soils using dual staining with fluorescent in situ hybridization (FISH) and 4′,6-diamidino-2-phenylindole (DAPI). Red cells indicate *Nocardia testacea* hybridized with the specific probe NTB630 (Cy3-labeled), while blue cells represent all soil microorganisms stained with DAPI. Scale bars represent 100 μm. The relative abundances of *Nocardia testacea* are presented in Fig. S41.

FISH analysis was used to evaluate CS1 colonization in bioaugmented soils. No obvious CS1 biofilms were detected in either inoculated or uninoculated soils (Fig. S38). Although CAP was rapidly degraded following CS1 inoculation, only small aggregates of probed-labeled CS1 cells were observed after 7 days (Fig. 6A and C). In contrast, distinct CS1-associated biofilms were evident in both bioaugmented soils, accompanied by the growth of surrounding microorganisms (Fig. 6B and D). The observed signals indicate the presence of *Nocardia*-like bacteria and are most likely attributable to CS1 under the experimental conditions. These results indicate that CS1 colonizes soils in biofilm form, enabling persistent CAP degradation while providing refuges that support the proliferation of other soil microorganisms under reduced antibiotic pressure.

16 S rRNA amplicon analysis further revealed the impact of CS1 colonization on soil bacteria (Fig. S39). For SZ and YT soils, seven and eight operational taxonomic units (OTUs) with relative abundances >0.1% were identified as biomarkers, respectively (Figs. S40–S41). Among them, OTU_49664, representing *Nocardia* genus members, was present at minimal levels (<2.5%) in the non-inoculated treatments (Fig. S41), indicating low indigenous *Nocardia* populations. In contrast, CS1 maintained high relative abundances in bioaugmented soils after 37 days: 5.6% in SZ soil at 30 °C and 24.3% in YT soil at 15 °C (Fig. S42A–B), consistent with their efficient CAP-degrading performances (Fig. 5A and B).

CAP contamination suppressed five OTUs in SZ soil (belonging to the family Sphingobacteriaceae, genera *Flaviaesturariibacter*, *Sphingomonas*, and *Adhaeribacter*; Fig. S42A), and six OTUs in YT soil (belonging to the phylum WPS-2, order Ktedonobacterales, genera *Methylobacterium*-*Methylorubrum*, and *Bryobacter*; Fig. S42B), with the exception of the *Nocardia* genus (OTU_49664). Antibiotic contamination from CAP inhibited the colonization of indigenous species that shared overlapping niches with CS1. Conversely, bioaugmentation with CS1 protected CAP-sensitive indigenous bacteria from antibiotic stress. In SZ soil, the selected biomarkers were almost fully recovered after bioaugmentation (Fig. S42A). In YT soil, bioaugmentation also increased the relative abundance of OTU_16921 (*Micromonospora*) and OTU_5599 (*Caballeronia zhejiangensis*) (Fig. S42B).

## 4 Discussion

### 4.1 Catabolic metabolism for complete mineralization of CAP

The functions of genes in the *chd* cluster are consistent with the complete catabolic pathway of CAP (Fig. 4). ChdB, sharing 30%, 31%, and 60% identity in amino acid sequences with the known CAP oxidases CapO [20], CmO [21], and ChlOR [18], respectively, is proposed to initiate CAP oxidation via either route I or route II (Fig. 3). By comparison, the substitution of a fluorine atom at the C_3_ position (C_3_-F) in FF completely inhibited its biodegradation, suggesting that the C_3_-OH position in CAP and TAP may serve as a critical binding site for ChdB.

PnbAB has been confirmed to reduce the nitro group in 4-nitrobenzoic acid [39]. Herein, nitroreduction constituted a key downstream step in the CAP metabolic pathway, yielding protocatechuic acid, which was subsequently metabolized via the protocatechuate pathway by PcaHGBLR (Table S4). In contrast, during TAP degradation, the methylsulfonyl group in TAP led to the formation of 4-methylsulfonylbenzoic acid as a dead-end metabolite, resulting in lower carbon and energy yield compared to CAP degradation. This structural impediment explains the slower degradation rate of TAP compared with that of CAP (Fig. 2A). Likewise, it also explains CS1’s ability to utilize 4-nitroaromatic compounds substituted at the 4-position rather than 2- or 3-nitro-substituted counterparts.

In addition, ChdA, annotated as an aldehyde dehydrogenase family protein, likely facilitates the oxidation of CAP or its aldehyde intermediate [13]. The hydrolase ChdD is implicated in the hydrolysis of the amide bond in intermediates, while L-threonine aldolase ChdC is involved in the breakdown of TP-225 via β-cleavage, a process analogous to the conversion of L-threonine to glycine and acetaldehyde [40]. ChdE, a proton-dependent oligopeptide transporter known for its role in drug transport [41], may mediate the uptake of CAP or its metabolites into cells. The regulation of these genes is likely governed by ChdR, which resembles other IclR family regulators associated with aromatic compound catabolism [42]. GcvAHTP and SdaA are involved in glycine metabolism [43,44].

Among the reported CAP-degraders (Fig. 1C), *N*. *testacea* CS1 possesses a relatively complete and compact gene cluster organization, including *chdABCDER*, *pnbAB*, *gcvAHTP*, and *sdaA*. These genes were coordinately induced during growth on CAP (Fig. 4), consistent with an integrated catabolic system, while the presence of the IclR-family regulator ChdR further suggests pathway-specific transcriptional regulation. Such coordinated gene organization and regulation may facilitate efficient metabolic flux transfer, reducing potential bottlenecks, and limit the accumulation of potentially toxic intermediates, thereby improving overall pathway efficiency [45]. Previous studies have shown that GMC family oxidoreductases share conserved catalytic residues and reaction mechanisms [46], while variations in the architecture of substrate-binding pockets may influence substrate accessibility and catalytic efficiency of CAP oxidoreductases [18,20,21]. Together, coordinated gene regulation and potential structural optimization of enzyme–substrate interactions may contribute to the high CAP degradation performance of strain CS1. However, detailed biochemical characterization will still be required to determine the rate-limiting steps and thermodynamic constraints of the pathway.

### 4.2 How CS1 possesses high environmental tolerance

Only a limited number of studies have explored the relationship between CAP degradation performance and environmental conditions. The CAP-degrading activity of *Sphingobium* sp. WTD-1 from activated sludge was nearly halted at 16 °C and 37 °C [19]. We also observed that the CAP-mineralizing performance of indigenous microorganisms in SZ soil was inhibited under low-temperature conditions (Fig. S37). In contrast, strain CS1 maintained robust CAP-degrading performance across a wide temperature range, which may be attributed to the presence of multiple cold shock protein genes in its genome (Table S5). These genes are typically induced under low-temperature stress to overcome reduced efficiency of transcription and translation [47].

Additionally, the exceptional environmental tolerance of CS1 could be attributed to its ability to form biofilms through self-aggregation (Fig. S1C), a well-documented bacterial strategy for mitigating environmental and antimicrobial stress [48]. Consistently, the *bcsB* gene, responsible for biofilm formation [49], is present in CS1’s genome (Table S5). CS1 also carries genes encoding resistance to heavy metals such as copper, chromate, and arsenate (Table S5), further suggesting its potential for stress resistance in soil environments. The presence of multiple stress-response and transport-related genes may contribute to the broad environmental tolerance of strain CS1. However, these genetic determinants are inferred from genome annotation and remain to be experimentally validated.

### 4.3 How CS1 successfully colonizes in contaminated soil

Colonization success is often lower in acidic or alkaline soils compared to neutral soils [50]. However, CS1 demonstrated effective colonization in acidic red earths (YT soil, pH 4.43; Fig. S41B, Table S2), likely due to its biofilm-forming capability [51]. Microorganisms in bioaugmentation processes typically adapt to adhere to clay particles, forming concentrated “hot spots” that enhance pollutant mineralization and improve their survival rates [52,53]. CS1’s ability to form biofilms through self-aggregation in liquid cultures (Fig. S1A) enhances its colonization potential in acid soils. FISH results also confirmed that CS1 grew in the soils in the form of a biofilm (Fig. 6M–P), forming hotspot microdomains that maintained CAP degradation activity.

On the other hand, the survival of invading species positively correlates with their resource utilization capability [54]. The Shannon diversity indices of soil bacterial communities in CAP-contaminated treatments exhibited a continuous decline throughout the incubation period (Fig. S42), owing to antibacterial effects [55]. The reduced biodiversity also facilitated the colonization of CS1. The vacant CAP-degrading niche allowed CS1 to colonize and maintain high abundance (Fig. S39) through its efficient and complete catabolism of CAP. Therefore, the successful colonization of CS1 in soils provides a sustained CAP degradation capacity, which is critical for addressing repeated CAP contamination events from long-term manure application or wastewater irrigation. More importantly, bioaugmentation with CS1 not only effectively eliminated CAP pollution, but also restored soil bacterial species diversity, as evidenced by the significantly elevated Shannon diversity indices in bioaugmented soils (Fig. S43). This dual benefit highlights the potential of CS1 as a sustainable solution for managing antibiotic-contaminated agricultural soils.

The ecological origin of CAP-mineralizing capability in the investigated soils cannot be determined from the current data, particularly in the absence of documented CAP exposure histories. Although pre-existing metabolic functions, such as nitroaromatic catabolism and oxidoreductase promiscuity, may provide a basis for incidental CAP transformation, these general properties alone may not fully explain the occurrence of a relatively integrated CAP-catabolic system. A more specific possibility is that CAP-mineralizing capability arose through the assembly of pre-existing enzymatic modules under ecological interactions with CAP-producing microorganisms. Consistent with this view, comparative genomic analysis identified similarly organized gene clusters in multiple genera within the class Actinobacteria (Fig. S44). This pattern indicates that such gene-cluster organizations may be conserved or recurrently assembled in soil microbial communities. Nevertheless, this evolutionary scenario remains hypothetical and would require further comparative genomic and phylogenetic validation. Consistent with this cautious interpretation, only limited ^14^C-CAP mineralization was observed in non-inoculated soils (Fig. 5A), suggesting that efficient CAP degradation is not widespread but restricted to specific microbial populations such as strain CS1.

### 4.4 Biosafety assessment of CS1 for bioaugmentation

Biosafety considerations are important for the potential environmental application of strain CS1. Some *Nocardia* species are opportunistic pathogens that can cause rare infections, typically treated with trimethoprim–sulfamethoxazole, linezolid, amikacin, or imipenem [56]. Although *Nocardia testacea* has occasionally been isolated from sputum samples of patients with mycobacterial infections [57,58], its pathogenic role remains unclear.

Genome-based and phenotypic analyses suggest a low biosafety risk for CS1. Genome annotation identified no canonical virulence factors and only limited virulence- and resistance-related determinants (Tables S7–S8). Antibiotic susceptibility testing using a Kirby–Bauer disk diffusion assay showed a clear inhibition zone (diameter: 44 mm) with trimethoprim–sulfamethoxazole (Fig. S45), indicating susceptibility to clinically relevant antibiotics.

The potential environmental applications of CS1 should be restricted to controlled conditions with appropriate monitoring to minimize unintended dissemination. If necessary, introduced populations could be reduced using standard microbial inactivation approaches such as drying or chemical disinfection. Overall, CS1 appears to pose low pathogenicity and gene-dissemination risks and represents a manageable candidate for CAP bioremediation under controlled conditions.

## 5 Conclusion

Bioremediation based on functional microbes represents an economic, effective and environmentally friendly method for soil remediation. Currently, only a limited number of CAP-degrading strains have been isolated, and their metabolic mechanisms and potential applications in soil remediation remain poorly understood. In this study, a novel soil strain named *Nocardia testacea* CS1 was isolated and demonstrated its ability to use CAP or TAP as the sole carbon source, exhibiting the highest CAP degradation activity among the known strains. On the other hand, bioaugmentation also faces challenges due to environmental stress, microbial competition, and the complexity of ecosystems. Herein, the soils bioaugmented with CS1 demonstrated successful colonization and sustained CAP-degrading activity, attributed to several reasons: 1) CS1 possesses stress resistance genes that enables it to tolerate diverse adverse conditions; 2) The formation of self-aggregating biofilm enhances its survival in foreign soil environments; 3) The versatile metabolic capabilities of CS1 allow it to occupy available ecological niches. These strategies collectively support the application potential for soil remediation. Specifically, strain CS1 exhibited strong tolerance across a wide pH range (4−11) and temperature conditions up to 50 °C. Its broad pH tolerance enables application in both acidic and alkaline soils. Its low-temperature tolerance makes it suitable for soil remediation under winter conditions. Its high-temperature tolerance suggests potential applications in antibiotics removal during composting. In addition, strain CS1, owing to its versatile metabolic functions, also represents a potential resource for the biodegradation of nitroaromatic compounds, warranting further investigation into its degradation substrate spectrum.

## Data availability

The 16 S rRNA gene, genome sequences, and transcriptomes raw reads of *Nocardia testacea* CS1 are available in the NCBI database under the accession numbers PV194974, PRJNA1079265, and PRJNA1213164, respectively. The 16 S rRNA gene raw reads of soil bacterial communities can be accessed in the NCBI database under the accession numbers PRJNA1219488.

## CRediT authorship contribution statement

**Qilin Wang:** Conceptualization, Data curation, Formal analysis, Investigation, Methodology, Visualization, Writing – original draft, Writing – review & editing. **Tianzi Yang:** Data curation, Formal analysis, Investigation, Software, Writing – original draft. **Yaqing Liu:** Conceptualization, Writing – review & editing. **Zhuang Ke:** Data curation, Investigation, Methodology. **Yalin Song:** Investigation, Methodology, Resources. **Yongping Shan:** Resources, Writing – review & editing. **Huan Deng:** Resources, Writing – review & editing. **Huan He:** Resources, Writing – review & editing. **Rong Ji:** Resources, Writing – review & editing. **Wentao Jiao:** Funding acquisition, Project administration, Supervision, Writing – review & editing. **Xin Jin:** Funding acquisition, Methodology, Project administration, Resources, Supervision, Writing – original draft, Writing – review & editing.

## Declaration of competing interests

The authors declare that they have no known competing financial interests or personal relationships that could have appeared to influence the work reported in this paper.
