# Supplementary material for: Molecular insights into the high-efficiency catabolism of chloramphenicol for soil bioaugmentation by Nocardia testacea CS1
Source: Eco Environ Health. 2026 Jun 16;5(3):100259. doi: 10.1016/j.eehl.2026.100259 (PMC13400229; doi:10.1016/j.eehl.2026.100259)
Supplement: Multimedia component 1 [file mmc1.docx]

**Supplementary information for**

**Molecular insights into high-efficient catabolism of chloramphenicol for soil bioaugmentation by *Nocardia testacea* CS1**

Qilin Wang*^a,b^*, Tianzi Yang*^d^*, Yaqing Liu*^e^*, Zhuang Ke*^f^*, Yalin Song*^d^*, Yongping Shan*^c^*, Huan Deng*^a^*, Huan He*^a^*, Rong Ji*^b^*, Wentao Jiao*^c^*^,^*, and Xin Jin*^a^*^,^*^b^*^,^*

*a*. School of Environment, Nanjing Normal University, Nanjing 210023, China

*b*. State Key Laboratory of Pollution Control and Resource and Reuse, School of the Environment, Nanjing University, Nanjing 210023, China

*c*. Research Center for Eco-Environmental Sciences, Chinese Academy of Sciences, Beijing 100085, China

*d*. Key Laboratory of Surficial Geochemistry of Ministry of Education, School of Earth Sciences and Engineering, Nanjing University, Nanjing 210023, China

*e*. College of Light Industry and Food Engineering, Guangxi University, Daxue Road 100, Nanning 530004, China

*f*. College of Rural Revitalization, Jiangsu Open University, Nanjing 210036, China

* Corresponding authors

Xin Jin: xjin@njnu.edu.cn

Wentao Jiao: wtjiao@rcees.ac.cn

**Number of pages:** 72

**Number of texts:** 7

**Number of figures:** 44

**Number of tables:** 8

**CONTENTS**

[TEXTS 10](#_Toc226626060)

**[Text S1.](#_Toc226626061)** [The measurements of soil properties 10](#_Toc226626061)

**[Text S2.](#_Toc226626062)** [The mineral salt media components 10](#_Toc226626062)

**[Text S3.](#_Toc226626063)** [Genomic assembly 11](#_Toc226626063)

**[Text S4.](#_Toc226626064)** [Transcriptome data filtering 11](#_Toc226626064)

**[Text S5.](#_Toc226626065)** [Homologous genes cluster analysis 11](#_Toc226626065)

**[Text S6.](#_Toc226626066)** [Quantification using high-performance liquid chromatography (HPLC) and metabolite identification using HPLC coupled with high-resolution mass spectrometer 12](#_Toc226626066)

**[Text S7.](#_Toc226626067)** [The 16S rRNA gene amplicon sequence analysis 14](#_Toc226626067)

[FIGURES 15](#_Toc226626068)

**[Figure S1.](#_Toc226626069)** [The morphologies of strain CS1 and its chloramphenicol (CAP) degrading capacity.](#_Toc226626069) **[(A)](#_Toc226626069)** [The aggregation biofilm of CS1 cultured in LB broth at 150 rpm, and](#_Toc226626069) **[(B)](#_Toc226626069)** [the pellets (1–3 mm) after washing with mineral salt medium.](#_Toc226626069) **[(C)](#_Toc226626069)** [Biofilm (marked with red circles) grown at the air-liquid interface after CS soil enrichment acclimated with CAP as the only carbon source.](#_Toc226626069) **[(D)](#_Toc226626069)** [Colonies of strain CS1 grown on LB agar plates for 6 days.](#_Toc226626069) **[(E)](#_Toc226626069)** [Degradation dynamics of 20 and 100 mg/L CAP by strain CS1 at 30°C and pH 7. 15](#_Toc226626069)

**[Figure S2.](#_Toc226626070)** [Mineralization of](#_Toc226626070) ^[14](#_Toc226626070)^[C-Chloramphenicol (](#_Toc226626070)^[14](#_Toc226626070)^[C-CAP) in the five farmland soils from Changsha (CS), Kunming (KM), Suzhou (SZ), Yingtan (YT), and Zhoushan (ZS), respectively. The soil incubation experiment was conducted at 30°C in darkness with shaking of 150 rpm. 16](#_Toc226626070)

**[Figure S3.](#_Toc226626071)** [Phylogenetic tree of CS1with the species greater than 97% identity of the 16S rRNA gene. Accession numbers and identity with the 16S rRNA gene of CS1 are provided in parentheses. 17](#_Toc226626071)

**[Figure S4.](#_Toc226626072)** [Degradation kinetics of CAP structural-like compounds by CS1, including](#_Toc226626072) **[(A)](#_Toc226626072)** [amphenicols of chloramphenicol (CAP), thiamphenicol (TAP), and florfenicol (FF);](#_Toc226626072) **[(B)](#_Toc226626072)** [nitroaromatic compounds of 2-nitrobenzoic acid (2NBA), 3-nitrobenzoic acid (3NBA), 4-nitrocinnamic acid (4NCN) and 4-nitrobenzoic acid methyl ester (4NME). 18](#_Toc226626072)

**[Figure S5.](#_Toc226626073)** [LC–MS](#_Toc226626073) **[(A)](#_Toc226626073)** [and LC–MS/MS](#_Toc226626073) **[(B)](#_Toc226626073)** [spectra of CAP. 19](#_Toc226626073)

**[Figure S6.](#_Toc226626074)** [LC–MS](#_Toc226626074) **[(A)](#_Toc226626074)** [and LC–MS/MS](#_Toc226626074) **[(B)](#_Toc226626074)** [spectra of 2-(2,2-dichloroacetamido)-3-hydroxy-3-(4-nitrophenyl)propanoic acid (compound TP-335). 20](#_Toc226626074)

**[Figure S7.](#_Toc226626075)** [LC–MS](#_Toc226626075) **[(A)](#_Toc226626075)** [and LC–MS/MS](#_Toc226626075) **[(B)](#_Toc226626075)** [spectra of 2,2-dichloro-](#_Toc226626075)*[N](#_Toc226626075)*[-[1-hydroxy-1-(4-nitrophenyl)-3-oxo-2-propanyl]acetamide (compound TP-319). 21](#_Toc226626075)

**[Figure S8.](#_Toc226626076)** [LC–MS](#_Toc226626076) **[(A)](#_Toc226626076)** [and LC–MS/MS](#_Toc226626076) **[(B)](#_Toc226626076)** [spectra of 2,3-dihydroxy-3-(4-nitrophenyl)propanoic acid (compound TP-226). 22](#_Toc226626076)

**[Figure S9.](#_Toc226626077)** [LC–MS/MS spectrum of 2-amino-3-hydroxy-3-(4-nitrophenyl)propanoic acid (compound TP-225). 23](#_Toc226626077)

**[Figure S10.](#_Toc226626078)** [LC–MS/MS spectrum of 3-hydroxy-3-(4-nitrophenyl)-2-oxopropanoic acid (compound TP-224). 24](#_Toc226626078)

**[Figure S11.](#_Toc226626079)** [LC–MS](#_Toc226626079) **[(A)](#_Toc226626079)** [and LC–MS/MS](#_Toc226626079) **[(B)](#_Toc226626079)** [spectra of 3-hydroxy-3-(4-hydroxyaminophenyl)-2-oxopropanoic acid (compound TP-210). 25](#_Toc226626079)

**[Figure S12.](#_Toc226626080)** [LC–MS/MS spectrum of 3-(4-nitrophenyl)-2-oxopropanoic acid (compound TP-208a). 26](#_Toc226626080)

**[Figure S13.](#_Toc226626081)** [LC–MS/MS spectrum of 3-(4-hydroxyaminophenyl)-2,3-dioxopropanoic acid (compound TP-208b). 27](#_Toc226626081)

**[Figure S14.](#_Toc226626082)** [LC–MS/MS spectrum of 3-(4-hydroxyaminophenyl)-2-oxopropanoic acid (compound TP-194). 28](#_Toc226626082)

**[Figure S15.](#_Toc226626083)** [LC–MS/MS spectrum of 3-(4-nitrosophenyl)-2-oxopropanoic acid (compound TP-192). 29](#_Toc226626083)

**[Figure S16.](#_Toc226626084)** [LC–MS/MS spectrum of 2-(2,2-dichloroacetamido)acetic acid (compound TP-184). 30](#_Toc226626084)

**[Figure S17.](#_Toc226626085)** [LC–MS](#_Toc226626085) **[(A)](#_Toc226626085)** [and LC–MS/MS](#_Toc226626085) **[(B)](#_Toc226626085)** [spectra 2-(4-hydroxyaminophenyl)-2-oxoacetic acid (TP-180). 31](#_Toc226626085)

**[Figure S18.](#_Toc226626086)** [LC–MS/MS spectrum of 3-(4-Aminophenyl)-2-oxopropanoic acid (TP-178). 32](#_Toc226626086)

**[Figure S19.](#_Toc226626087)** [LC–MS/MS spectrum of 2,2-dichloro-](#_Toc226626087)*[N](#_Toc226626087)*[-(2-hydroxyethyl)acetamide (TP-170). 33](#_Toc226626087)

**[Figure S20.](#_Toc226626088)** [LC–MS/MS spectrum of 2,2-dichloro-](#_Toc226626088)*[N](#_Toc226626088)*[-(2-oxoethyl)acetamide (TP-168). 34](#_Toc226626088)

**[Figure S21.](#_Toc226626089)** [LC–MS](#_Toc226626089) **[(A)](#_Toc226626089)** [and LC–MS/MS](#_Toc226626089) **[(B)](#_Toc226626089)** [spectra of 4-nitrobenzoic acid (TP-166). 35](#_Toc226626089)

**[Figure S22.](#_Toc226626090)** [LC–MS](#_Toc226626090) **[(A)](#_Toc226626090)** [and LC–MS/MS](#_Toc226626090) **[(B)](#_Toc226626090)** [spectra of 2-(4-aminophenyl)-2-oxoacetic acid (TP-164). 36](#_Toc226626090)

**[Figure S23.](#_Toc226626091)** [LC–MS/MS spectrum of protocatechuic acid (TP-153). 37](#_Toc226626091)

**[Figure S24.](#_Toc226626092)** [LC–MS](#_Toc226626092) **[(A)](#_Toc226626092)** [and LC–MS/MS](#_Toc226626092) **[(B)](#_Toc226626092)** [spectra of 4-nitrosobenzoic acid (TP-152a). 38](#_Toc226626092)

**[Figure S25.](#_Toc226626093)** [LC–MS/MS spectrum of (4-nitrophenyl)methanol (TP-152b). 39](#_Toc226626093)

**[Figure S26.](#_Toc226626094)** [LC–MS/MS spectrum of 4-nitrobenzaldehyde (TP-151). 40](#_Toc226626094)

**[Figure S27.](#_Toc226626095)** [LC–MS](#_Toc226626095) **[(A)](#_Toc226626095)** [and LC–MS/MS](#_Toc226626095) **[(B)](#_Toc226626095)** [spectra of 4-nitrosobenzoic acid (TP-150). 41](#_Toc226626095)

**[Figure S28.](#_Toc226626096)** [LC–MS/MS spectrum of 4-aminobenzoic acid (TP-136). 42](#_Toc226626096)

**[Figure S29.](#_Toc226626097)** [LC–MS](#_Toc226626097) **[(A)](#_Toc226626097)** [and LC–MS/MS](#_Toc226626097) **[(B)](#_Toc226626097)** [spectra of 2,2-dichloroacetic acid (TP-127). 43](#_Toc226626097)

**[Figure S30.](#_Toc226626098)** [LC–MS](#_Toc226626098) **[(A)](#_Toc226626098)** [and LC–MS/MS](#_Toc226626098) **[(B)](#_Toc226626098)** [spectra of thiamphenicol (TAP). 44](#_Toc226626098)

**[Figure S31.](#_Toc226626099)** [LC–MS/MS spectrum of 2-amino-3-hydroxy-3-(4-methylsulfonylphenyl)propionic acid (TAP-TP-258). 45](#_Toc226626099)

**[Figure S32.](#_Toc226626100)** [LC–MS](#_Toc226626100) **[(A)](#_Toc226626100)** [and LC–MS/MS](#_Toc226626100) **[(B)](#_Toc226626100)** [spectra of 4-methylsulfonylbenzoic acid (TAP-TP-199). 46](#_Toc226626100)

**[Figure S33.](#_Toc226626101)** [LC–MS/MS spectrum of (4-methylsulfonylphenyl)methanol (TAP-TP-185). 47](#_Toc226626101)

**[Figure S34.](#_Toc226626102)** [LC–MS/MS spectrum of 4-methylsulfonylbenzaldehyde (TAP-TP-184). 48](#_Toc226626102)

**[Figure S35.](#_Toc226626103)** [The proposed catabolism pathways of TAP by CS1. The compounds in brackets were not detected and represented the hypothetical intermediates. 49](#_Toc226626103)

**[Figure S36.](#_Toc226626104)** [Transcriptome of CS1 under glucose and CAP treatment. (](#_Toc226626104)**[A](#_Toc226626104)**[) Volcano plots indicating the pairwise comparison of significantly expressed genes of CS1 between CAP and glucose treatments. (](#_Toc226626104)**[B](#_Toc226626104)**[) The log](#_Toc226626104)_[2](#_Toc226626104)_[(Fold change) of the CAP-degrading cluster genes in CAP treatment compared to glucose treatment. The expression fold change was determined based on the normalized counts between the indicated carbon substrates. Log](#_Toc226626104)_[2](#_Toc226626104)_[(Fold change) > 1 or < −1 (](#_Toc226626104)*[p](#_Toc226626104)* [< 0.05) indicated genes significantly up-regulated or down-regulated, respectively. Data are means of three individual experiments ± one standard deviation. 51](#_Toc226626104)

**[Figure S37.](#_Toc226626105)** [Mineralization of](#_Toc226626105) ^[14](#_Toc226626105)^[C-CAP in the Suzhou (SZ) soil at 15°C, 30°C, and 40°C. 52](#_Toc226626105)

**[Figure S38.](#_Toc226626106)** [Bacterial community composition of the SZ soil (](#_Toc226626106)**[A](#_Toc226626106)**[) and the Yingtan (YT) soil (](#_Toc226626106)**[B](#_Toc226626106)**[) at 0, 7, 31, and 37 days. S and Y represent SZ and YT soils. A, C, and P represent CAP pollution with CS1 augmentation, no pollution, and CAP pollution treatments, respectively. 53](#_Toc226626106)

**[Figure S39.](#_Toc226626107)** [OTUs that caused a mean decrease in Gini index greater than 0.5 in random forests analysis in the bacterial communities of SZ (](#_Toc226626107)**[A](#_Toc226626107)**[) and YT (](#_Toc226626107)**[B](#_Toc226626107)**[) soils. 54](#_Toc226626107)

**[Figure S40.](#_Toc226626108)** [Relative abundance of important OTUs (mean decrease in Gini index > 0.5) in the soil bacterial communities of SZ (](#_Toc226626108)**[A](#_Toc226626108)**[) and YT (](#_Toc226626108)**[B](#_Toc226626108)**[) soils during the incubation period. 55](#_Toc226626108)

**[Figure S41.](#_Toc226626109)** [Relative abundance of important OTUs (mean decrease in Gini index > 0.5 in random forest analysis) after normalization using Z-scores in the soil bacterial communities of SZ (A) and YT (B) during the incubation. SC: SZ soil without CAP pollution; SP: SZ soil with CAP pollution; SA: SZ soil with CAP pollution and CS1 augmentation; YC: YT soil without CAP pollution; YP: YT soil with CAP pollution; YA: YT soil with CAP pollution and CS1 augmentation. 56](#_Toc226626109)

**[Figure S42.](#_Toc226626110)** [Shannon index of bacterial communities in SZ (](#_Toc226626110)**[A](#_Toc226626110)**[) and YT (](#_Toc226626110)**[B](#_Toc226626110)**[) soils. Blank: the pristine soil without CAP contamination. Control: CAP-polluted soil in the absence of CS1. CS1: CAP-polluted soil with CS1 bioaugmentation. 57](#_Toc226626110)

**[Figure S43.](#_Toc226626111)** [Phylogenetic tree of the strains carrying](#_Toc226626111) *[chd](#_Toc226626111)* [cluster. The accession numbers were shown in Table S6. The functions of gene products:](#_Toc226626111) **[ChdA](#_Toc226626111)** [(aldehyde dehydrogenase family protein);](#_Toc226626111) **[ChdB](#_Toc226626111)** [(GMC family oxidoreductase);](#_Toc226626111) **[ChdC](#_Toc226626111)** [(L-threonine aldolase);](#_Toc226626111) **[ChdD](#_Toc226626111)** [(hippurate hydrolase);](#_Toc226626111) **[ChdE](#_Toc226626111)** [(Proton-dependent oligopeptide transporter);](#_Toc226626111) **[ChdR](#_Toc226626111)** [(IclR-family transcriptional regulator);](#_Toc226626111) **[GcvA](#_Toc226626111)** [(glycine cleavage system transcriptional activator);](#_Toc226626111) **[GcvH](#_Toc226626111)** [(glycine cleavage system H protein);](#_Toc226626111) **[GcvP](#_Toc226626111)**[: (glycine cleavage system P protein);](#_Toc226626111) **[GcvT](#_Toc226626111)** [(glycine cleavage system T protein);](#_Toc226626111) **[SdaA](#_Toc226626111)** [(L-serine ammonia-lyase); ORF (unannotated open reading frame);](#_Toc226626111) **[PnbA](#_Toc226626111)** [(4-nitrobenzoate reductase);](#_Toc226626111) **[PnbB](#_Toc226626111)** [(4-hydroxyaminobenzoate lyase);](#_Toc226626111) **[PnbX](#_Toc226626111)** [(4-nitrobenzoate-responsive repressor). 59](#_Toc226626111)

**[Figure S44.](#_Toc226626112)** [Antibiotic susceptibility of strain CS1 determined by Kirby–Bauer disk diffusion assay using trimethoprim–sulfamethoxazole. 60](#_Toc226626112)

[TABLES 61](#_Toc226626113)

**[Table S1.](#_Toc226626114)** [Overview of reported chloramphenicol (CAP) degrading bacterial isolates. Their genome accession numbers are provided in Table S2. 61](#_Toc226626114)

**[Table S2.](#_Toc226626115)** [The physiochemical properties of Suzhou (SZ) and Yingtan (YT) soils. 63](#_Toc226626115)

**[Table S3.](#_Toc226626116)** [Strains carrying](#_Toc226626116) *[chd](#_Toc226626116)* [cluster, their isolation sources, and accession numbers. 64](#_Toc226626116)

**[Table S4.](#_Toc226626117)** [Genes of protocatechuic acid downstream metabolism in strains CS1. 65](#_Toc226626117)

**[Table S5.](#_Toc226626118)** [Genes carried by CS1 against environmental stress. 66](#_Toc226626118)

**[Table S6.](#_Toc226626119)** [CAP-degrading strains carrying genes](#_Toc226626119) *[chdABCD](#_Toc226626119)*[, and their genome accession numbers. 67](#_Toc226626119)

**[Table S7.](#_Toc226626120)** [Genes in the CS1 genome encoding protein homologous to Virulence Factor Database (VFDB). 68](#_Toc226626120)

**[Table S8.](#_Toc226626121)** [Genes in the CS1 genome with >70% protein sequence identity of matching region to reference sequences in VFDB. 70](#_Toc226626121)

[REFERENCES 71](#_Toc226626122)

# TEXTS

**Text S1.** The measurements of soil properties

The measurement methods of pH, soil organic carbon (SOM), cation exchange capacity (CEC) were consistent with our previous study.[[1](#_ENREF_1)] The soil pH was recorded in a soil/water suspension (1:2.5, w/v).[[2](#_ENREF_2)] The content of SOM was determined by the dichromate redox colorimetric method.[[2](#_ENREF_2)] Briefly, 0.5 g soil was digested with 0.4 mol/L potassium dichromate sulfuric acid solution of 10 mL under 180 ºC for 5 min, then the excess potassium dichromate was titrated with ferrous sulfate standard solution until the color changed from orange to green, and ended up with reddish brown. The SOM was calculated from the amount of potassium dichromate consumed using the oxidation correction coefficient. The effective CEC was determined by the barium chloride buffer method.[[3](#_ENREF_3)] For each measurement, 0.5 g soil in 30 mL BaCl_2_ (0.1 mol/L) solution was shaken for 2 hours, then was centrifugated and filtrated. The filtrate was analyzed by inductively coupled plasma optical emission spectrometer (ICP-OES, PQ9000, Analytik, Jena, Germany) to determine the amounts of the following cations: Ca, Mg, K, Na, Al, Fe, and Mn. The CEC value was calculated according to the total amount of these cations. The total nitrogen (TN) was determined using the Kjeldahl digestion procedure.[[2](#_ENREF_2)]

**Text S2.** The mineral salt media components

The mineral salt medium (MSM with pH of 7.0–7.2) includes 2.44 g/L of Na_2_HPO_4_, 1.52 g/L of KH_2_PO_4_, 0.5 g/L of (NH_4_)_2_SO_4_, 0.2 g/L of MgSO_4_, 0.05 g/L of CaCl_2_⋅2H_2_O, 5.0 mg/L of EDTA, 2.0 mg/L of FeSO_4_⋅7H_2_O, 0.9 mg/L of ZnSO_4_⋅7H_2_O, 0.27 mg/L of MnCl_2_⋅4H_2_O, 2.7 mg/L of H_3_BO_3_, 1.8 mg/L of CoCl_2_⋅6H_2_O, 0.09 mg/L of CuCl_2_⋅2H_2_O, 0.18 mg/L of NiCl_2_⋅6H_2_O, and 0.27 mg/L of Na_2_MoO_4_⋅2H_2_O.

**Text S3.** Genomic assembly

For genome assembly, the filtered subreads were assembled by Hifiasm.[[4](#_ENREF_4)] Circulator v1.5.5[[5](#_ENREF_5)] was applied to cyclize the assembled draft genome. Genome assembly improvement was performed by Pilon v1.22,[[6](#_ENREF_6)] utilizing NovaSeq PE150 sequencing data.

**Text S4.** Transcriptome data filtering

Sequence quality control and data filtering were performed with fastp (v0.19.7),[[7](#_ENREF_7)] and rRNA reads were filtered out before analysis. Hisat 2 (v2.1.0) was used to map clean reads to the reference genome.[[8](#_ENREF_8)]

**Text S5.** Homologous genes cluster analysis

The GenBank and RefSeq protein files of bacteria in Class Actinomycetes (including genomes and metagenome assembled genome) were downloaded from the NCBI database (<https://www.ncbi.nlm.nih.gov/datasets/>). Subsequently, the protein sequences were aligned with *chdABCD* genes of strain CS1 using BLAST (v2.14.0) with the criteria (>40% identity, >70% query length coverage, and <1e-5 e-value). The genomes carried *chdABCD* were considered for gene arrangement of CAP-degrading gene clusters visualized using the R package gggenes (v0.5.1) (<https://github.com/wilkox/gggenes>).

**Text S6.** Quantification using high-performance liquid chromatography (HPLC) and metabolite identification using HPLC coupled with high-resolution mass spectrometer

Quantification of chloramphenicol (CAP), thiamphenicol (TAP), florfenicol (FF), 2-nitrobenzoic acid (2NBA), 3-nitrobenzoic acid (3NBA), 4-nitrocinnamic acid (4NCN), and 4-nitrobenzoic acid methyl ester (4NME) was conducted using a HPLC (e2695, Waters Co., Milford, MA) system equipped with a C18 column (Symmetry, 5 μm, 4.6 mm × 250 mm, Waters Co., Milford, MA). The mobile phase, consisting of acetonitrile (phase A) and water with 0.1% formic acid (phase B), was at a flow rate of 1.0 mL/min in isocratic mode for CAP, 2NBA, 3NBA, 4NCN, and 4NME (35% A and 65% B), or TAP and FF (20% A and 80% B). UV detector (e2487, Waters Co., Milford, MA) was set at 270 and 215 nm for the detection of CAP, TAP and FF.

Metabolites of CAP and TAP were identified using an Agilent 1260 infinity HPLC system in tandem with a quadrupole time-of-flight mass spectrometer (TripleTOF 5600, AB Sciex LLC, Framingham, MA). Chromatographic separation was conducted on a C18 column (X-Bridge, 3.5 μm, 2.1 mm × 100 mm) at 20 °C. The mobile phase consisted of 0.1% formic acid (gradient grade for LC; Anaqua™ Chemicals, Wilmington, DE) in water (Phase A) and methanol (Phase B; gradient grade for LC; Merck, Darmstadt, Germany), and the flow rate was 200 μL/min in isocratic mode (80% A and 20%B). The mass spectrometer was operated with electrospray ionization (ESI) source in negative ion mode to record mass spectra (m/z 30‒500) under the following conditions: ionspray voltage floating, -4500 V; temperature, 550 °C; ion source gas 1, 55 psi; ion source gas 2, 55 psi; curtain gas, 35 psi; declustering potential, -80 V; collision energy, -10 V. Following the MS analysis, the precursor ions of possible transformation products were individually selected for MS/MS analysis, and the fragmentation was optimized by varying collision energy from 25 to 45 eV. During the analysis, nitrogen gas was used throughout, and the accuracy of the MS and MS/MS data was ensured by a calibration error of < 3 ppm during external mass calibration with APCI negative calibration solution. The mass spectrometric data were acquired with Analyst TF software (Version 1.6, AB Sciex LLC, Framingham, MA) and processed using PeakView software (Version 1.2, AB Sciex LLC, Framingham, Massachusetts, USA).

Structural annotation of CAP transformation intermediates was supported by characteristic isotope patterns and diagnostic MS/MS fragments. For dichlorinated compounds, the chlorine isotope pattern (M, M+2, and M+4 peaks with an approximate intensity ratio of 9:6:1) was used as supporting evidence for molecular formula assignment. For nitroaromatic-related intermediates, MS/MS spectra showed diagnostic fragment ions consistent with sequential nitro-group reduction products, including fragments characteristic of nitrobenzene-, nitrosobenzene-, phenylhydroxylamine-, and aniline-type structures.

**Text S7.** The 16S rRNA gene amplicon sequence analysis

DNA extraction using FastDNA Spin Kit for Soil DNA Extraction (MP Biomedicals; Irvine, CA). The 16S rRNA genes were amplified using 338F and 806R primers and sequencing at Guangdong Magigene Biotechnology Co., Ltd. (Guangzhou, China). Raw 16S rRNA gene sequencing data was process using QIIME2.[[9](#_ENREF_9)] Diversity analysis and random forest modeling were carried out in R (version 4.3.3) using the “vegan” (version 2.6-6.1) and “random Forest” (version 4.7-1.1) packages, respectively.

# FIGURES

**
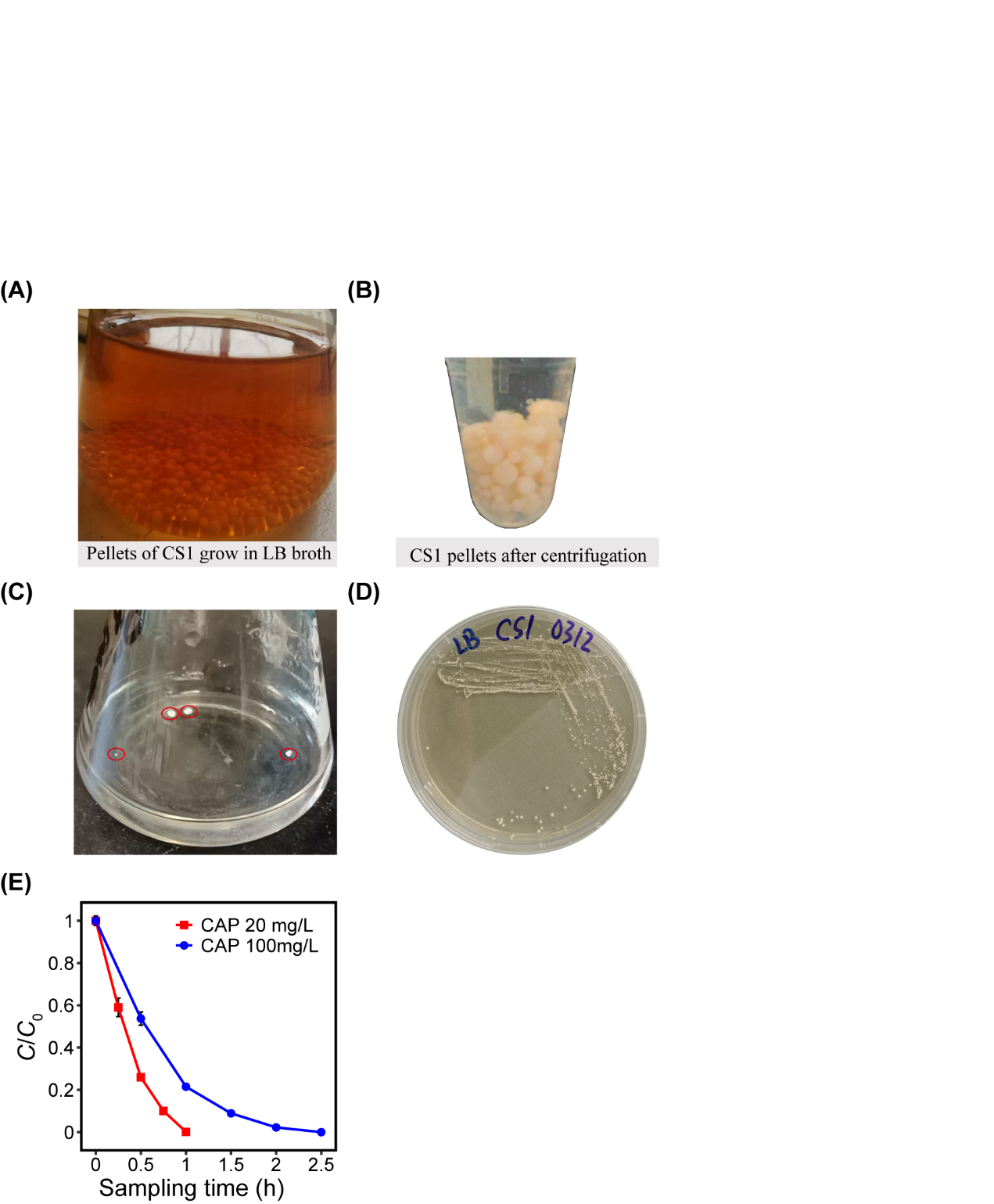
**

**Figure S1.** The morphologies of strain CS1 and its chloramphenicol (CAP) degrading capacity. **(A)** The aggregation biofilm of CS1 cultured in LB broth at 150 rpm, and **(B)** the pellets (1–3 mm) after washing with mineral salt medium. **(C)** Biofilm (marked with red circles) grown at the air-liquid interface after CS soil enrichment acclimated with CAP as the only carbon source. **(D)** Colonies of strain CS1 grown on LB agar plates for 6 days. **(E)** Degradation dynamics of 20 and 100 mg/L CAP by strain CS1 at 30°C and pH 7.


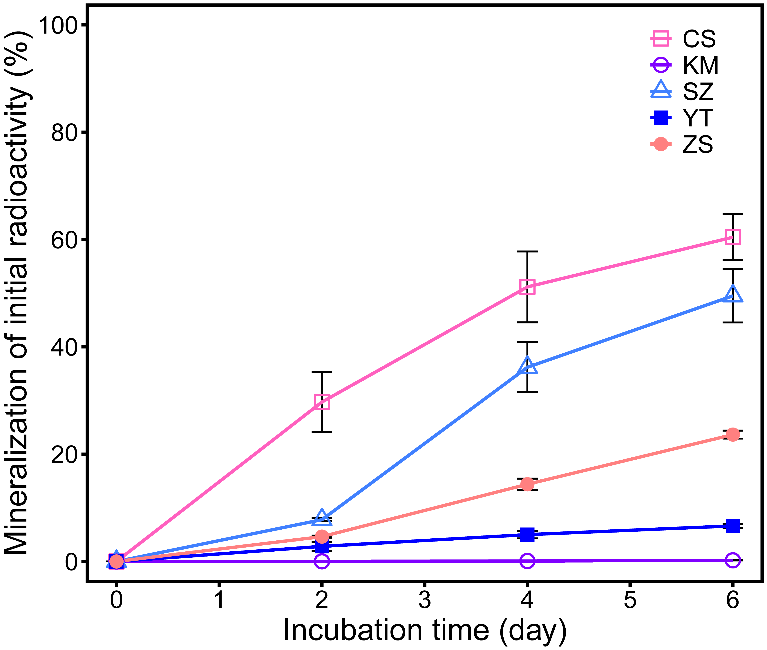


**Figure S2.** Mineralization of ^14^C-Chloramphenicol (^14^C-CAP) in the five farmland soils from Changsha (CS), Kunming (KM), Suzhou (SZ), Yingtan (YT), and Zhoushan (ZS), respectively. The soil incubation experiment was conducted at 30°C in darkness with shaking of 150 rpm.


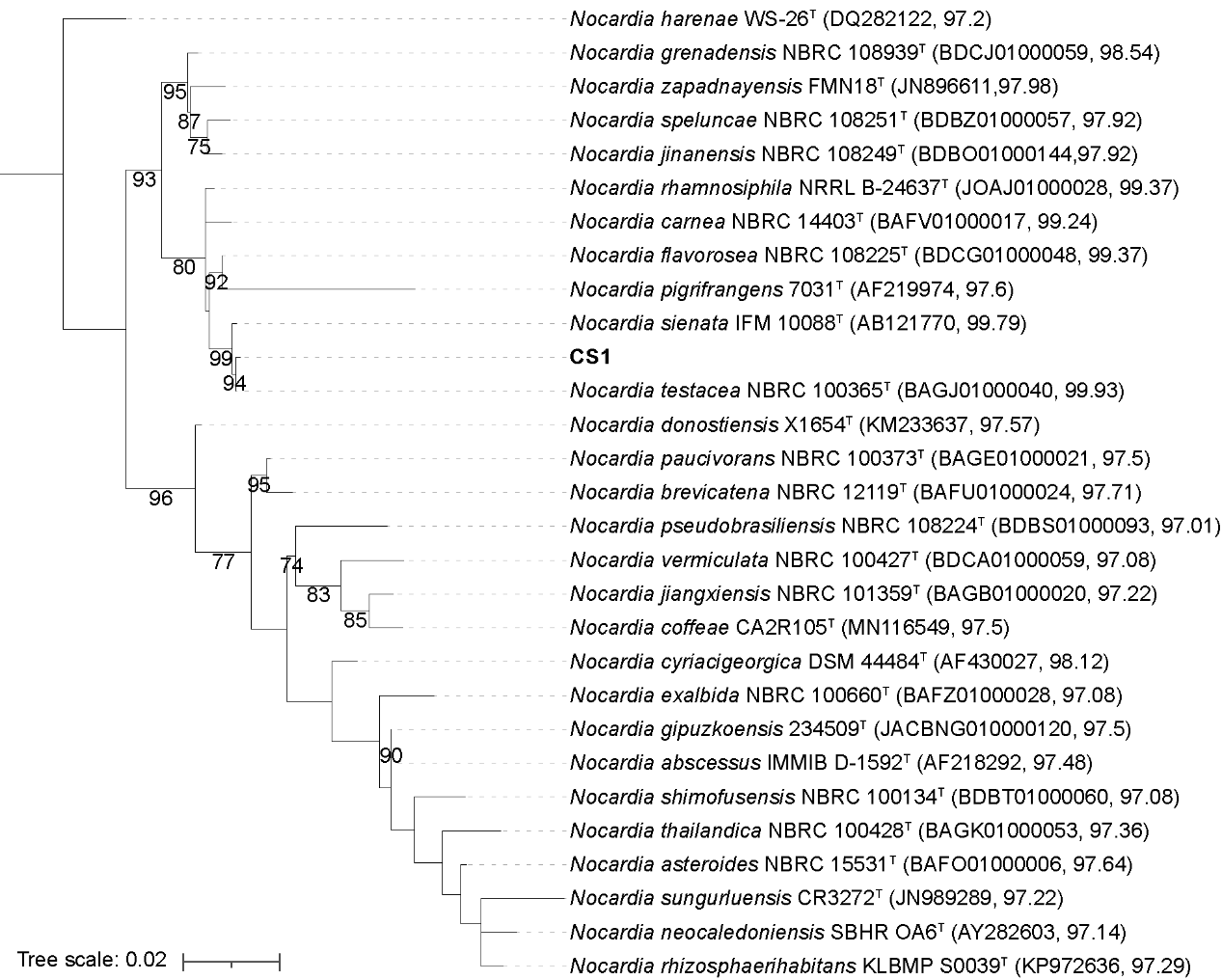


**Figure S3.** Phylogenetic tree of CS1with the species greater than 97% identity of the 16S rRNA gene. Accession numbers and identity with the 16S rRNA gene of CS1 are provided in parentheses.


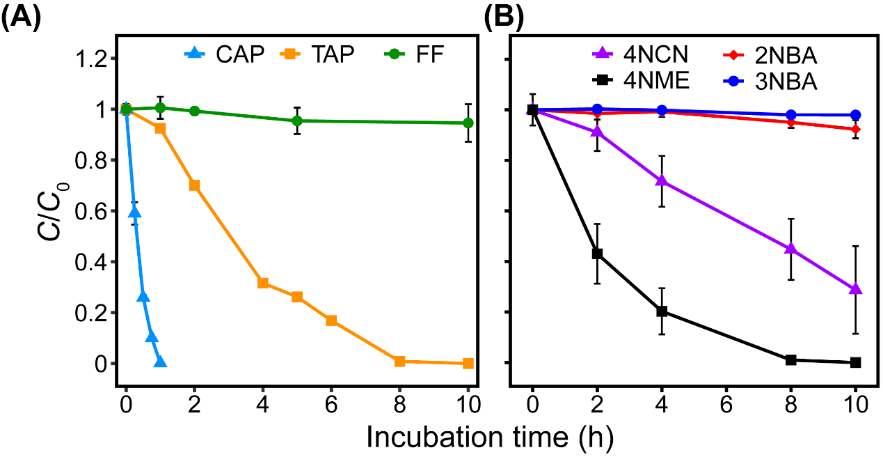


**Figure S4.** Degradation kinetics of CAP structural-like compounds by CS1, including **(A)** amphenicols of chloramphenicol (CAP), thiamphenicol (TAP), and florfenicol (FF); **(B)** nitroaromatic compounds of 2-nitrobenzoic acid (2NBA), 3-nitrobenzoic acid (3NBA), 4-nitrocinnamic acid (4NCN) and 4-nitrobenzoic acid methyl ester (4NME).


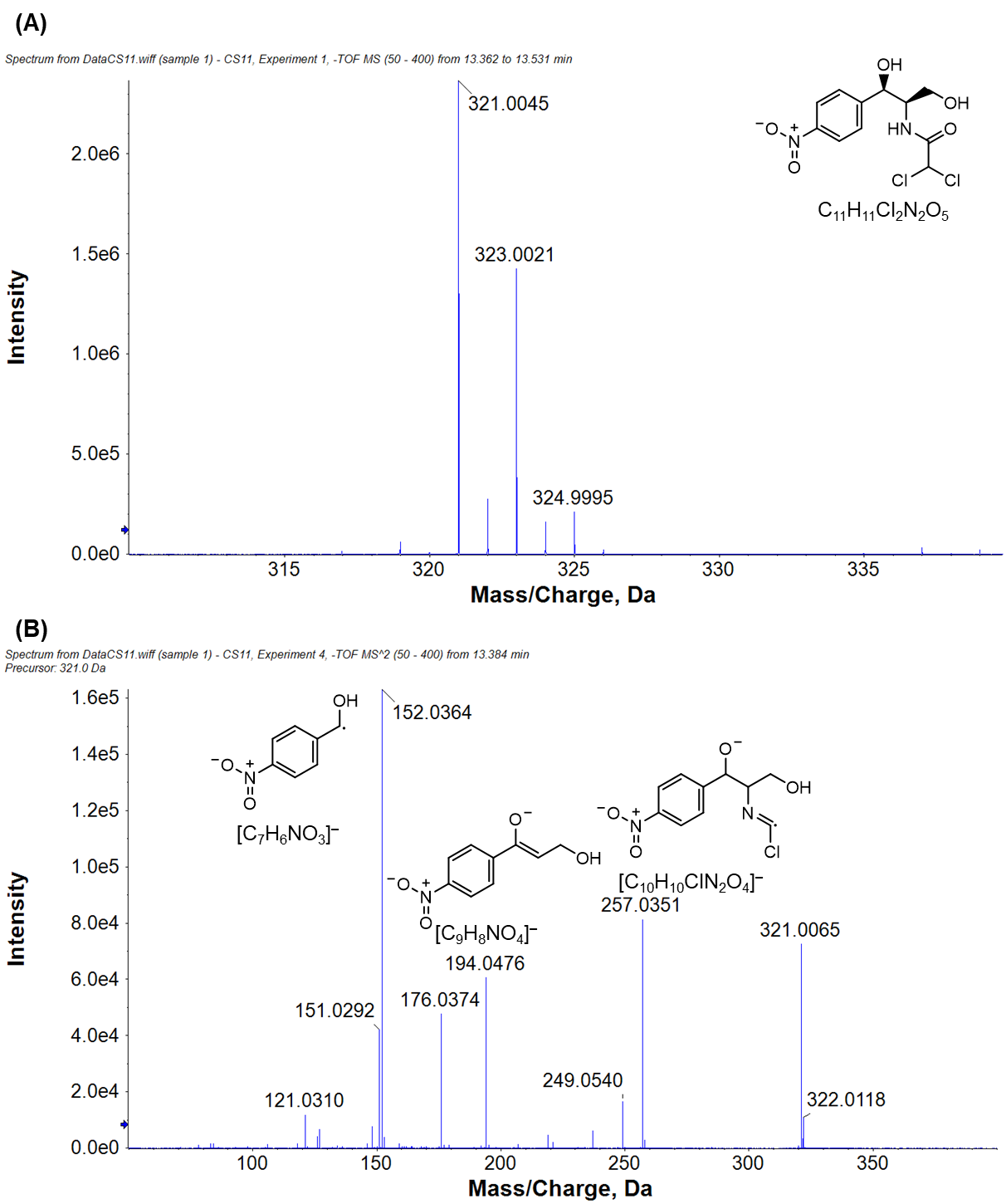


**Figure S5.** LC–MS **(A)** and LC–MS/MS **(B)** spectra of CAP.


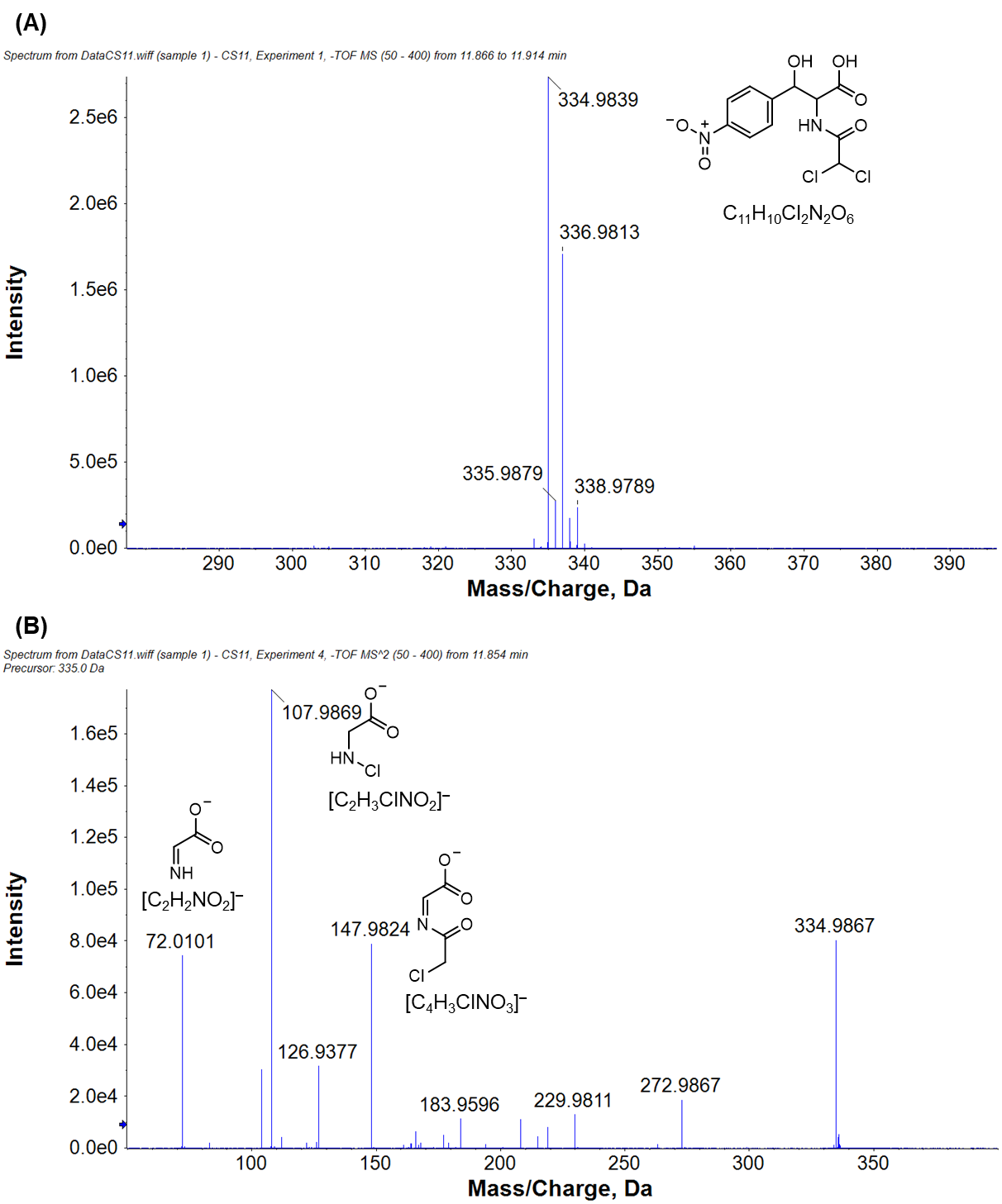


**Figure S6.** LC–MS **(A)** and LC–MS/MS **(B)** spectra of 2-(2,2-dichloroacetamido)-3-hydroxy-3-(4-nitrophenyl)propanoic acid (compound TP-335).


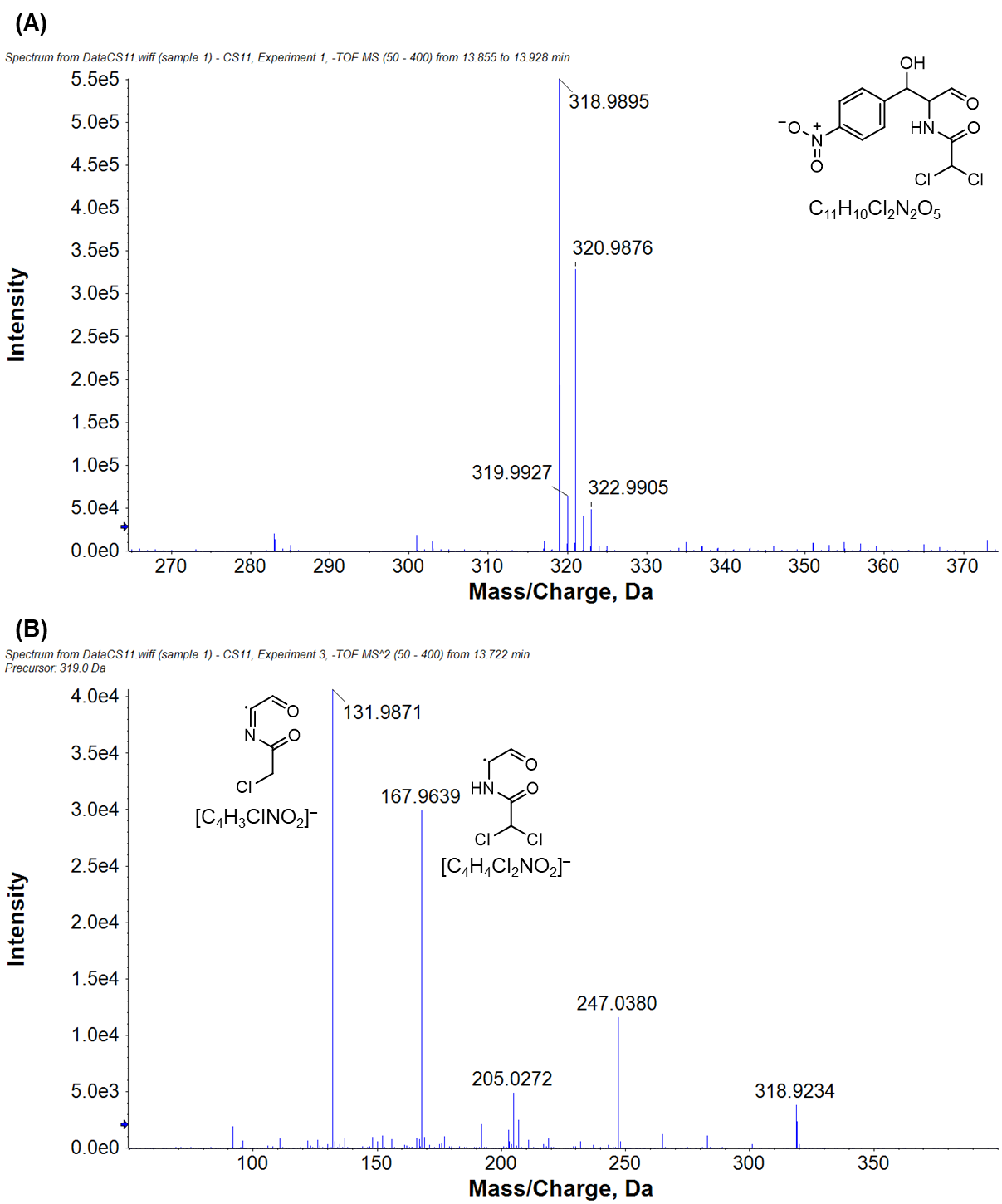


**Figure S7.** LC–MS **(A)** and LC–MS/MS **(B)** spectra of 2,2-dichloro-*N*-[1-hydroxy-1-(4-nitrophenyl)-3-oxo-2-propanyl]acetamide (compound TP-319).


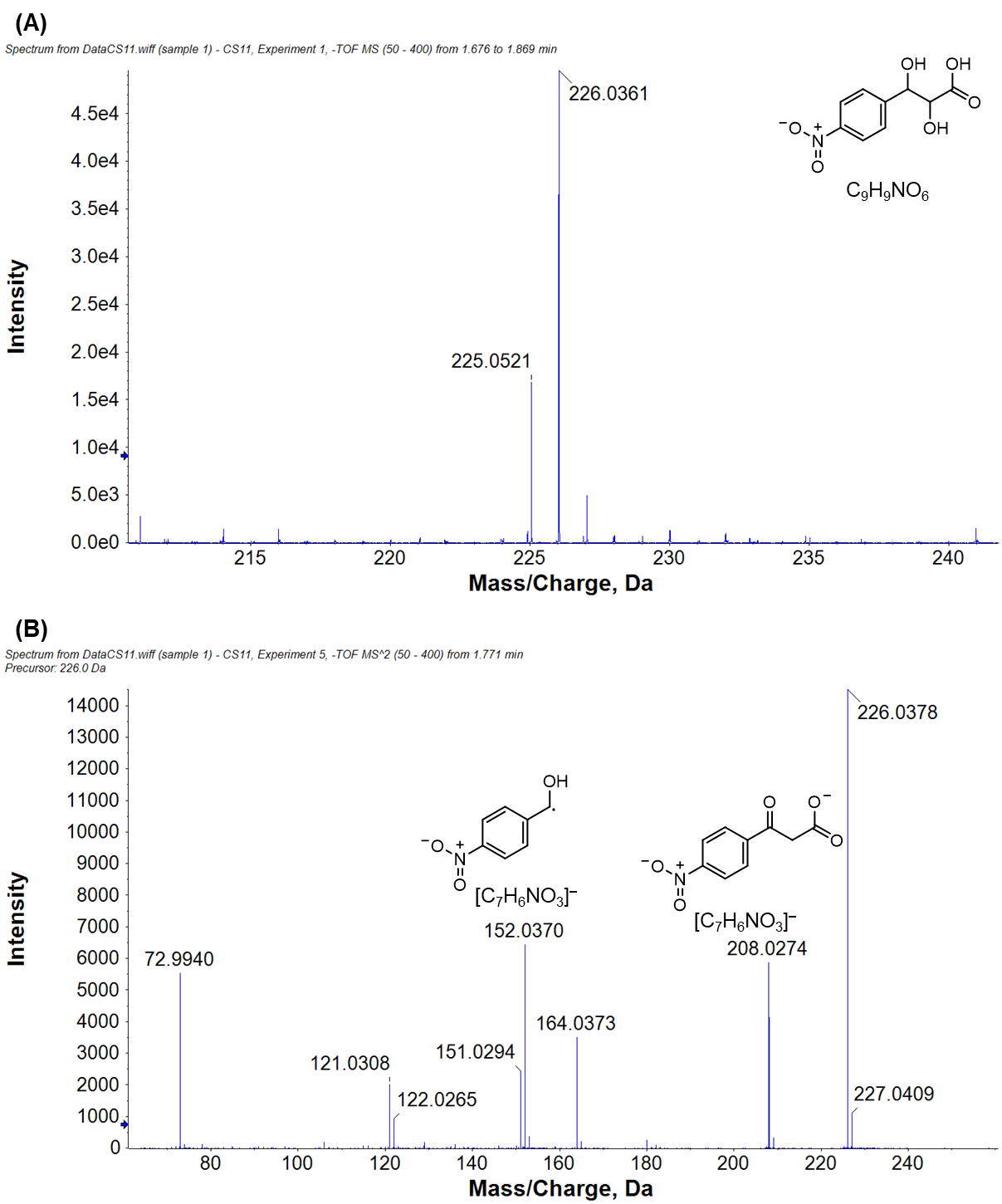


**Figure S8.** LC–MS **(A)** and LC–MS/MS **(B)** spectra of 2,3-dihydroxy-3-(4-nitrophenyl)propanoic acid (compound TP-226).


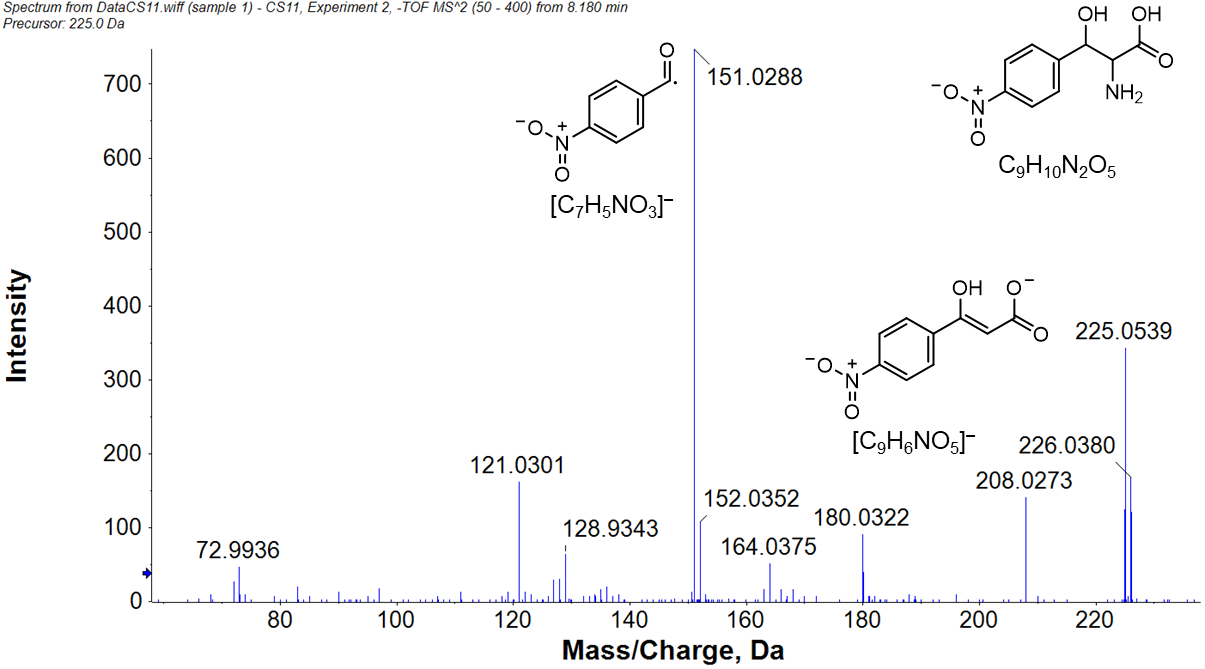


**Figure S9.** LC–MS/MS spectrum of 2-amino-3-hydroxy-3-(4-nitrophenyl)propanoic acid (compound TP-225).


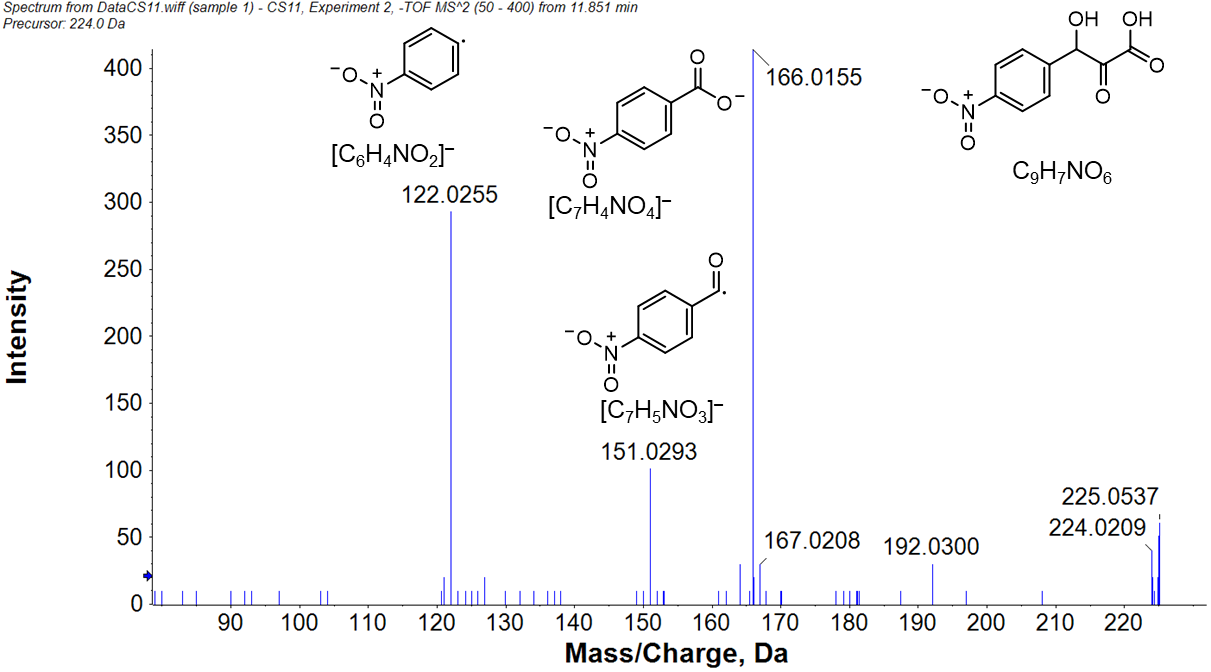


**Figure S10.** LC–MS/MS spectrum of 3-hydroxy-3-(4-nitrophenyl)-2-oxopropanoic acid (compound TP-224).


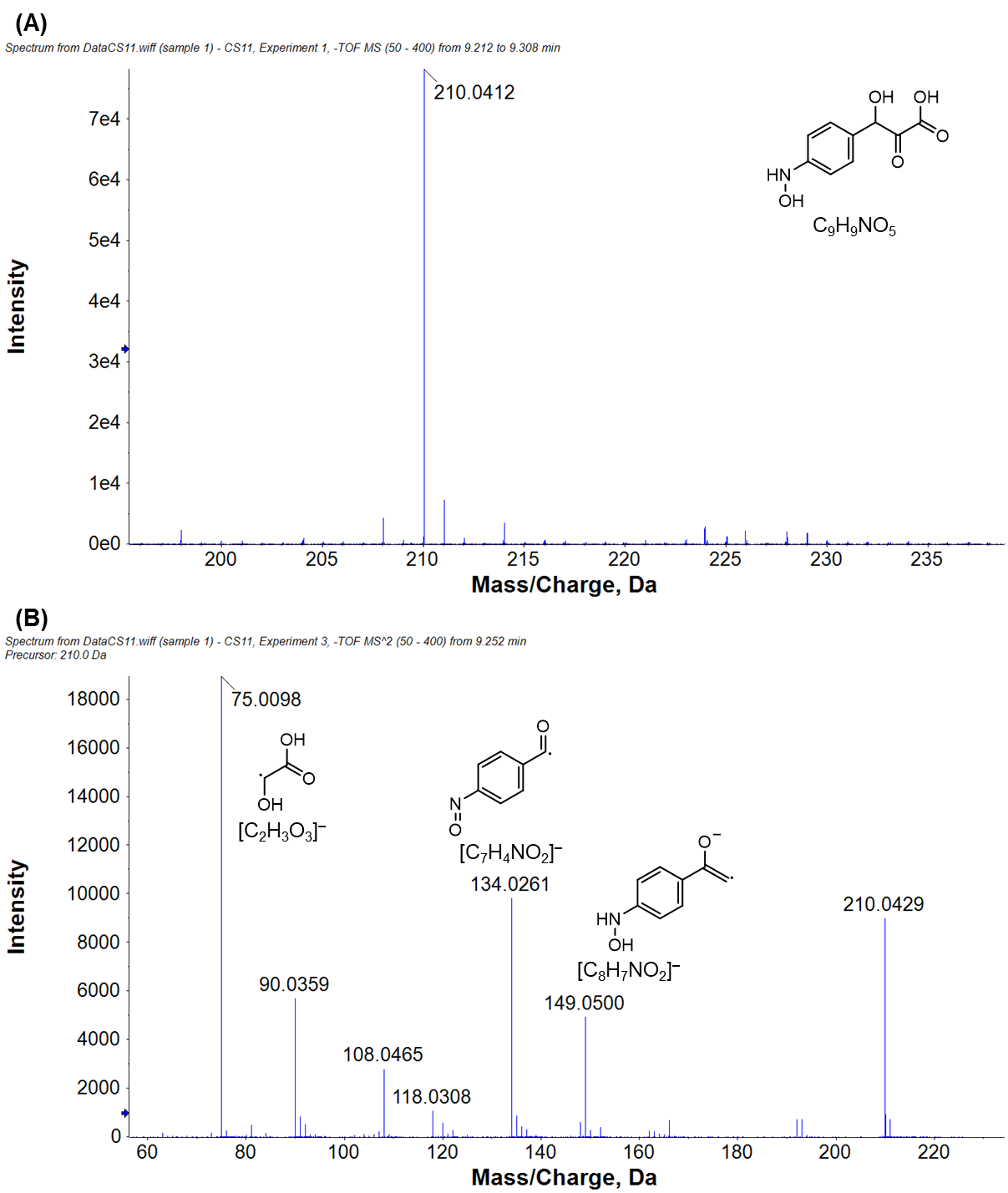


**Figure S11.** LC–MS **(A)** and LC–MS/MS **(B)** spectra of 3-hydroxy-3-(4-hydroxyaminophenyl)-2-oxopropanoic acid (compound TP-210).


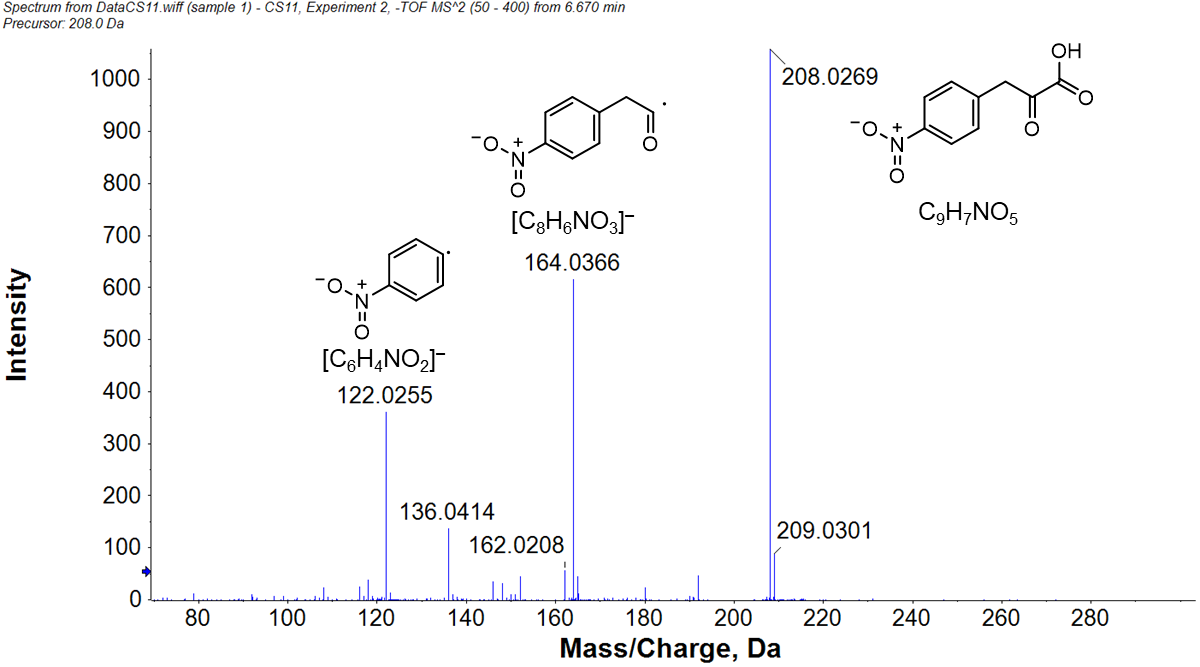


**Figure S12.** LC–MS/MS spectrum of 3-(4-nitrophenyl)-2-oxopropanoic acid (compound TP-208a).


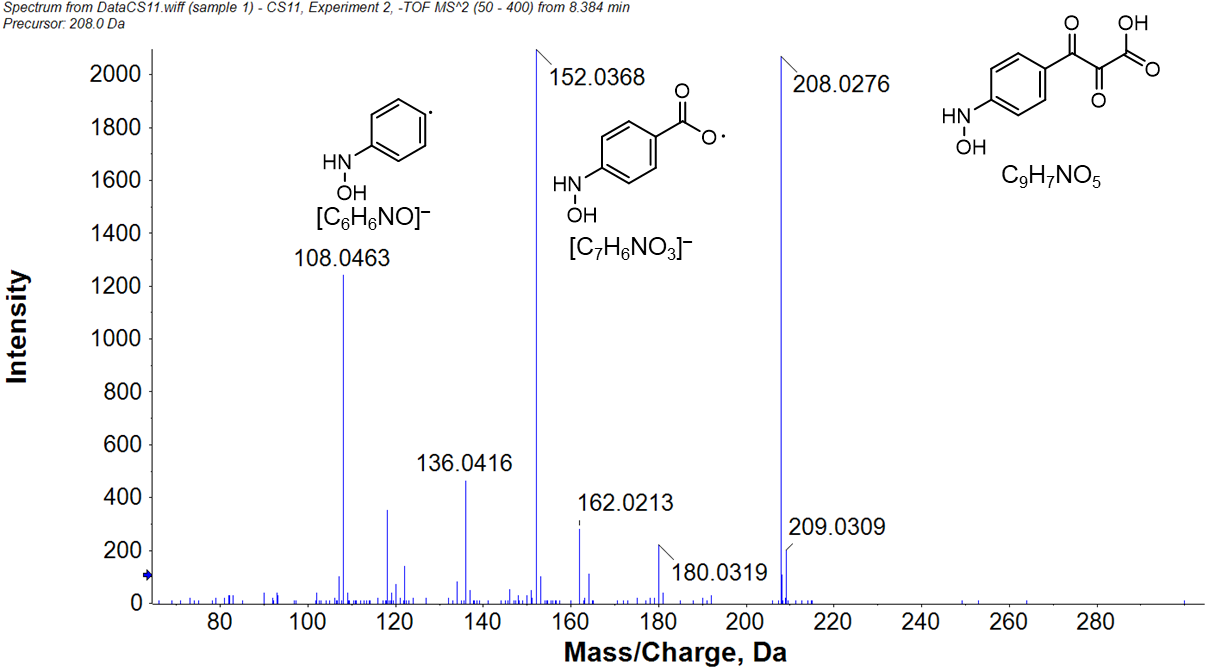


**Figure S13.** LC–MS/MS spectrum of 3-(4-hydroxyaminophenyl)-2,3-dioxopropanoic acid (compound TP-208b).


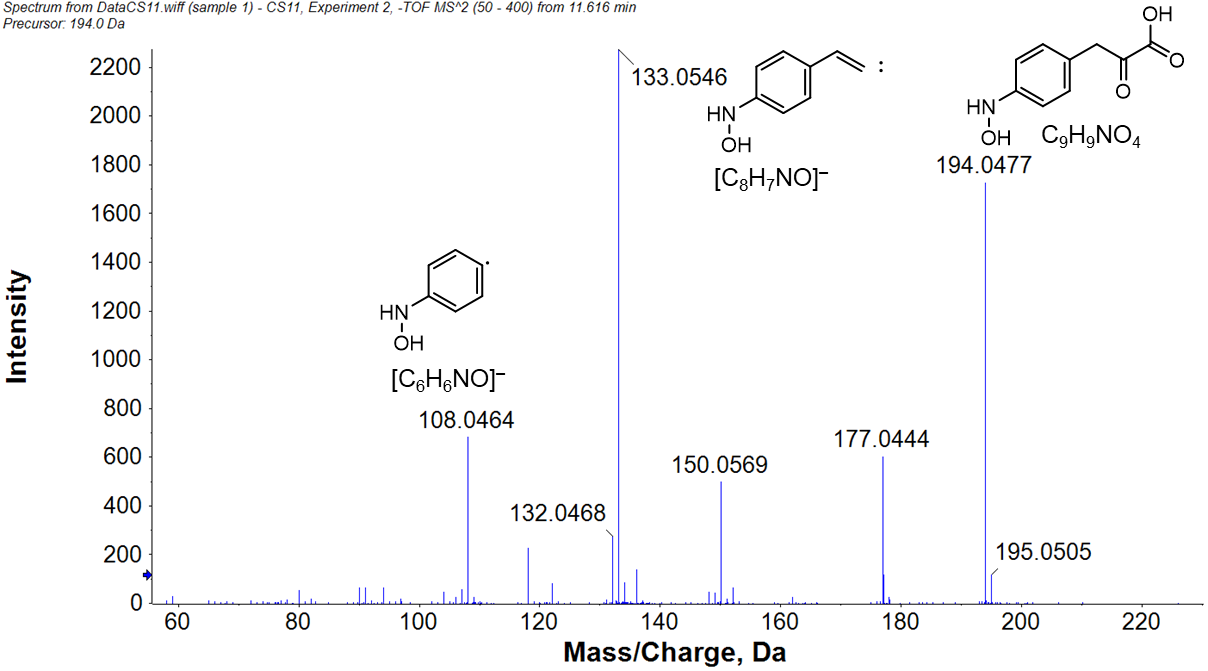


**Figure S14.** LC–MS/MS spectrum of 3-(4-hydroxyaminophenyl)-2-oxopropanoic acid (compound TP-194).


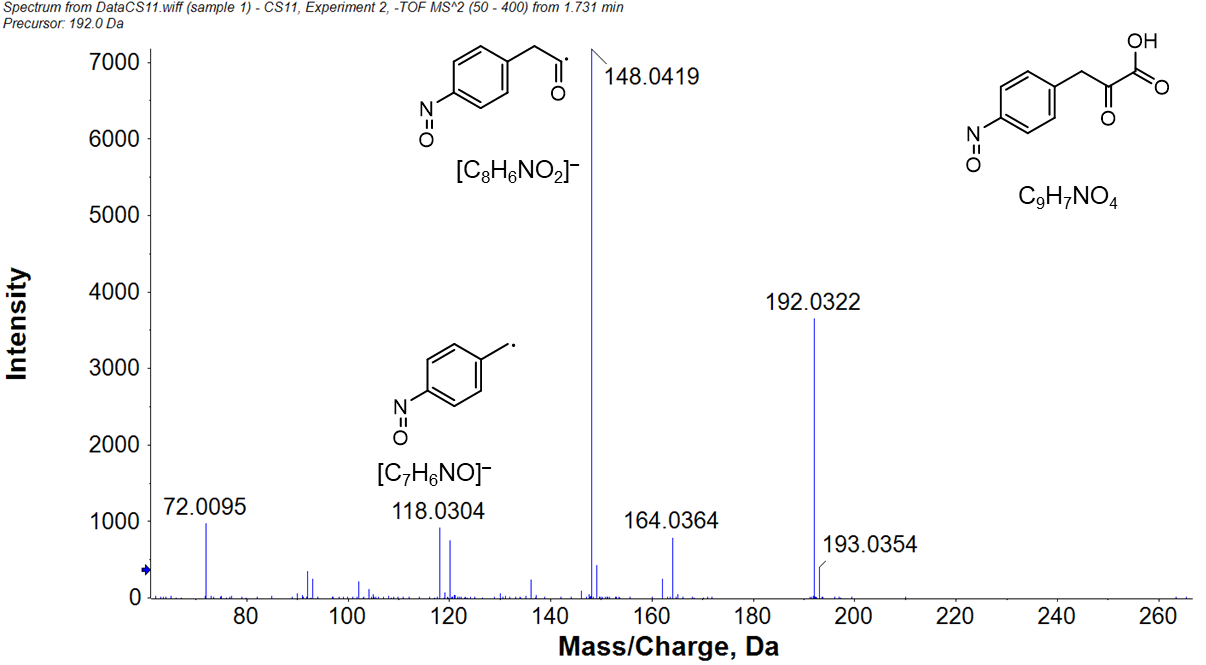


**Figure S15.** LC–MS/MS spectrum of 3-(4-nitrosophenyl)-2-oxopropanoic acid (compound TP-192).


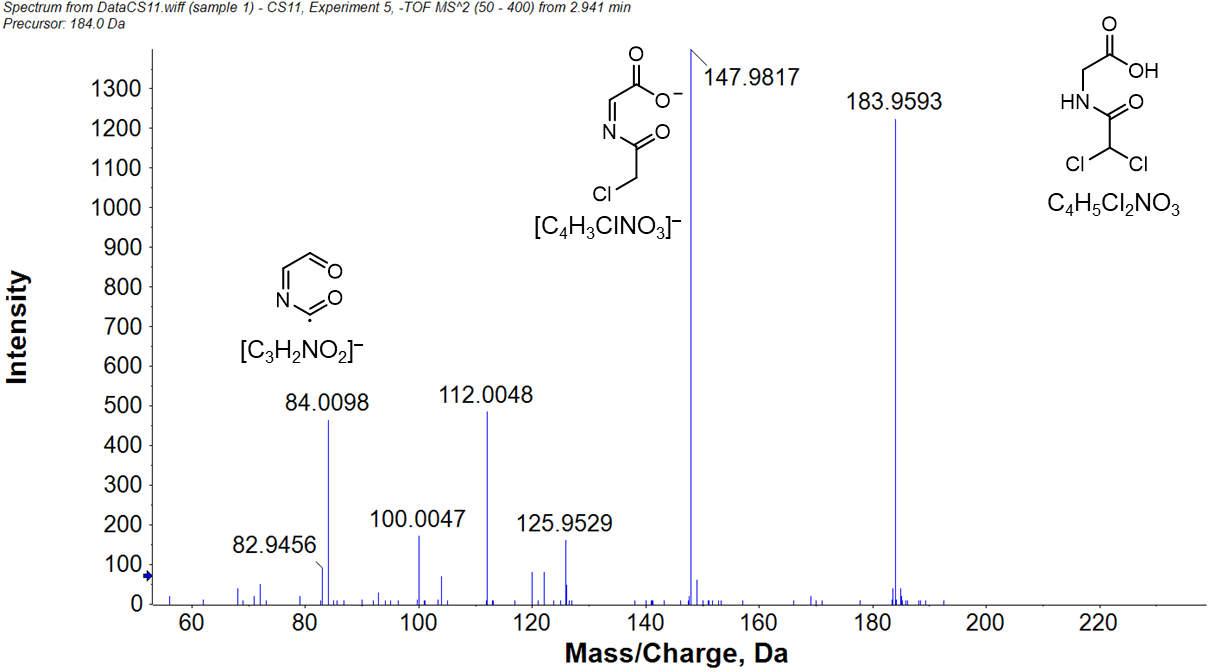


**Figure S16.** LC–MS/MS spectrum of 2-(2,2-dichloroacetamido)acetic acid (compound TP-184).


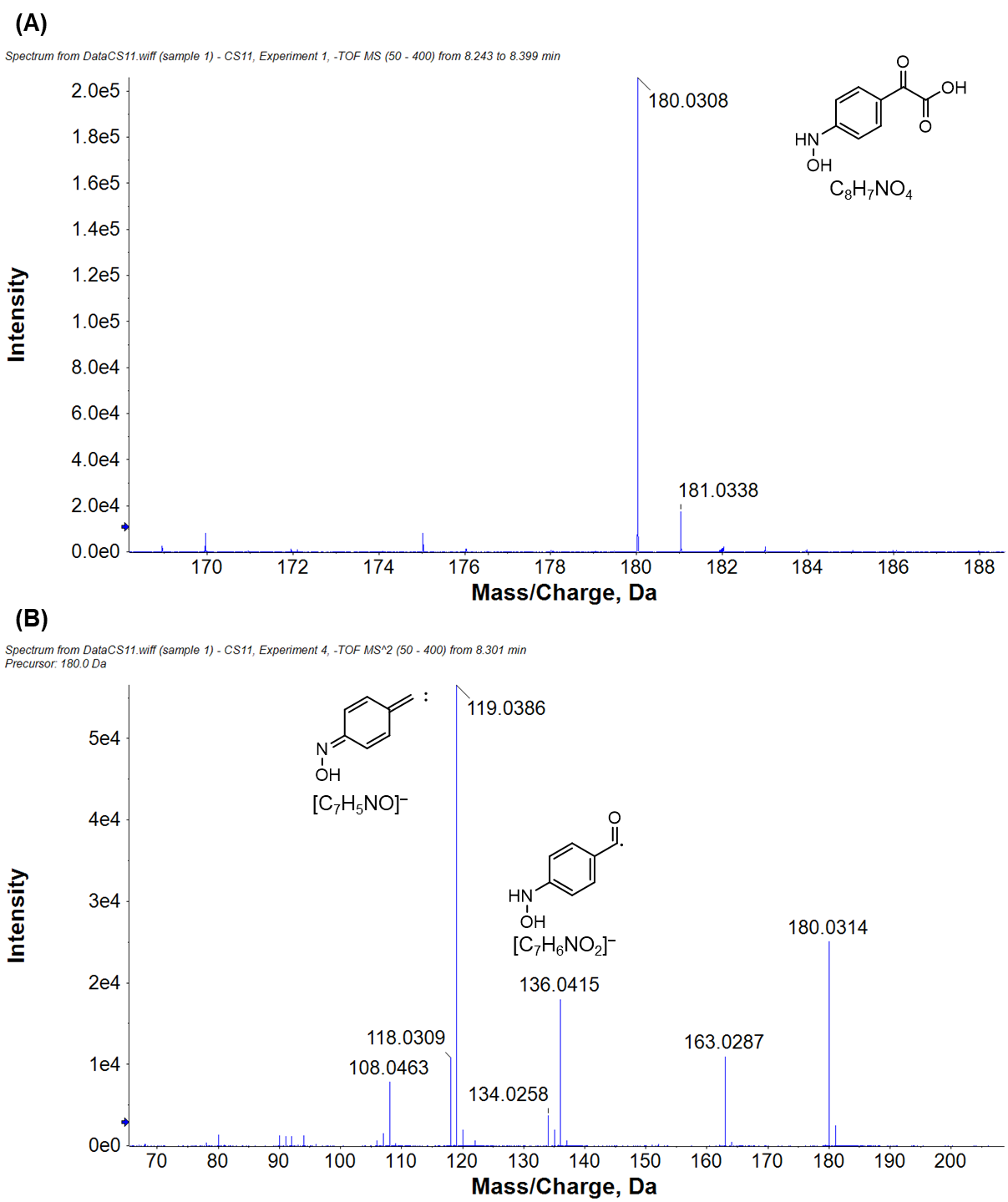


**Figure S17.** LC–MS **(A)** and LC–MS/MS **(B)** spectra 2-(4-hydroxyaminophenyl)-2-oxoacetic acid (TP-180).


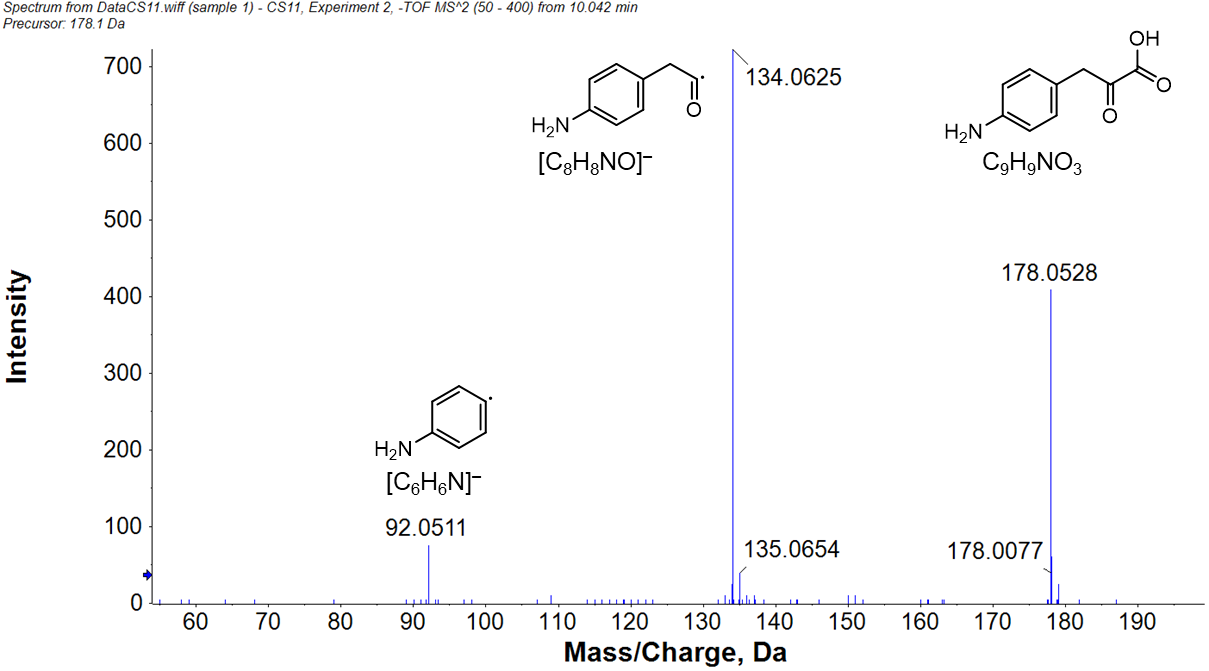


**Figure S18.** LC–MS/MS spectrum of 3-(4-Aminophenyl)-2-oxopropanoic acid (TP-178).


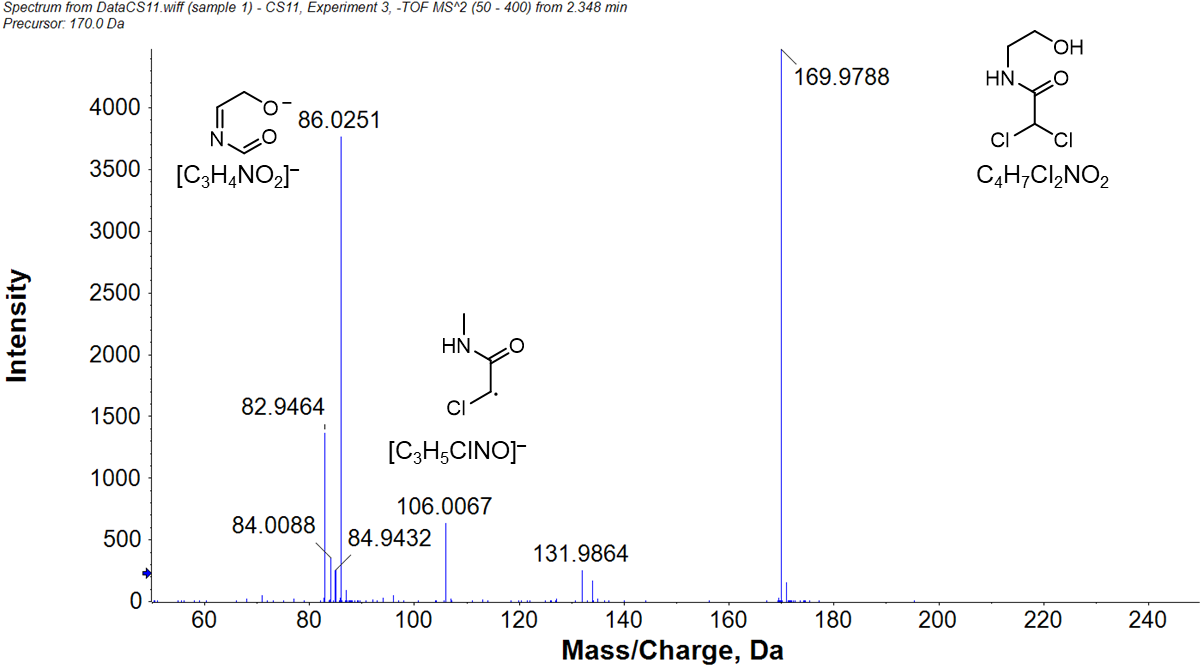


**Figure S19.** LC–MS/MS spectrum of 2,2-dichloro-*N*-(2-hydroxyethyl)acetamide (TP-170).


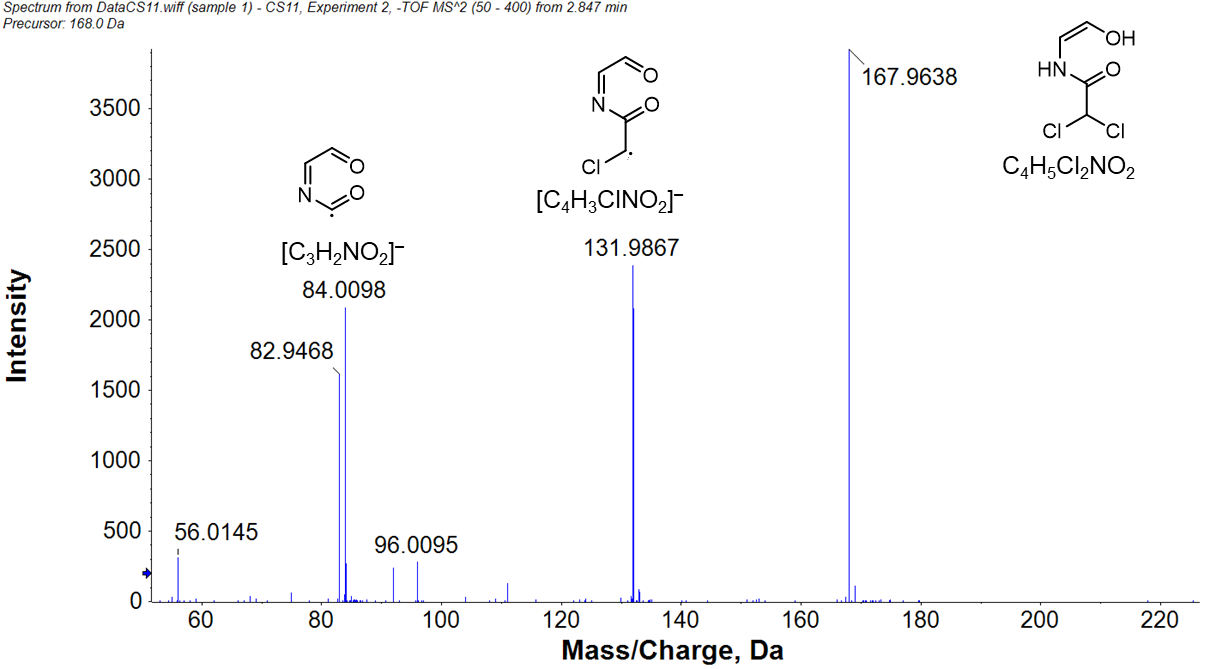


**Figure S20.** LC–MS/MS spectrum of 2,2-dichloro-*N*-(2-oxoethyl)acetamide (TP-168).


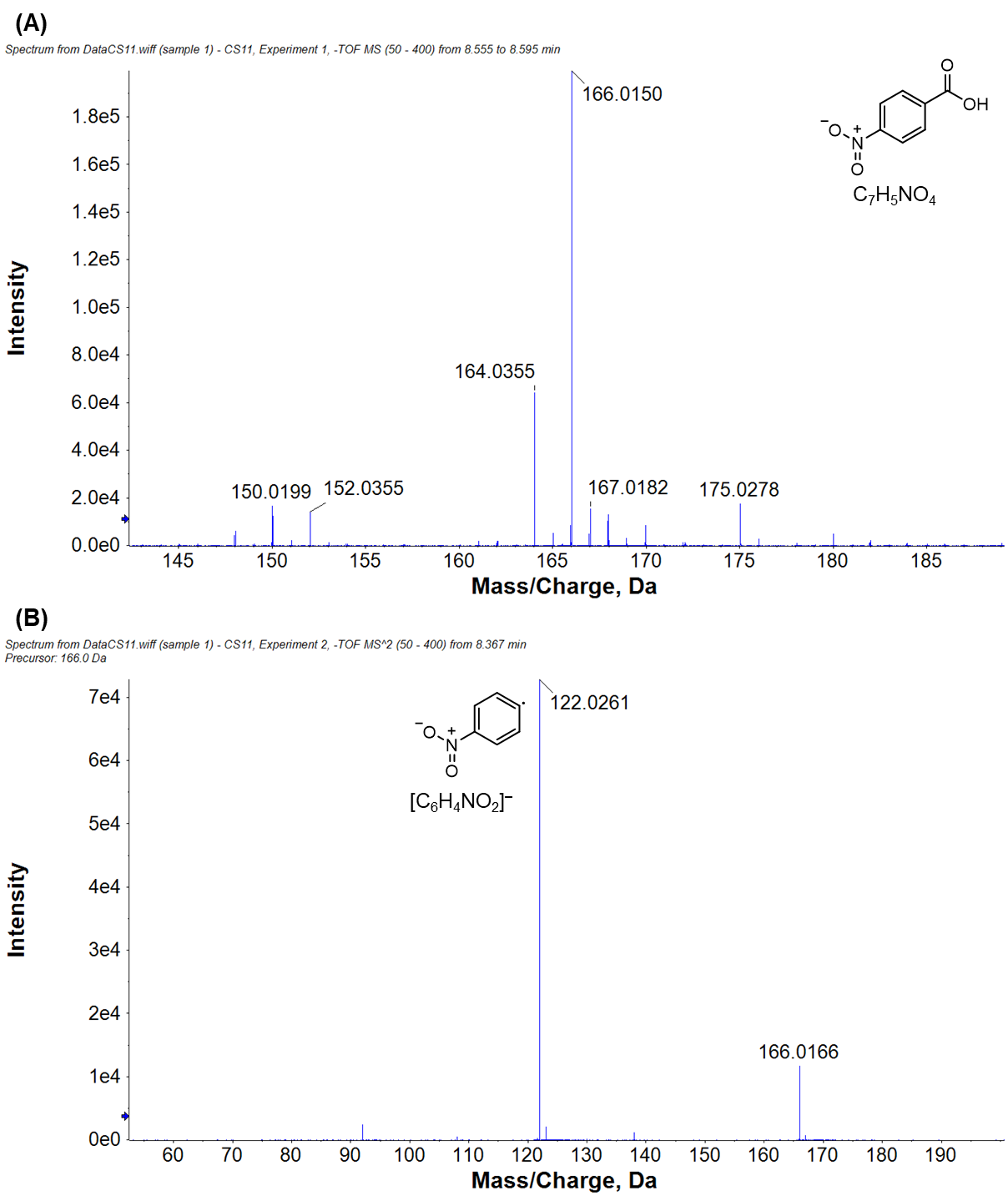


**Figure S21.** LC–MS **(A)** and LC–MS/MS **(B)** spectra of 4-nitrobenzoic acid (TP-166).


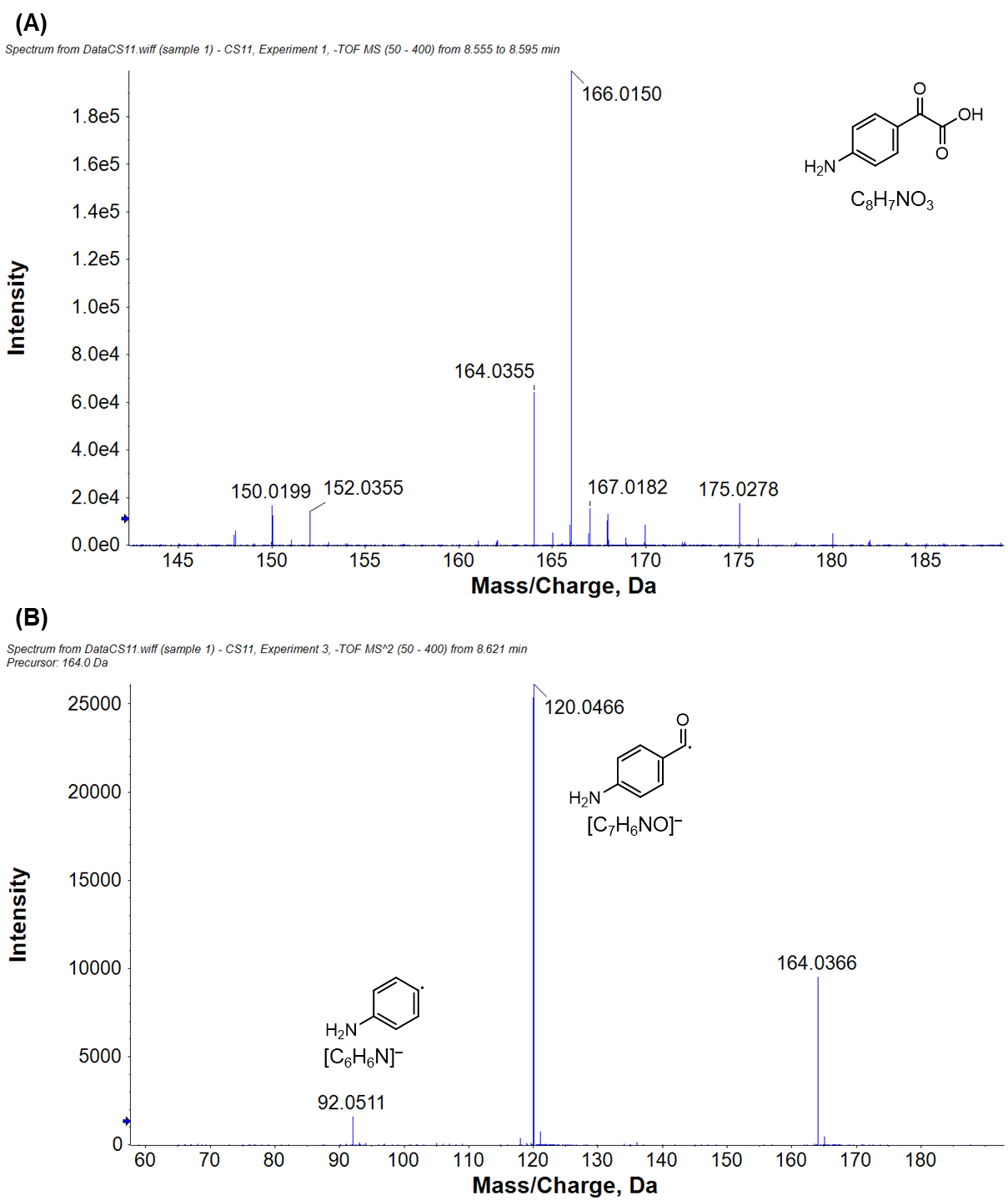


**Figure S22.** LC–MS **(A)** and LC–MS/MS **(B)** spectra of 2-(4-aminophenyl)-2-oxoacetic acid (TP-164).


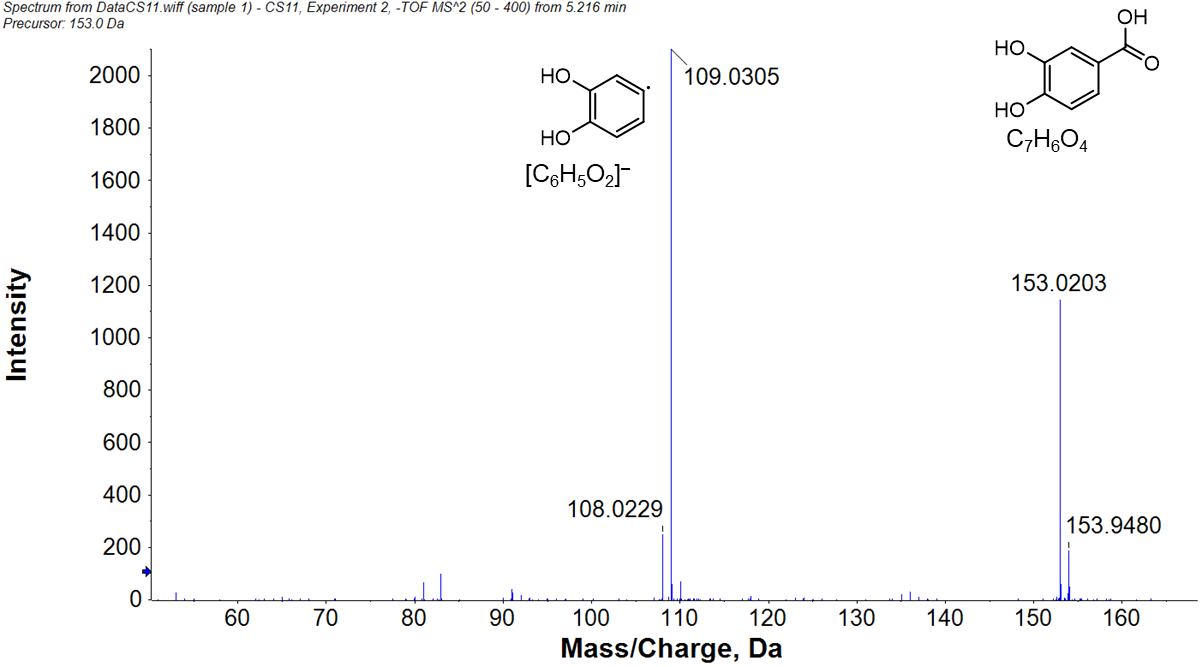


**Figure S23.** LC–MS/MS spectrum of protocatechuic acid (TP-153).


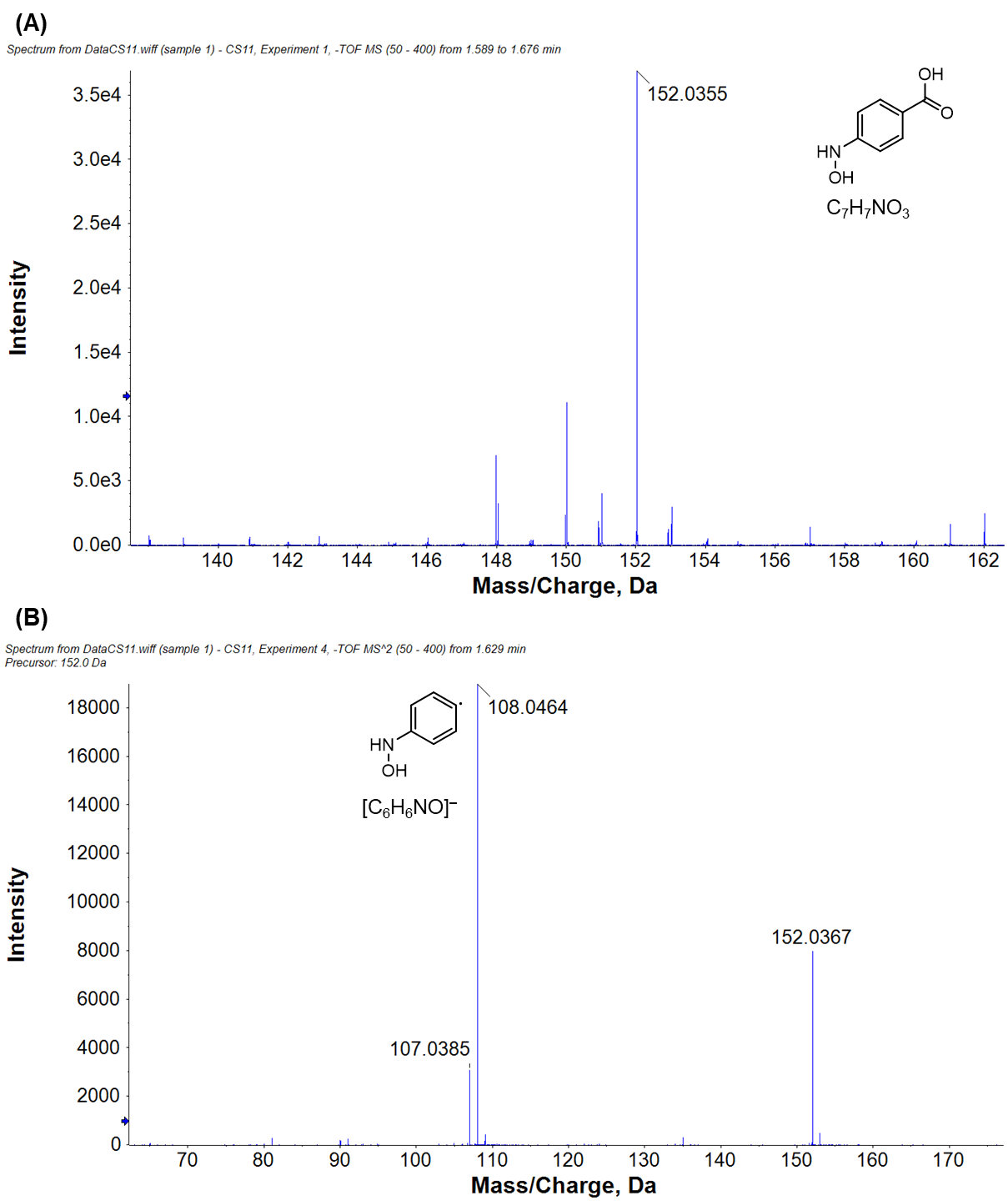


**Figure S24.** LC–MS **(A)** and LC–MS/MS **(B)** spectra of 4-nitrosobenzoic acid (TP-152a).


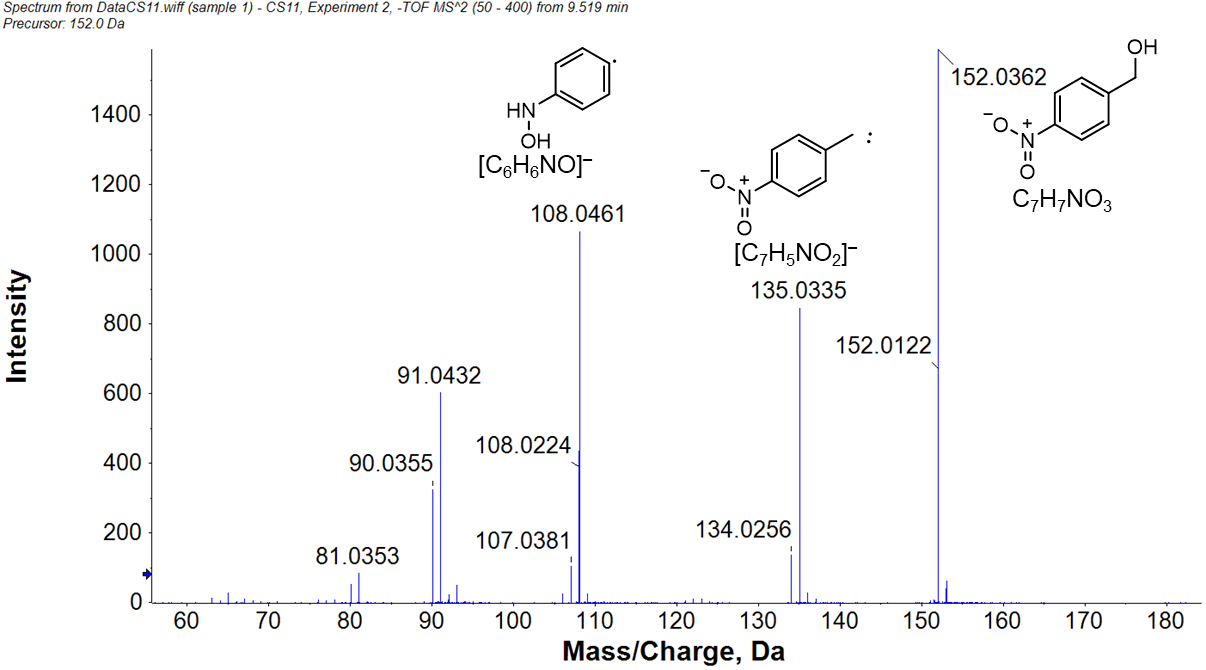


**Figure S25.** LC–MS/MS spectrum of (4-nitrophenyl)methanol (TP-152b).


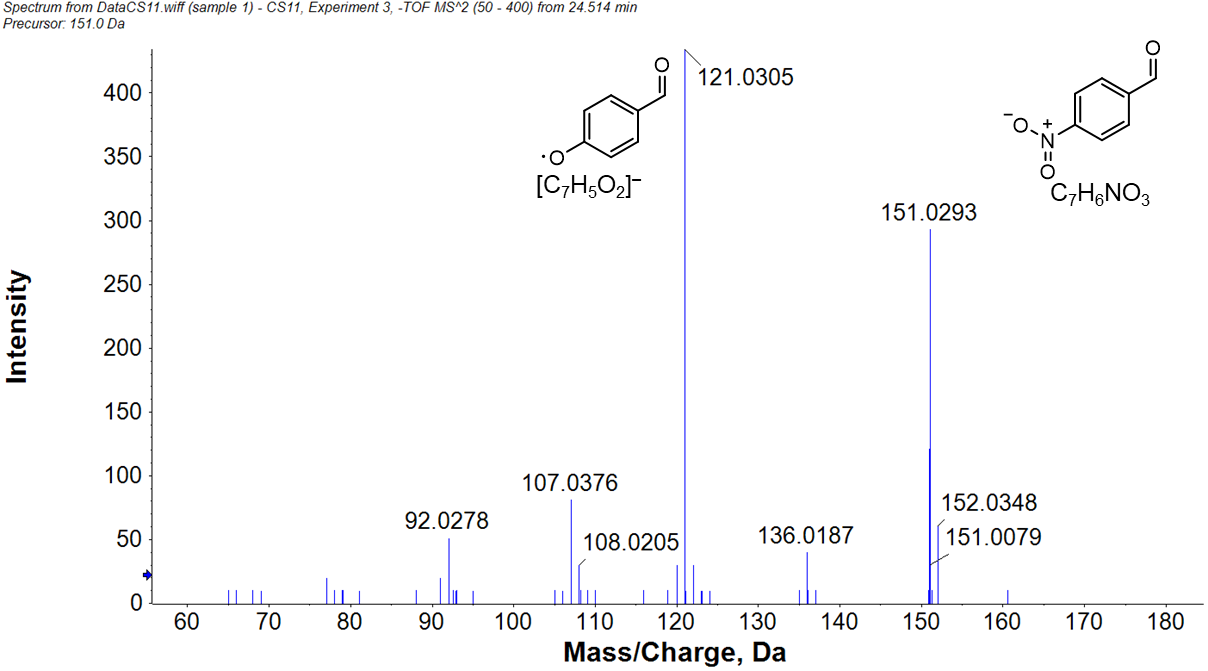


**Figure S26.** LC–MS/MS spectrum of 4-nitrobenzaldehyde (TP-151).


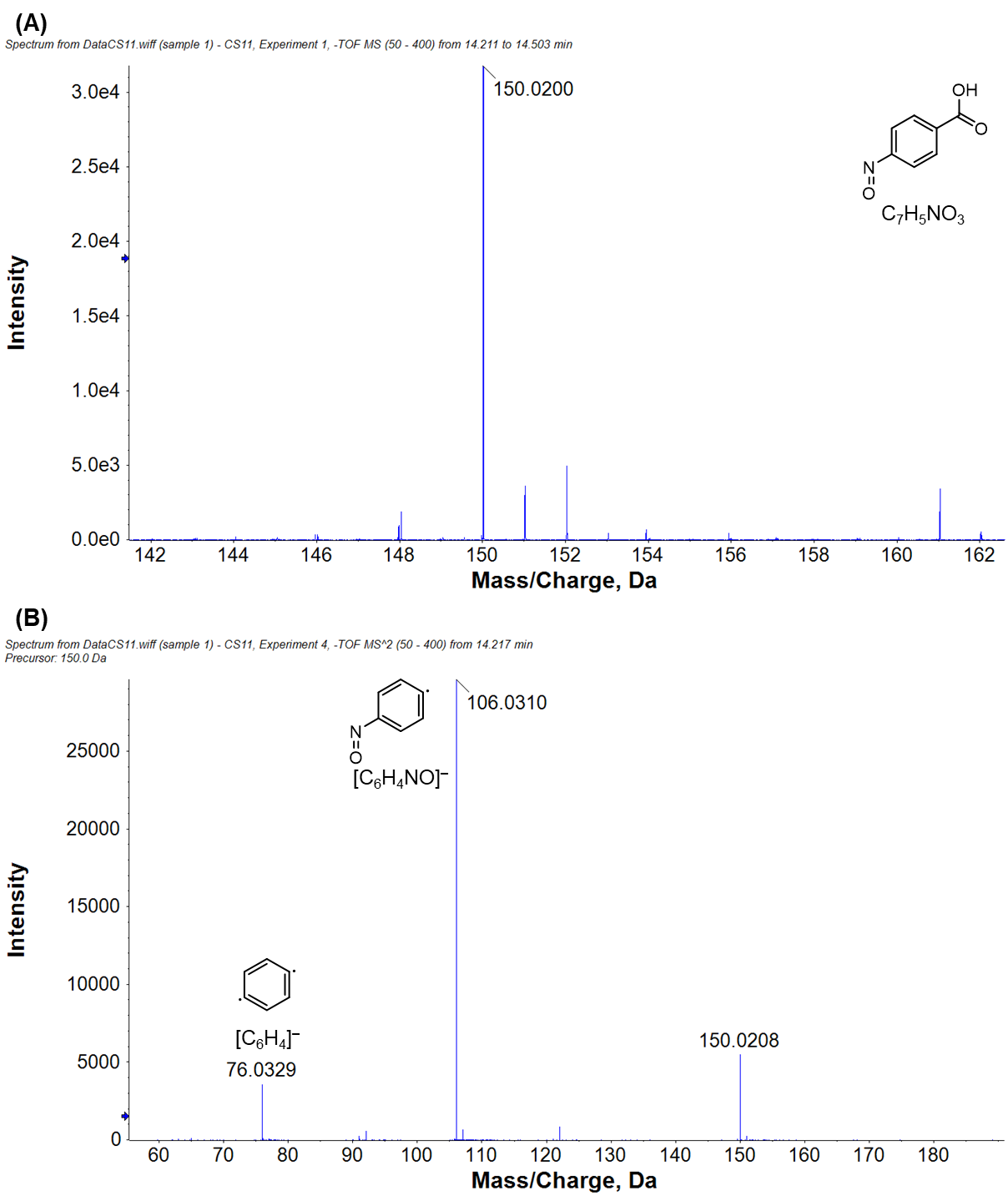


**Figure S27.** LC–MS **(A)** and LC–MS/MS **(B)** spectra of 4-nitrosobenzoic acid (TP-150).


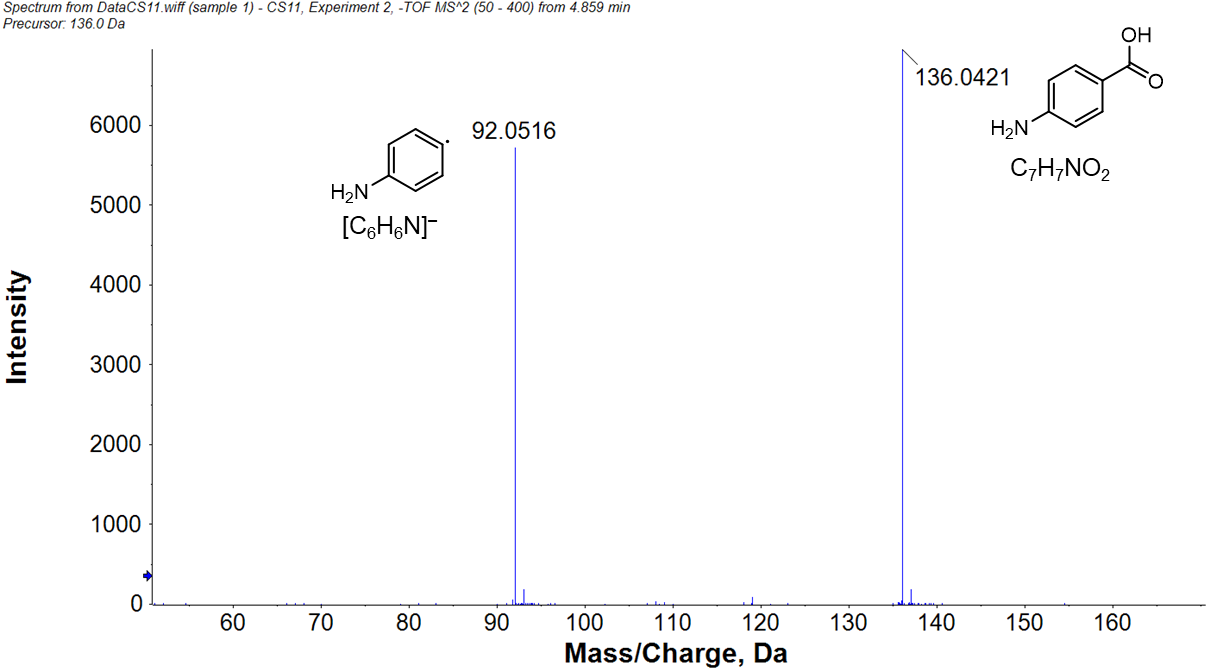


**Figure S28.** LC–MS/MS spectrum of 4-aminobenzoic acid (TP-136).


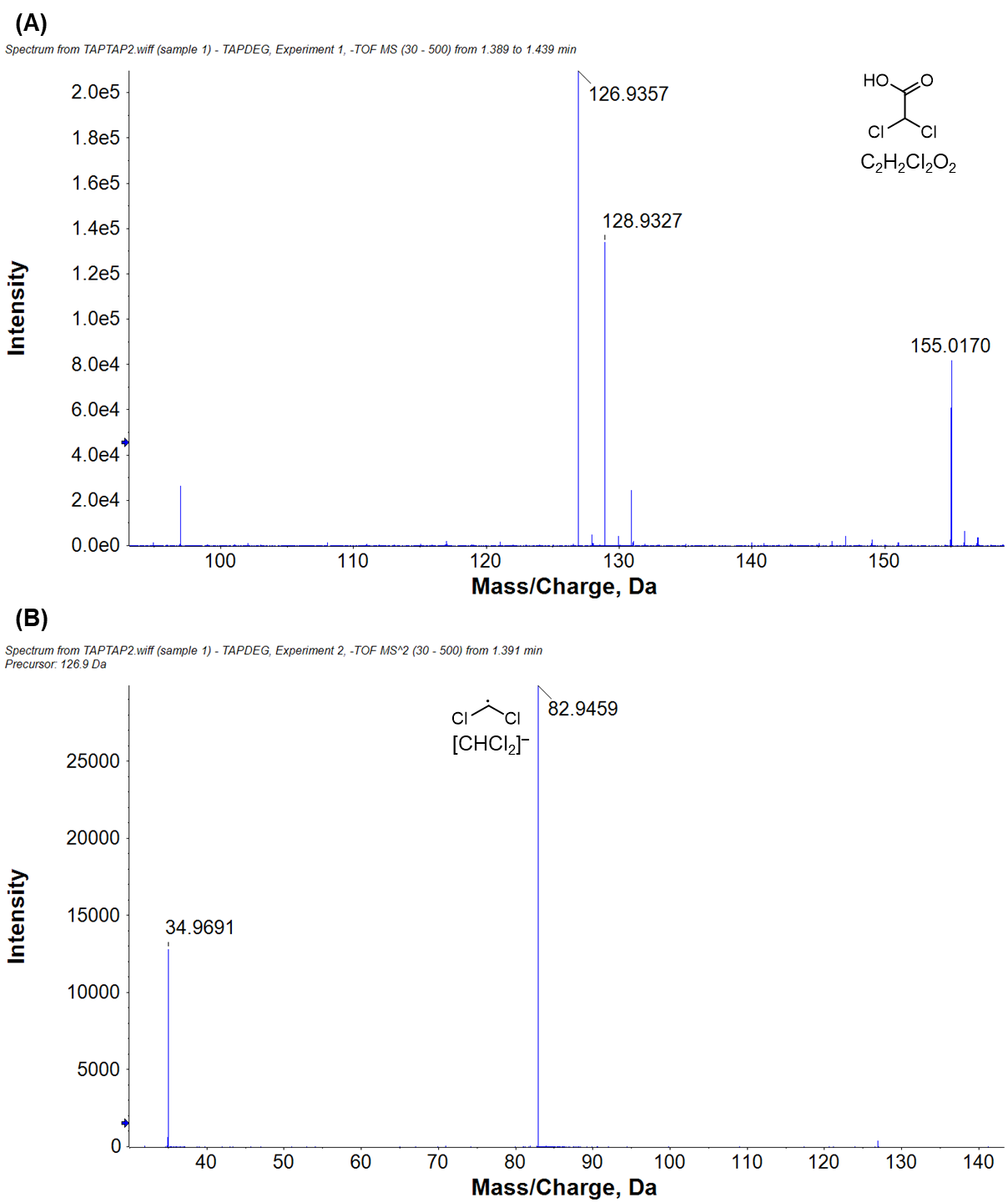


**Figure S29.** LC–MS **(A)** and LC–MS/MS **(B)** spectra of 2,2-dichloroacetic acid (TP-127).


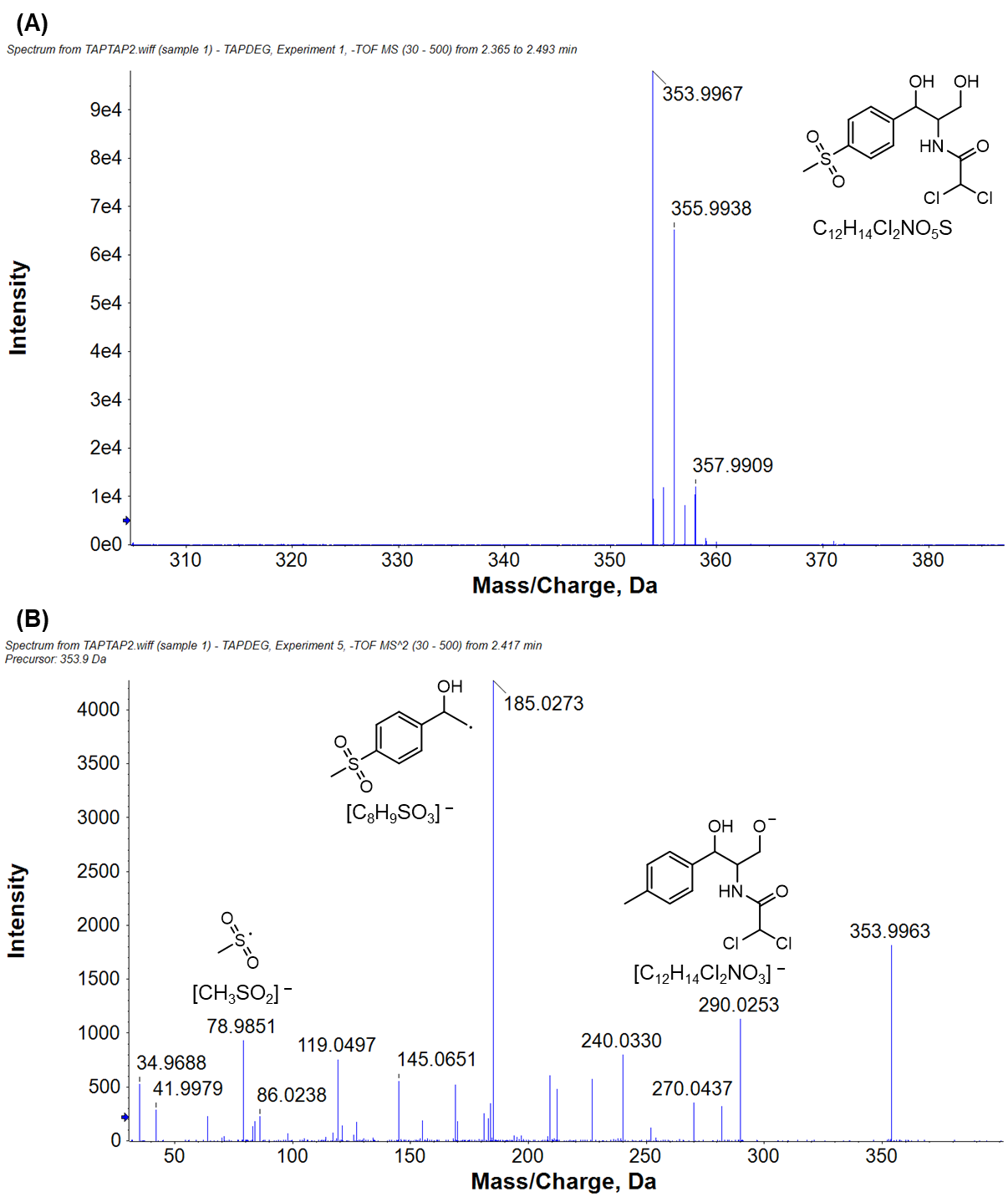


**Figure S30.** LC–MS **(A)** and LC–MS/MS **(B)** spectra of thiamphenicol (TAP).


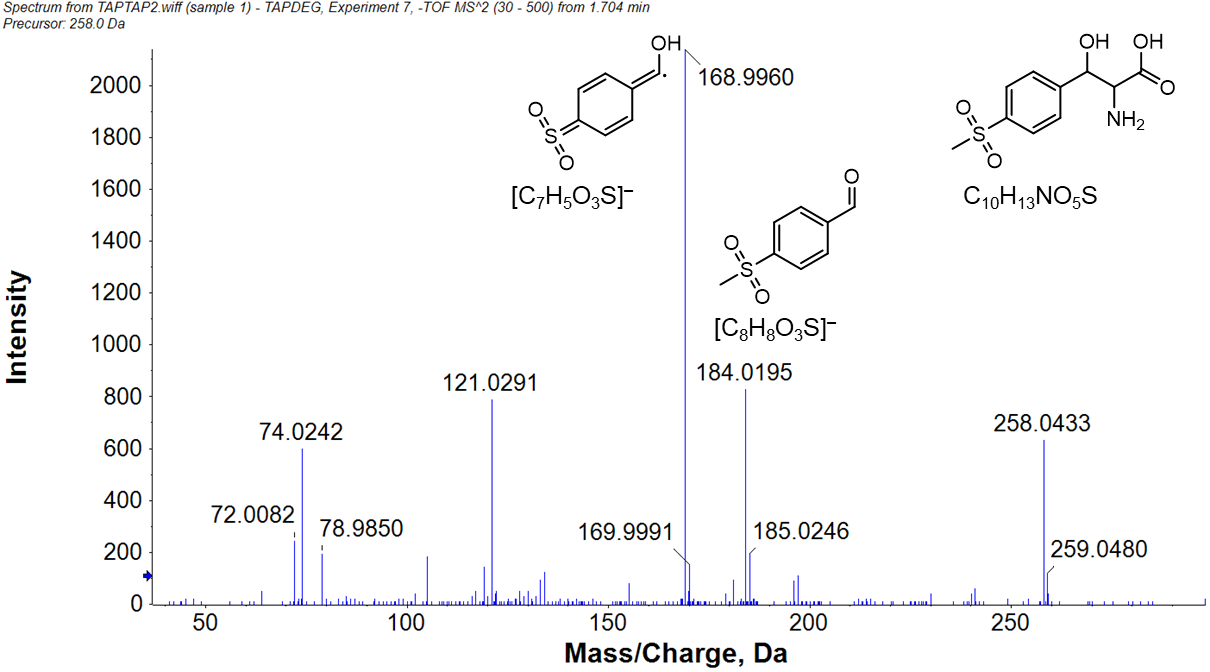


**Figure S31.** LC–MS/MS spectrum of 2-amino-3-hydroxy-3-(4-methylsulfonylphenyl)propionic acid (TAP-TP-258).


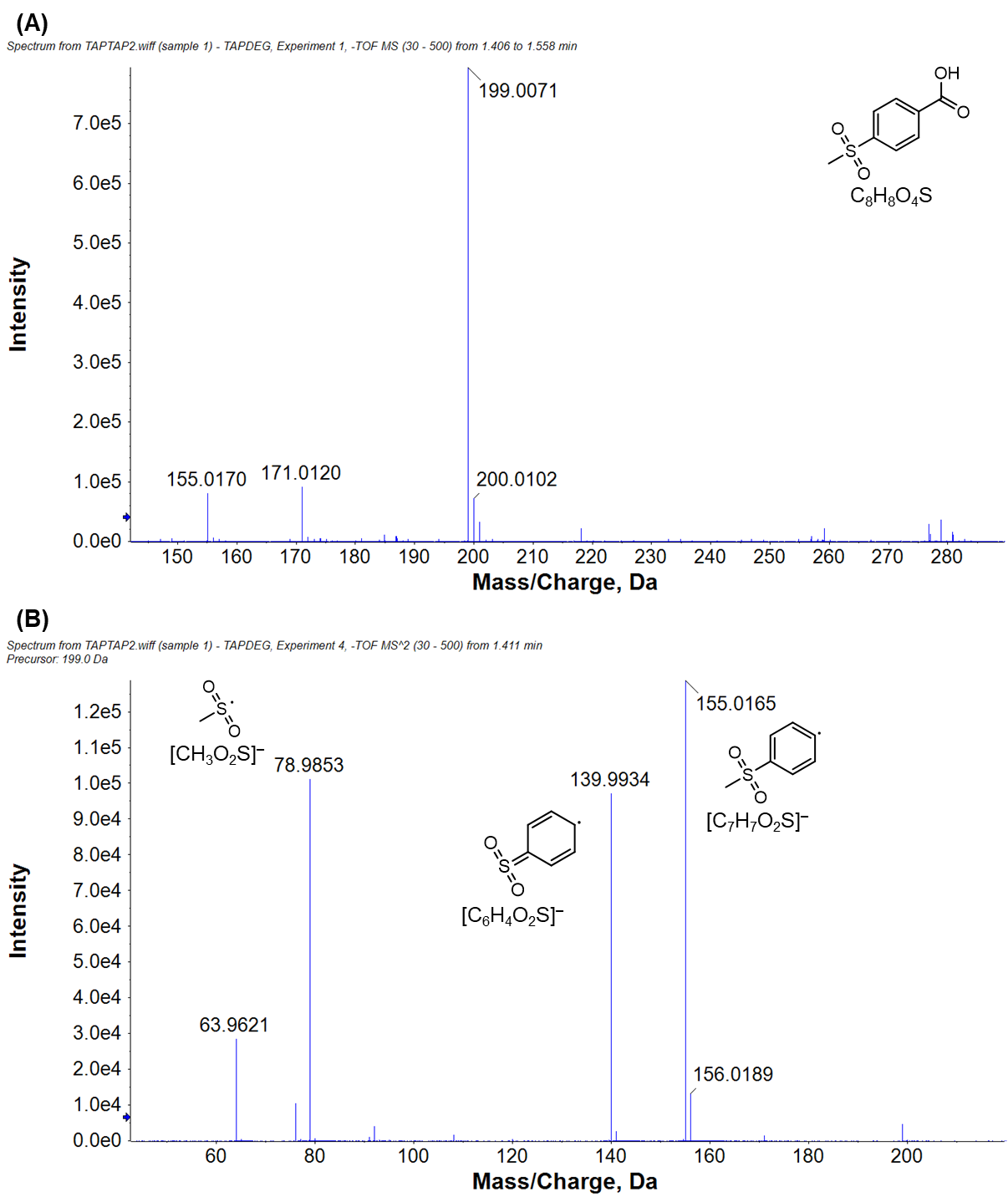


**Figure S32.** LC–MS **(A)** and LC–MS/MS **(B)** spectra of 4-methylsulfonylbenzoic acid (TAP-TP-199).


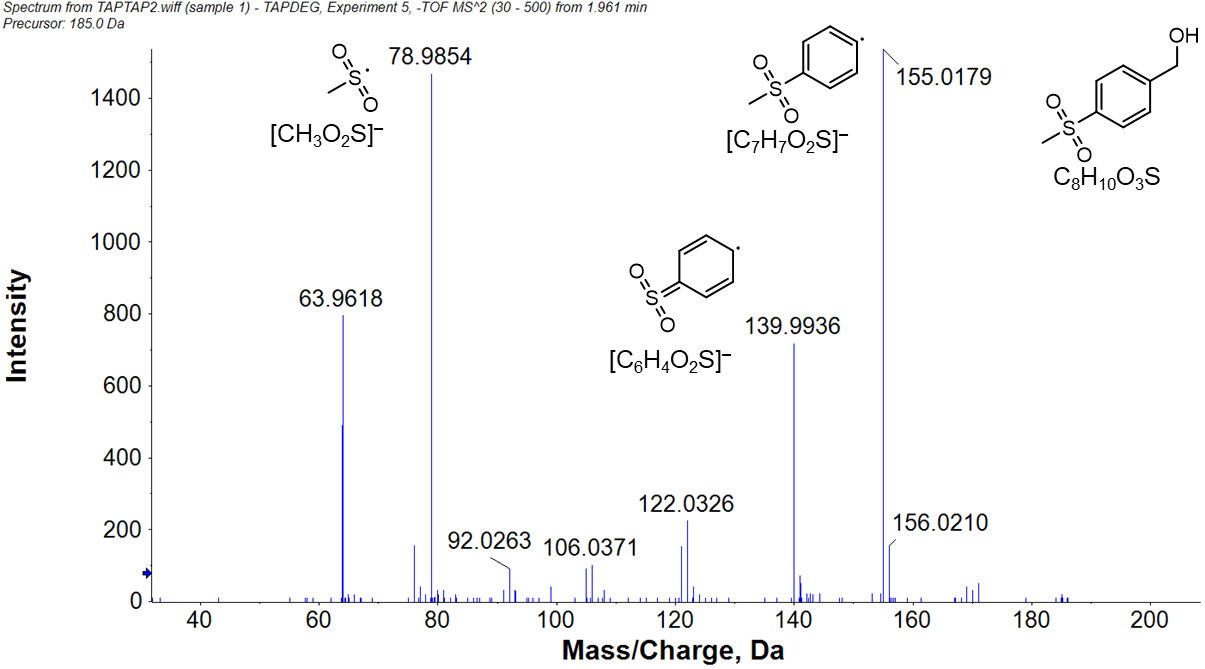


**Figure S33.** LC–MS/MS spectrum of (4-methylsulfonylphenyl)methanol (TAP-TP-185).


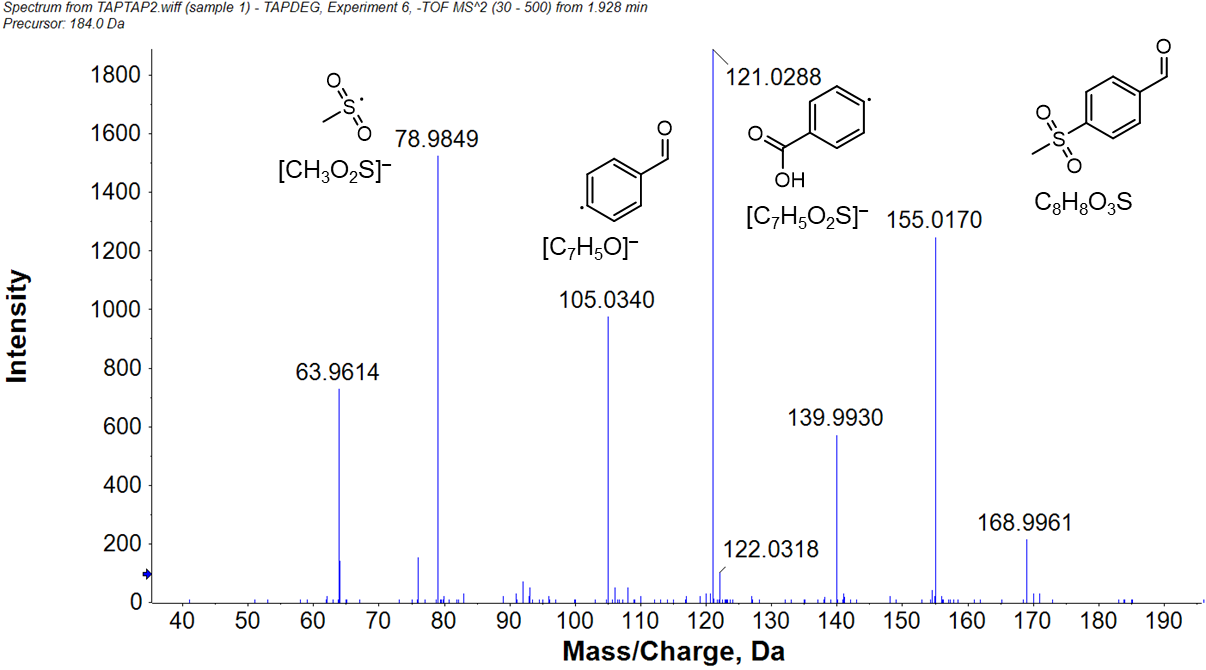


**Figure S34.** LC–MS/MS spectrum of 4-methylsulfonylbenzaldehyde (TAP-TP-184).


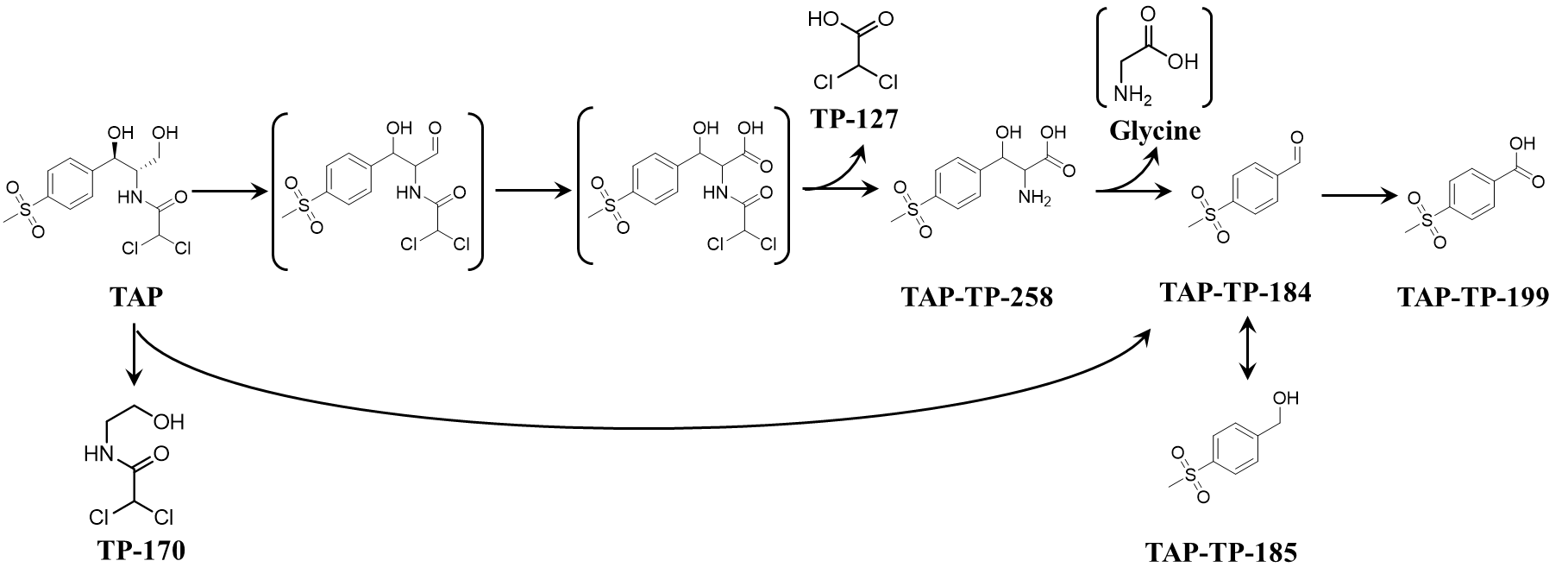


**Figure S35.** The proposed catabolism pathways of TAP by CS1. The compounds in brackets were not detected and represented the hypothetical intermediates.


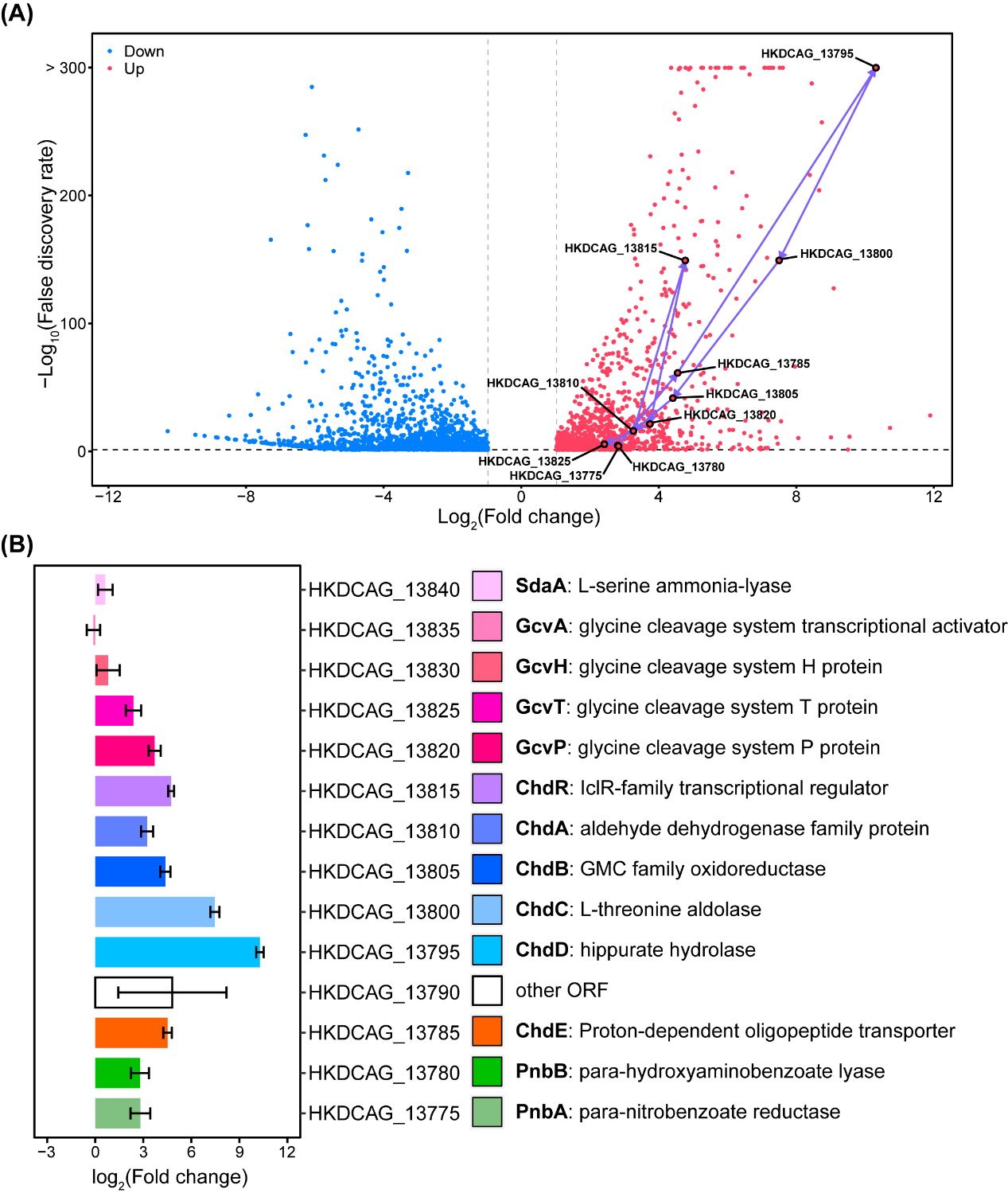


**Figure S36.** Transcriptome of CS1 under glucose and CAP treatment. (**A**) Volcano plots indicating the pairwise comparison of significantly expressed genes of CS1 between CAP and glucose treatments. (**B**) The log_2_(Fold change) of the CAP-degrading cluster genes in CAP treatment compared to glucose treatment. The expression fold change was determined based on the normalized counts between the indicated carbon substrates. Log_2_(Fold change) > 1 or < −1 (*p* < 0.05) indicated genes significantly up-regulated or down-regulated, respectively. Data are means of three individual experiments ± one standard deviation.


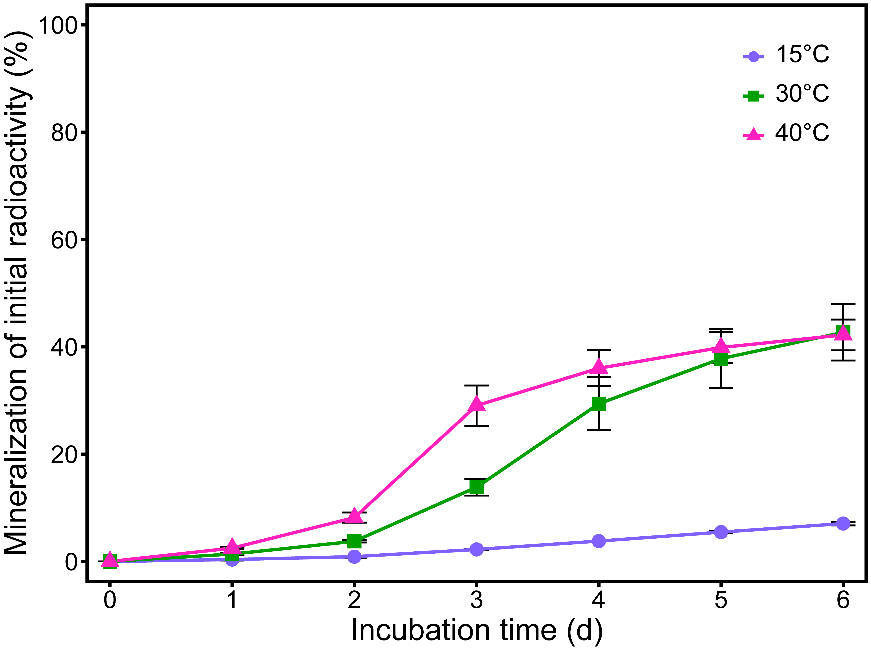


**Figure S37.** Mineralization of ^14^C-CAP in the Suzhou (SZ) soil at 15°C, 30°C, and 40°C.


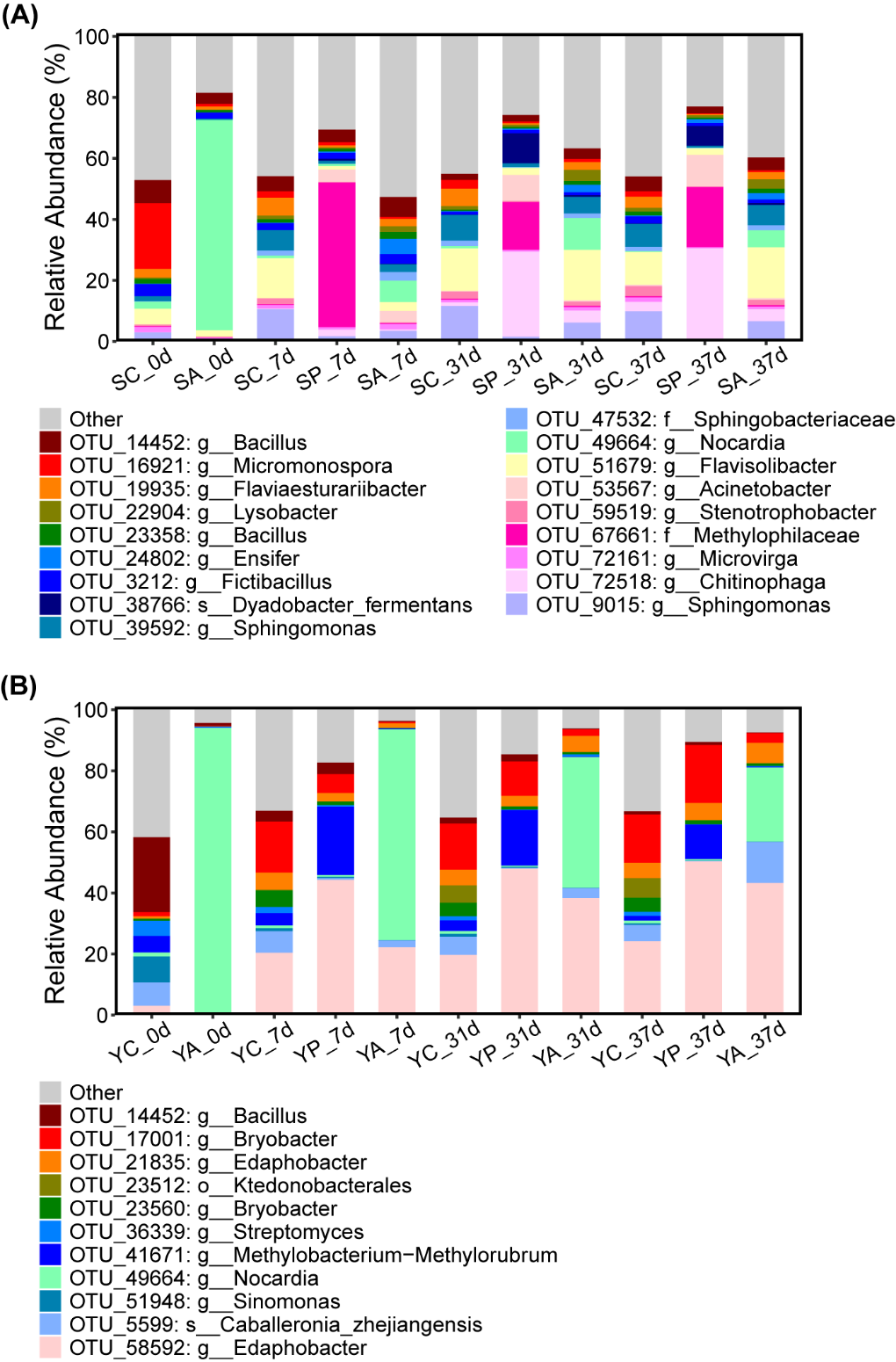


**Figure S38.** Bacterial community composition of the SZ soil (**A**) and the Yingtan (YT) soil (**B**) at 0, 7, 31, and 37 days. S and Y represent SZ and YT soils. A, C, and P represent CAP pollution with CS1 augmentation, no pollution, and CAP pollution treatments, respectively.


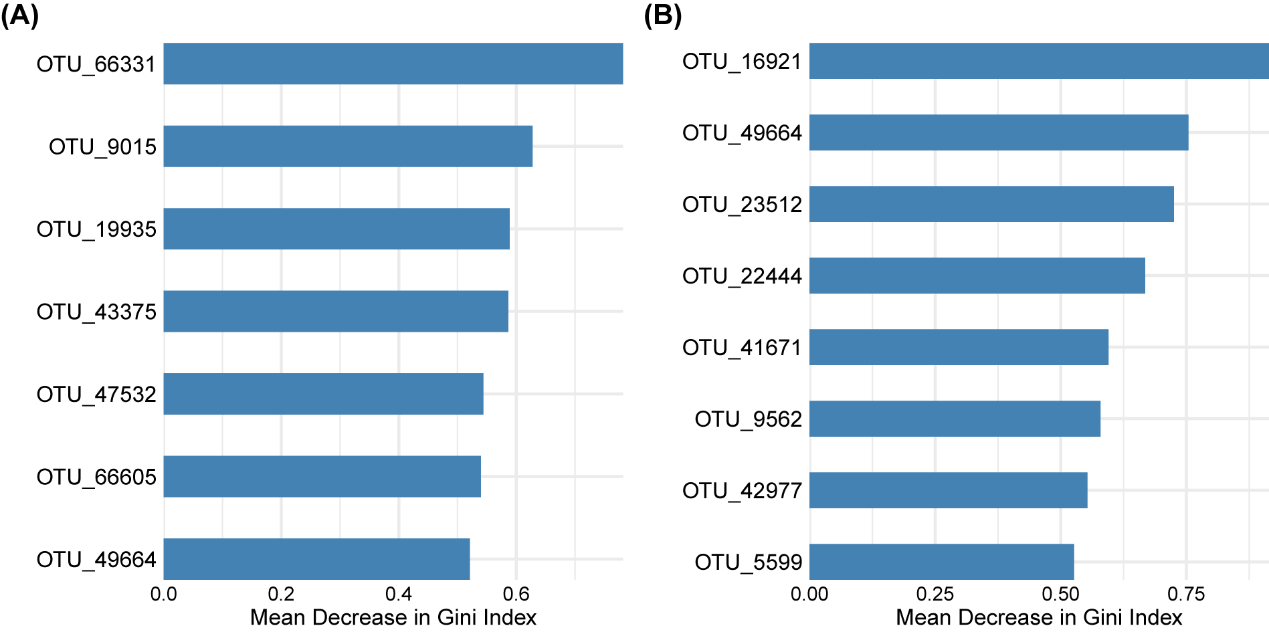


**Figure S39.** OTUs that caused a mean decrease in Gini index greater than 0.5 in random forests analysis in the bacterial communities of SZ (**A**) and YT (**B**) soils.


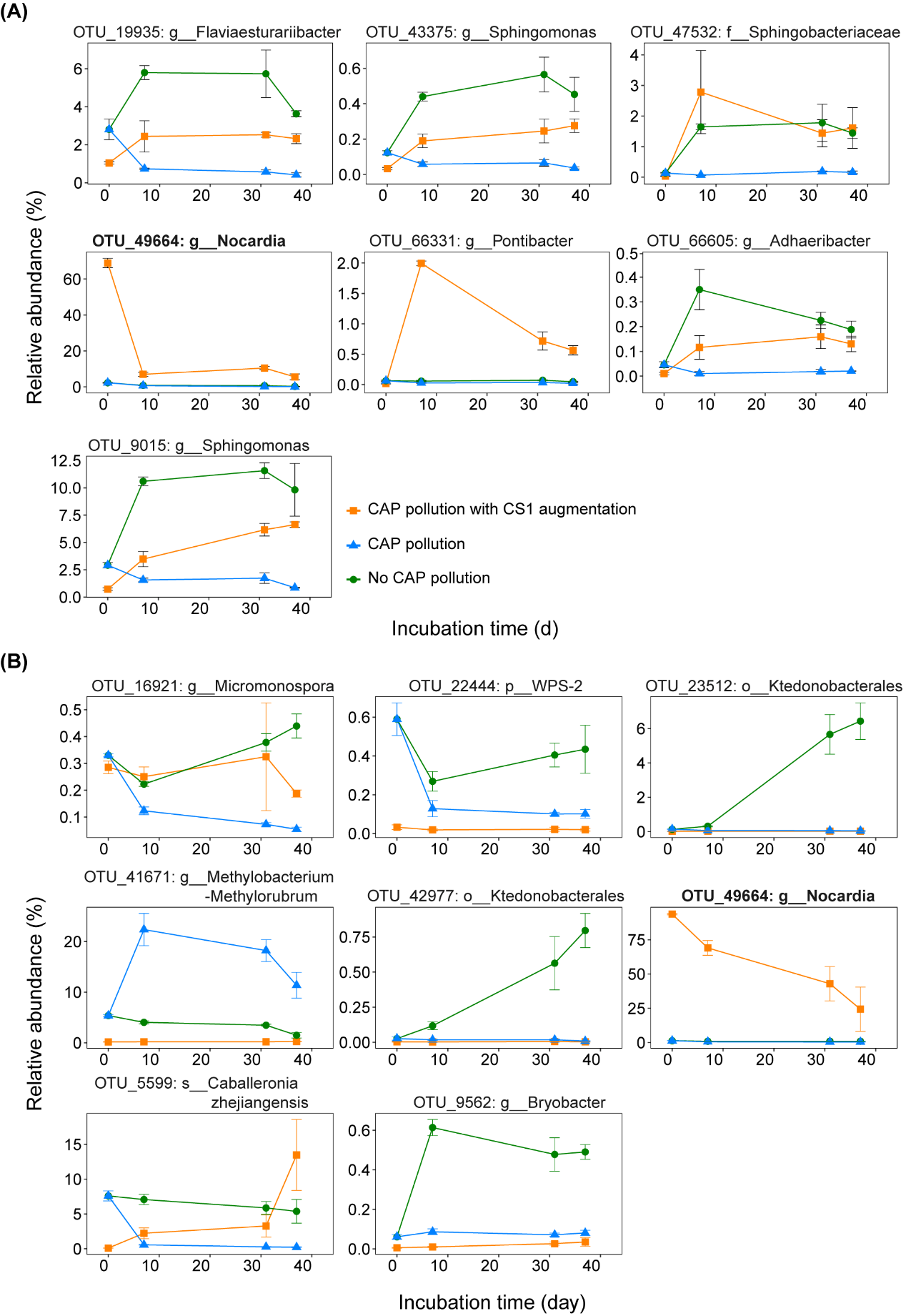


**Figure S40.** Relative abundance of important OTUs (mean decrease in Gini index > 0.5) in the soil bacterial communities of SZ (**A**) and YT (**B**) soils during the incubation period.


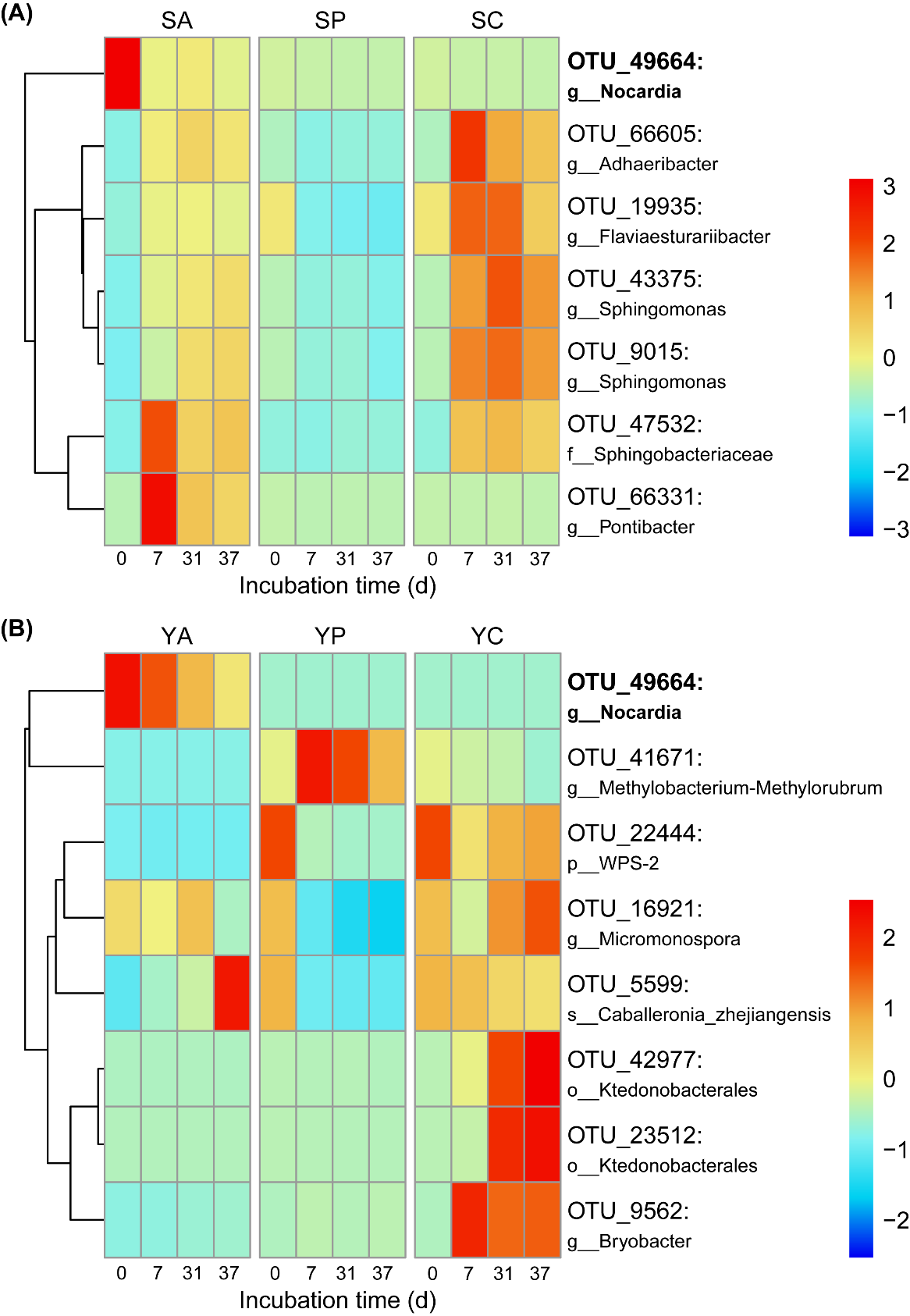


**Figure S41.** Relative abundance of important OTUs (mean decrease in Gini index > 0.5 in random forest analysis) after normalization using Z-scores in the soil bacterial communities of SZ (A) and YT (B) during the incubation. SC: SZ soil without CAP pollution; SP: SZ soil with CAP pollution; SA: SZ soil with CAP pollution and CS1 augmentation; YC: YT soil without CAP pollution; YP: YT soil with CAP pollution; YA: YT soil with CAP pollution and CS1 augmentation.


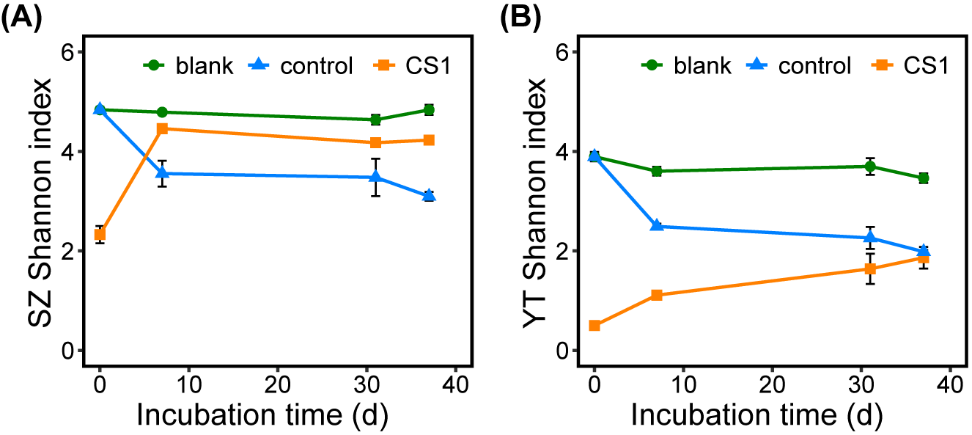


**Figure S42.** Shannon index of bacterial communities in SZ (**A**) and YT (**B**) soils. Blank: the pristine soil without CAP contamination. Control: CAP-polluted soil in the absence of CS1. CS1: CAP-polluted soil with CS1 bioaugmentation.


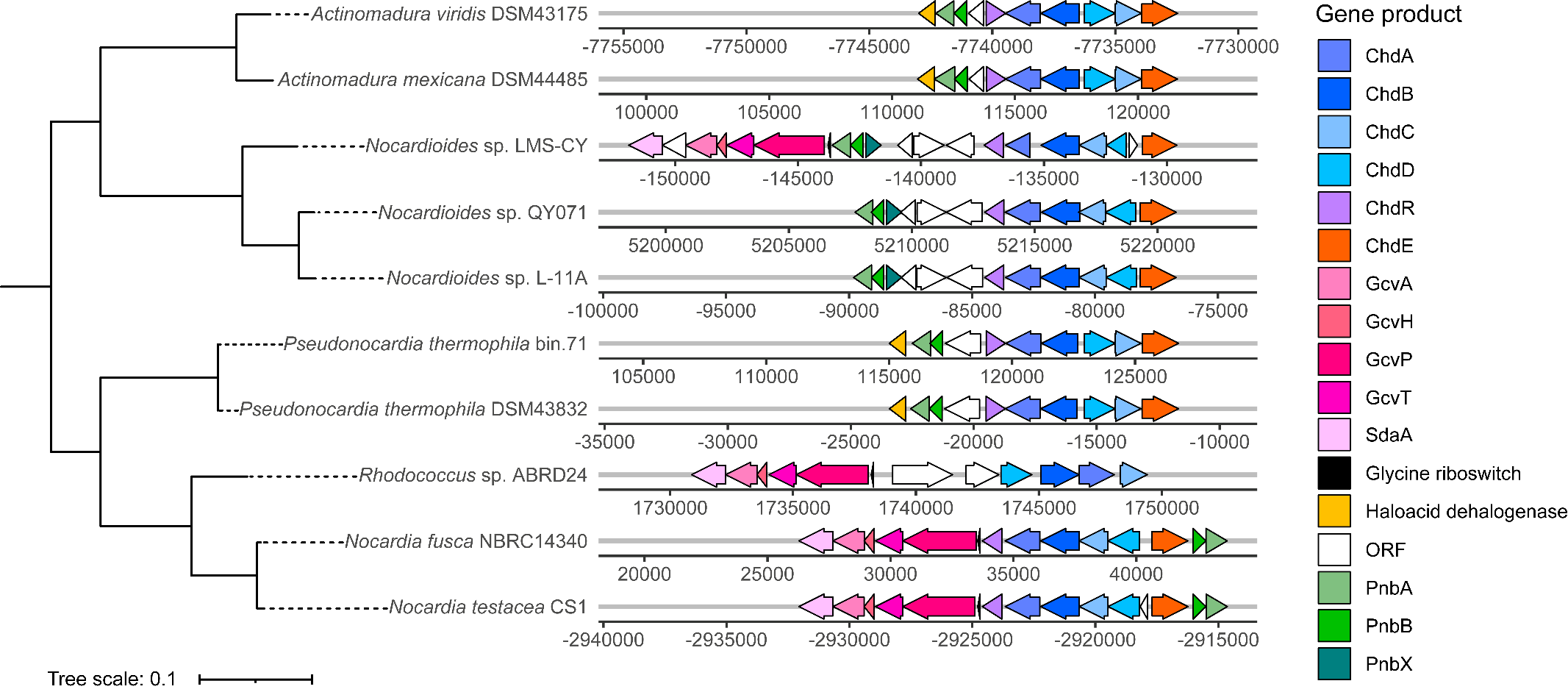


**Figure S43.** Phylogenetic tree of the strains carrying *chd* cluster. The accession numbers were shown in Table S6. The functions of gene products: **ChdA** (aldehyde dehydrogenase family protein); **ChdB** (GMC family oxidoreductase); **ChdC** (L-threonine aldolase); **ChdD** (hippurate hydrolase); **ChdE** (Proton-dependent oligopeptide transporter); **ChdR** (IclR-family transcriptional regulator); **GcvA** (glycine cleavage system transcriptional activator); **GcvH** (glycine cleavage system H protein); **GcvP**: (glycine cleavage system P protein); **GcvT** (glycine cleavage system T protein); **SdaA** (L-serine ammonia-lyase); ORF (unannotated open reading frame); **PnbA** (4-nitrobenzoate reductase); **PnbB** (4-hydroxyaminobenzoate lyase); **PnbX** (4-nitrobenzoate-responsive repressor).


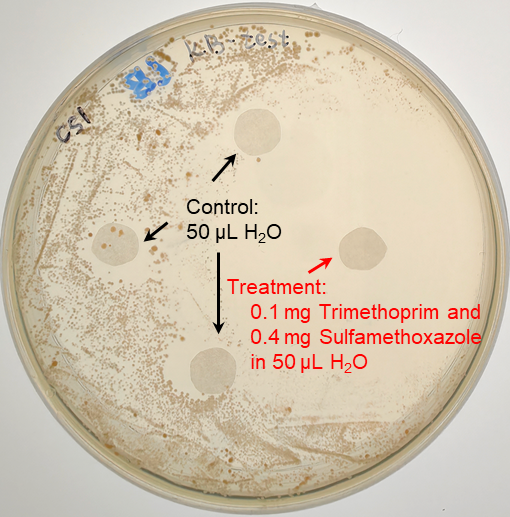


**Figure S44.** Antibiotic susceptibility of strain CS1 determined by Kirby–Bauer disk diffusion assay using trimethoprim–sulfamethoxazole.

# TABLES

**Table S1.** Overview of reported chloramphenicol (CAP) degrading bacterial isolates. Their genome accession numbers are provided in Table S2.

| **Phylum** | **Strain** | **Habitat** | **CAP concentration** | **Sole carbon source** | **Removal efficiency** | **Reference** |
| --- | --- | --- | --- | --- | --- | --- |
| Pseudomonadota | *Sphingomonas* sp. CL5.1 | Activated sludge | 60 mg/L | Yes | 100% removal in 36 h | [[10](#_ENREF_10)] |
| Pseudomonadota | *Sphingobium* sp. CAP-1 | Activated sludge | 50 mg/L | Yes | 100% removal in 48 h | [[11](#_ENREF_11)] |
| Pseudomonadota | *Sphingobium* sp. WTD-1 | Activated sludge | 50 mg/L | Yes | 100% removal in 60 h | [[12](#_ENREF_12)] |
| Pseudomonadota | *Klebsiella pneumoniae*  I-11-CHL-1 | Fecal samples from human | 20 mg/L | Yes | 10% removal in 13 d | [[13](#_ENREF_13)] |
| Pseudomonadota | *Aeromonas media* SZW3 | Earthworm gut | 50 mg/L | No | 15% removal in 1 d | [[14](#_ENREF_14)] |
| Pseudomonadota | *Klebsiella* sp. YB1 | Earthworm gut | 20 mg/L | No | 22% removal in 2 d | [[15](#_ENREF_15)] |
| Actinomycetota | *Nocardiopsis synnemataformans*  DSM 44143 | Sputum of a patient | 12 mg/L | No | 100% removal in 125 h | [[16](#_ENREF_16)] |
| Actinomycetota | *Nocardioides* sp. QY071 | Chicken faeces | 25 mg/L | Yes | 100% removal in 2 h | [[17](#_ENREF_17)] |
|  | *Nocardioides* sp. L-11A | Soil | 25 mg/L | Yes | 100% removal in 4 h |  |
|  | *Nocardioides* sp. LMS-CY | Activated sludge | 25 mg/L | Yes | 100% removal in 2 h |  |

**Table S2.** The physiochemical properties of Suzhou (SZ) and Yingtan (YT) soils.

| **Soil** | **SZ** | **YT** |
| --- | --- | --- |
| Geographical location | Jiangsu Province, P. R. China | Jiangxi Province, P. R. China |
| Type | Paddy soils | Red earths |
| Soil organic matter (g/kg) | 33 | 15 |
| Total nitrogen (g/kg) | 2.2 | 0.6 |
| Cationic exchange capacity (cmol/kg) | 23.4 | 5.2 |
| pH | 7.30 | 4.43 |

**Table S3.** Strains carrying *chd* cluster, their isolation sources, and accession numbers.

| **Strain** | **Isolation source** | **Accession number** |
| --- | --- | --- |
| *Nocardia testacea* CS1 | Soil | GCF_048019105.1 |
| *Nocardia fusca* NBRC 14340 | Soil | GCF_001618425.1 |
| *Rhodococcus* sp. ABRD24 | Freshwater sediment | GCF_004328705.1 |
| *Actinomadura mexicana* DSM 44485 | Soil | GCF_900188105.1 |
| *Actinomadura viridis* DSM 43175 | Soil | GCF_015751755.1 |
| *Pseudonocardia thermophila* bin.71 | Rice straw compost enrichment | GCF_937930745.1 |
| *Pseudonocardia thermophila* DSM 43832 | Fresh horse manure | GCF_900142365.1 |
| *Nocardioides* sp. QY071 | Livestock manure | GCF_029961765.1 |
| *Nocardioides* sp. L-11A | Soil | GCF_029961745.1 |
| *Nocardioides* sp. LMS-CY | Sludge | GCF_018732145.1 |

**Table S4.** Genes of protocatechuic acid downstream metabolism in strains CS1.

| **Sequence ID** | **Type** | **Start** | **Stop** | **Strand** | **Locus Tag** | **Gene** |
| --- | --- | --- | --- | --- | --- | --- |
| contig_1 | cds | 4440247 | 4440969 | + | HKDCAG_20630 | *pcaH* |
| contig_1 | cds | 4440962 | 4441588 | + | HKDCAG_20635 | *pcaG* |
| contig_1 | cds | 4441598 | 4442950 | + | HKDCAG_20640 | *pcaB* |
| contig_1 | cds | 4442993 | 4444222 | + | HKDCAG_20645 | *pcaD* |
| contig_1 | cds | 4444219 | 4444998 | + | HKDCAG_20650 | *pcaR* |

**Table S5.** Genes carried by CS1 against environmental stress.

| **Sequence ID** | **Type** | **Start** | **Stop** | **Strand** | **Locus tag** | **Gene product** |
| --- | --- | --- | --- | --- | --- | --- |
| contig_1 | cds | 64016 | 64570 | - | HKDCAG_00285 | Copper resistance protein CopC |
| contig_1 | cds | 317417 | 317623 | + | HKDCAG_01425 | Cold shock protein, CspA family |
| contig_1 | cds | 612166 | 612585 | + | HKDCAG_02910 | Cold shock protein, CspA family |
| contig_1 | cds | 2502435 | 2502866 | + | HKDCAG_11835 | Chromate resistance protein |
| contig_1 | cds | 3442252 | 3442680 | - | HKDCAG_16090 | Organic hydroperoxide resistance protein |
| contig_1 | cds | 3647035 | 3647349 | + | HKDCAG_17030 | Arsenate reductase |
| contig_1 | cds | 3647346 | 3647789 | + | HKDCAG_17035 | Mercuric resistance operon regulatory protein |
| contig_1 | cds | 3691008 | 3692984 | - | HKDCAG_17245 | cellulose biosynthesis cyclic di-GMP-binding regulatory protein BcsB |
| contig_1 | cds | 5092035 | 5092238 | + | HKDCAG_23600 | Cold shock protein |
| contig_1 | cds | 5634413 | 5634658 | - | HKDCAG_26120 | Cold shock protein, CspA family |

**Table S6.** CAP-degrading strains carrying genes *chdABCD*, and their genome accession numbers.

| **Strain** | **Genome accession number** |
| --- | --- |
| *Nocardia testacea* CS1 | GCF_048019105.1 |
| *Nocardioides* sp. LMS-CY | GCF_018732145.1 |
| *Nocardioides* sp. QY071 | GCF_029961765.1 |
| *Nocardioides* sp. L-11A | GCF_029961745.1 |
| *Sphingobium* sp. CAP-1  *Sphingomonas* sp. CL5.1  *Caballeronia* sp. PC1 | GCF_009720145.1  GCF_013344685.1  GCF_021353155.1 |

**Table S7.** Genes in the CS1 genome encoding protein homologous to Virulence Factor Database (VFDB).

| **Gene locus tag** | **Gene accession in VFDB (in Genbank)** | **Protein sequence matching region (%)** | **Coverage (%)** | **Annotation in VFDB** |
| --- | --- | --- | --- | --- |
| HKDCAG_29085 | VFG001392(gb\|NP_215737) | 87.5 | 72.6 | ECF RNA polymerase sigma factor SigE [SigE (VF0295) - Regulation (VFC0301)] |
| HKDCAG_30520 | VFG001390(gb\|NP_215496) | 85.5 | 99.6 | two-component response regulator MrpA [MprAB (VF0298) - Regulation (VFC0301)] |
| HKDCAG_08800 | VFG001404(gb\|NP_216944) | 84.5 | 99.5 | alkyl hydroperoxide reductase subunit AhpC [AhpC (VF0306) - Stress survival (VFC0282)] |
| HKDCAG_24435 | VFG001826(gb\|NP_217099) | 83.8 | 93.0 | Probable GTP pyrophosphokinase RelA [RelA (VF0287) - Regulation (VFC0301)] |
| HKDCAG_31880 | VFG001381(gb\|YP_177728) | 82.8 | 100.0 | Isocitrate lyase Icl [Isocitrate lyase (VF0253) - Others (VFC0346)] |
| HKDCAG_28265 | VFG001412(gb\|NP_217739) | 80.4 | 98.1 | redox-responsive transcriptional regulator WhiB3 [WhiB3 (VF0288) - Regulation (VFC0301)] |
| HKDCAG_00345 | VFG001421(gb\|NP_218363) | 80.2 | 99.5 | superoxide dismutase [SodA (VF0304) - Stress survival (VFC0282)] |
| HKDCAG_02470 | VFG001818(gb\|NP_216893) | 78.9 | 98.6 | putative protein MbtH [Mycobactin (VF0299) - Nutritional/Metabolic factor (VFC0272)] |
| HKDCAG_02490 | VFG001386(gb\|NP_215271) | 77.9 | 93.9 | flagellar basal body protein FliL [Flagella (VF0430) - Motility (VFC0204)] |
| HKDCAG_01715 | VFG001416(gb\|NP_218118) | 75.6 | 98.5 | aspartate 1-decarboxylase [PanC/PanD (VF0319) - Nutritional/Metabolic factor (VFC0272)] |
| HKDCAG_20945 | VFG001824(gb\|NP_217649) | 75.0 | 100.0 | two component transcriptional regulator DevR [DevRS (VF0317) - Regulation (VFC0301)] |
| HKDCAG_25050 | VFG001406(gb\|NP_217227) | 73.9 | 98.7 | ferrienterobactin ABC transporter ATPase [Enterobactin (VF0228) - Nutritional/Metabolic factor (VFC0272)] |
| HKDCAG_31135 | VFG046465(gb\|WP_003028672) | 72.5 | 100.3 | elongation factor Tu [EF-Tu (VF0460) - Adherence (VFC0001)] |
| HKDCAG_14155 | VFG001824(gb\|NP_217649) | 70.7 | 100.0 | two component transcriptional regulator DevR [DevRS (VF0317) - Regulation (VFC0301)] |
| HKDCAG_32900 | VFG001379(gb\|NP_214867) | 70.4 | 91.2 | heat shock protein transcriptional repressor HspR [HspR (VF0297) - Regulation (VFC0301)] |

**Table S8.** Genes in the CS1 genome with >70% protein sequence identity of matching region to reference sequences in VFDB.

| **ARO term** | **AMR gene family** | **Drug class** | **Resistance mechanism** | **% identity of matching region** | **% Length of reference sequence** |
| --- | --- | --- | --- | --- | --- |
| Streptomyces venezuelae rox | rifampin monooxygenase | rifamycin antibiotic | antibiotic inactivation | 72.73 | 103.36 |
| Mycobacterium tuberculosis rpoC mutations confer resistance to rifampicin | rifampicin resistant rpoC | rifamycin antibiotic | antibiotic target alteration | 88.54 | 100.08 |

# REFERENCES

[1] S. Huang; Q. Wang; Z. Fan; M. Xu; R. Ji; X. Jin; et al. Dry-to-wet fluctuation of moisture contents enhanced the mineralization of chloramphenicol antibiotic, Water Res. 240 (2023) 120103.

[2] R. Lu. Analytical methods of soil agrochemistry, China Agricultural Science and Technology Press, Beijing (1999) 85−96.

[3] M. R. Carter; E. G. Gregorich. *Soil sampling and methods of analysis*; CRC press, 2007.

[4] H. Cheng; G. T. Concepcion; X. Feng; H. Zhang; H. Li. Haplotype-resolved de novo assembly using phased assembly graphs with hifiasm, Nat. Methods 18 (2021) 170−175.

[5] M. Hunt; N. D. Silva; T. D. Otto; J. Parkhill; J. A. Keane; S. R. Harris. Circlator: Automated circularization of genome assemblies using long sequencing reads, Genome Biol. 16 (2015) 294.

[6] B. J. Walker; T. Abeel; T. Shea; M. Priest; A. Abouelliel; S. Sakthikumar; et al. Pilon: An integrated tool for comprehensive microbial variant detection and genome assembly improvement, PLoS One 9 (2014) e112963.

[7] S. Chen; Y. Zhou; Y. Chen; J. Gu. fastp: An ultra-fast all-in-one FASTQ preprocessor, Bioinformatics 34 (2018) i884−i890.

[8] D. Kim; B. Langmead; S. L. Salzberg. HISAT: A fast spliced aligner with low memory requirements, Nat. Methods 12 (2015) 357−360.

[9] E. Bolyen; J. R. Rideout; M. R. Dillon; N. A. Bokulich; C. C. Abnet; G. A. Al-Ghalith; et al. Reproducible, interactive, scalable and extensible microbiome data science using QIIME 2, Nat. Biotechnol. 37 (2019) 852−857.

[10] J. Zhang; W. Gan; R. Zhao; K. Yu; H. Lei; R. Li; et al. Chloramphenicol biodegradation by enriched bacterial consortia and isolated strain *Sphingomonas* sp. CL5.1: The reconstruction of a novel biodegradation pathway, Water Res. 187 (2020) 116397.

[11] X. Ma; B. Liang; M. Qi; H. Yun; K. Shi; Z. Li; et al. Novel pathway for chloramphenicol catabolism in the activated sludge bacterial isolate *Sphingobium* sp. CAP-1, Environ. Sci. Technol. 54 (2020) 7591−7600.

[12] Y. Gao; Y. Chen; F. Zhu; D. Pan; J. Huang; X. Wu. Revealing the biological significance of multiple metabolic pathways of chloramphenicol by *Sphingobium* sp. WTD-1, J. Hazard. Mater. 469 (2024) 134069.

[13] Z. Xin; T. Fengwei; W. Gang; L. Xiaoming; Z. Qiuxiang; Z. Hao; et al. Isolation, identification and characterization of human intestinal bacteria with the ability to utilize chloramphenicol as the sole source of carbon and energy, FEMS Microbiol. Ecol. 82 (2012) 703−712.

[14] Z. Tan; X. Yang; L. Chen; Y. Liu; H.-J. Xu; Y. Li; et al. Biodegradation mechanism of chloramphenicol by *Aeromonas media* SZW3 and genome analysis, Bioresour. Technol. 344 (2022) 126280.

[15] Z. Tan; X. Yang; Y. Liu; L. Chen; H. Xu; Y. Li; et al. The capability of chloramphenicol biotransformation of *Klebsiella* sp. YB1 under cadmium stress and its genome analysis, Chemosphere 313 (2023) 137375.

[16] L. Zhang; M. Toplak; R. Saleem-Batcha; L. Höing; R. Jakob; N. Jehmlich; et al. Bacterial dehydrogenases facilitate oxidative inactivation and bioremediation of chloramphenicol, ChemBioChem 24 (2023) e202200632.

[17] Y. Qian; M. Cheng; L. Lai; J. Zhou; G. J. Zylstra; X. Huang. ChlOR, a GMC family oxidoreductase that evolved independently from the actinomycete, confers resistance to amphenicol antibiotics, Environ. Microbiol. 25 (2023) 3019−3034.
